# Comparative study of the evolution of cancer gene duplications across fish

**DOI:** 10.1111/eva.13481

**Published:** 2022-09-29

**Authors:** Ciara Baines, Richard Meitern, Randel Kreitsberg, Tuul Sepp

**Affiliations:** ^1^ Institute of Ecology and Earth Sciences University of Tartu Tartu Estonia; ^2^ Estonian Marine Institute University of Tartu Tallinn Estonia

**Keywords:** cancer defense mechanisms, cancer evolution, fish cancer, fish evolution, tumor suppression, wildlife cancer

## Abstract

Comparative studies of cancer‐related genes not only provide novel information about their evolution and function but also an understanding of cancer as a driving force in biological systems and species’ life histories. So far, these studies have focused on mammals. Here, we provide the first comparative study of cancer‐related gene copy number variation in fish. Fishes are a paraphyletic group whose last common ancestor is also an ancestor of the tetrapods, and accordingly, their tumour suppression mechanisms should include most of the mammalian mechanisms and also reveal novel (but potentially phylogenetically older) previously undetected mechanisms. We have matched the sequenced genomes of 65 fish species from the Ensemble database with the cancer gene information from the COSMIC database. By calculating the number of gene copies across species using the Ensembl CAFE data (providing species trees for gene copy number counts), we used a less resource‐demanding method for homolog identification. Our analysis demonstrates a masked relationship between cancer‐related gene copy number variation (CNV) and maximum lifespan in fish species, suggesting that a higher number of copies of tumour suppressor genes lengthens and the number of copies of oncogenes shortens lifespan. Based on the positive correlation between the number of copies of tumour suppressors and oncogenes, we show which species have more tumour suppressors in relation to oncogenes. It could be suggested that these species have stronger genetic defences against oncogenic processes. Fish studies could be a largely unexplored treasure trove for understanding the evolution and ecology of cancer, providing novel insights into the study of cancer and tumour suppression, in addition to fish evolution, life‐history trade‐offs, and ecology.

## 1 INTRODUCTION

Cancer is a disease that arose with multicellularity and is caused by a variety of factors, including mutations that occur either somatically, arising throughout the organism's lifetime, or are inherited through the germline (Trigos et al., 2018). It is estimated that approximately 90% of mutations leading to cancer in humans are somatic (Sondka et al., 2018). Evolution, as a result, has led to the selection of genes that reduce the risk of an organism to neoplastic development. It is understood that oncogenes (OGs), tumour‐suppressor genes (TSGs), and differentiation genes are amongst the oldest gene classes in humans (Makashov et al., 2019), opening a possibility for gaining novel information about the evolution and function of these genes from comparative studies. Moreover, comparative studies not only allow us to understand that cancer is not only a disease but also a driving force in biological systems and species’ life histories (Nunney et al., 2015). Theoretically, species with longer lifespans or larger body size should be at a greater risk of cellular mutations that increase cancer risk due to a greater number of cellular divisions. However, genetic controls on neoplastic cellular proliferation vary between species, resulting in a lack of correlation between body size and cancer prevalence, a paradigm known as Peto's paradox (Caulin & Maley, 2011; Peto et al., 1975; Tollis et al., 2017). These genetic controls include the upregulation or duplication of TSGs and the downregulation of OGs within an organism. TSGs can control potentially carcinogenic mutations through various mechanisms, including apoptosis, cell cycle arrest, and senescence (Kumari et al., 2014). They can be divided into two major categories, caretakers and gatekeepers; caretaker genes control the maintenance of the genetic information integrity in each cell, whilst gatekeepers are genes that directly regulate tumour growth, codifying for proteins which either stimulate or inhibit proliferation, differentiation or apoptosis (Weitzman, 2001).

Gene duplication is considered an important mechanism for creating genetic novelty, as it has contributed to the evolution of developmental programmes, the plasticity of a genome, and the capability of a species to adapt to changing environments (Magadum et al., 2013; Ohno, 1970). It has been suggested that increased copy numbers of TSGs are amongst the most effective routes to enhanced cancer resistance (Vazquez & Lynch, 2021). Furthermore, duplicated TSGs can sometimes be selectively lost, which could be a macroevolutionary route towards lower cancer resistance (Glenfield & Innan, 2021). TSG duplication is one of the possible mechanisms behind increased cancer resistance in large‐bodied and/or long‐lived mammals. For example, low cancer mortality rates in elephants (Proboscidean lineage) may be linked to 20 genomic copies of the gene TP53 (Abegglen et al., 2015; Sulak et al., 2016), a tumour suppressor responsible for apoptosis, senescence, and cell cycle arrest in the presence of damaged DNA (Kumari et al., 2014). In blind mole rats (*Spalax* sp.), another tumour suppression mechanism has evolved, through duplications of genes in the interferon pathway, leading to interferon‐mediated concerted cell death, a strategy that has been proposed to counteract the weakened pro‐apoptotic function of the p53 protein (Gorbunova et al., 2012). A recent study in cetaceans indicated positive selection within the CXCR2 gene, an important regulator of DNA damage, tumour dissemination and immune system, and 71 duplicated genes, which had roles, such as the regulation of senescence, cell proliferation and metabolism (Tejada‐Martinez et al., 2021). Another recent study focusing on the evolution of elephants and their relatives (Proboscideans) from their smaller‐bodied ancestors (Afrotherians) indicated that tumour suppressor duplication was pervasive in Afrotherian genomes, suggesting that duplication of TSGs facilitated the evolution of increased body size (Sulak et al., 2016).

Another side of the TSG coin are OGs, genes that encode proteins that can induce cancer in animals (Croce, 2009; Lodish, 2000). Of the many known OGs, all but a few are derived from normal cellular genes called proto‐oncogenes, whose products participate in cellular growth‐controlling pathways (Lodish, 2000), by encoding proteins that stimulate cell division, inhibit cell differentiation, and halt cell death (Chial, 2008). All these processes are important for normal development and maintenance of tissues and organs. Due to their basic role in animal life, proto‐oncogenes have been highly conserved over eons of evolutionary time (Lodish, 2000). For growing big and/or living long, an increased number or function of proto‐oncogenes is expected, bringing along the risk of these genes turning into OGs by a gain‐of‐function mutation. This risk can be counteracted by an increase in the number of (copies of) TSGs. Whilst comparative studies have so far mainly focused on TSGs, a positive correlation between the number copies of TSGs and (proto‐)oncogenes is expected and has been demonstrated on the between‐species level in mammals (Tollis et al., 2020). We suggest that instead of focusing on the TSGs in comparative studies, a balance between TSGs and OGs should be considered, as it is possible that a species with a lower number of TSGs is still more resistant to cancer due to a lower number of OGs.

Using a wider variety of model species provides novel insights into the evolutionary and ecological importance of oncogenic processes (Baines, Lerebours, et al., 2021; Giraudeau et al., 2018; Hamede et al., 2020; Pesavento et al., 2018). Depending on their longevity, body size, life history strategy, as well as environmental (oncogenic) pressures, species should deploy different tumour suppression strategies. However, to date, comparative studies of tumour suppression mechanisms have focused on mammals (e.g. Abegglen et al., 2015; Seluanov et al., 2018; Tejada‐Martinez et al., 2021; Tollis et al., 2017; Vazquez & Lynch, 2021; Yu et al., 2021). This focus should be widened to include other vertebrate groups. Fish, and more specifically bony fish, are evolutionarily older and genetically more diverse than mammals (Buchmann, 2014), being a paraphyletic group whose last common ancestor is also an ancestor of the tetrapods and, therefore, all mammals. Accordingly, their tumour suppression mechanisms should not only include most of the mammalian mechanisms but also reveal novel (but potentially phylogenetically older) previously undetected mechanisms. There is evidence that fish lineages have evolved at increased rates of duplicated genes compared with mammals (Robinson‐Rechavi & Laudet, 2001), suggesting a possibility that tumour suppression and gene duplications could be related to life‐history more closely in fish compared to mammals. However, it has to be kept in mind that the taxonomy and genetics of fish are complicated compared to mammals. All teleost fish have gone through three rounds of whole‐genome duplication (WGD), and a fourth round of duplication has taken place in salmonids (the salmonid‐specific autotetraploidization event), which occurred in the common ancestor of salmonids ~100 Mya (Lien et al., 2016). Whilst only one autotetraploidization event has occurred in the common ancestor of salmonids, polyploidization has evolved independently on multiple occasions in Cyprinids, a large fish family, including species like common carp (*Cyprinus carpio*) and goldfish (*Carassius* sp.; Xu et al., 2019).

**FIGURE 1:**
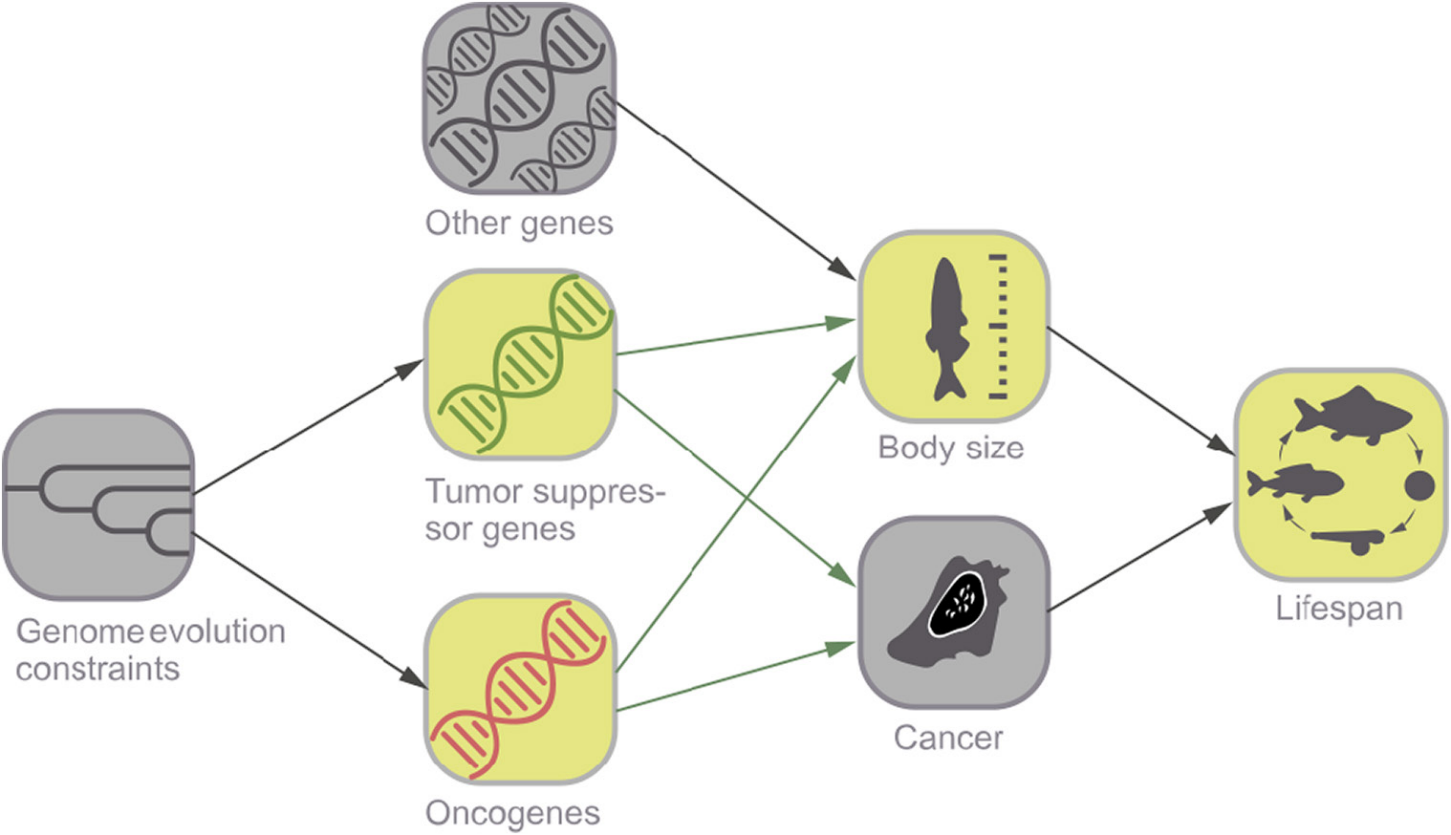
A directed acyclic graph depicting a simplified view of how lifespan may be influenced by copy numbers of TSGs and OGs. Gray boxes represent unobserved and green boxes represent observed variables. Arrows indicate possible causal paths. Green arrows indicate causal paths to be tested.

Tumor suppressor genes and oncogenes perform important tasks in retaining homeostasis. Arguably, the most notable role of these genes is to regulate growth. Indeed, any of the genes responsible for increased body size are also OGs, which increase cancer vulnerability in larger‐sized organisms together with the increased risks arising from greater number of cells and cellular divisions. Within species, body size is linked to cancer rate mostly through the number of cells (Nunney, 2018), while between species, the number of OGs is also affecting vulnerability to cancer. TSGs, however, have the role of reducing cell proliferation. Nevertheless, not all genes contributing to body size are TSGs or OGs. Furthermore, body size (larger individuals benefit from reduced predation rates) and cancer susceptibility are just two among many factors affecting animal lifespan. Based on these premises, we hypothesize that the lifespan of fish species is correlated positively to the copy numbers of TSGs and negatively to copy numbers of OGs when correcting for body size (Figure 1). To test this hypothesis, we have conducted a comparative analysis that examines the relationship between life history traits (longevity, body size) and the number of cancer‐related gene duplications in fish. Using the Catalogue of Somatic Mutations in Cancer (COSMIC; Sondka et al., 2018), we estimated the copy numbers of human cancer gene homologs in 85 complete genomes from across the phylogenetic tree of aquatic vertebrates (except mammals). We suggest that the COSMIC database, which is a collection of human cancer‐related genetic data, provides a reasonable proxy for testing our hypothesis on fish cancer genes for two main reasons: first, there is currently no wildlife version of this database, and second, there is an overlap between human gene homologs and those of other species (e.g. zebrafish [*Danio rerio*] have 70% overlap, Howe et al., 2013).

## 2 MATERIALS AND METHODS

### 2.1 Cancer genes

We estimated the copy numbers of OGs and TSGs of different fish species using publicly available phylogenetic trees for gene copy number counts (CAFE) and validated this efficient new approach against previous computational‐intensive alternatives. We used the manually curated human cancer genes COSMIC list, including tier 1 genes (those with strongly established link between mutations and cancer) and tiers 1 and 2 (tier 2 comprises genes with less extensive evidence linking them to cancer). Additionally, we noted whether genes were categorised as OG's or TSG's and somatic or germline mutations (Sondka et al., 2018). Additionally, we classified each TSG as being a gatekeeper gene or a caretaker gene according to the list provided by Tollis et al. (2020). For a detailed code, see Baines, Meitern, et al. (2021). The methods and R code for extraction of the data is described in Appendix S1 (Extraction of genes from the COSMIC list) and Appendix S2 (Get fish homolog gene counts for COSMIC genes).

We used the Ensembl CAFE and the Ensembl Biomart orthology database to calculate the number of gene copies across species. First, we downloaded the Ensembl CAFE (Herrero et al., 2016) species trees for all the COSMIC genes. This provided an estimation of gene gain and loss data for each species whilst also considering lineage information (De Bie et al., 2006; Herrero et al., 2016). Second, we downloaded the list of human COSMIC gene homologs for each species represented in the Ensembl database using BioMart (Kinsella et al., 2011) and counted the unique confident orthologs in each species for each gene (called “Homologue approach” in the Supporting Information). For both datasets, we accounted for potentially missing orthologs, from incomplete genome sequencing and assembly, by normalising the copy number counts. This was done by dividing the sum of all gene copies, for all genes, with the total number of orthologous genes found for that species (Tollis et al., 2020, see Appendix S3). Normalisation of copy counts was completed for both cancer gene lists (COSMIC Tier 1 and COSMIC Tier 1&2) and for both copy number count methods (CAFE and Homolog approach). Both the TSG and OG counts were implemented so that genes classified as both TSGs and OGs in the COSMIC database were excluded from the calculation of copy numbers.

### 2.2 Comparison and validation of our methods

Tollis et al. (2020) performed a comparative analysis on cancer gene copies across mammals using BLAST searches (for methods, see Tollis et al., 2020). In order to ensure our methods produced similar results to their study, we ran additional analysis on mammals for comparison and found results were similar to those in Tollis et al. (2020) (see Appendix S3 for full results of the methods comparison). Our method, in addition to being computationally less intensive, allows for simple re‐running of the analysis whenever the Ensembl databases are updated. However, it should be kept in mind that there are drawbacks to relying solely on the Ensemble database which has fewer species genomes available and is known to miss paralogs if genomes are not updated regularly (Sulak et al., 2016).

### 2.3 Trait data collection

The maximum length and lifespan data (as well as other parameters) were obtained mainly from FishBase (Froese & Pauly, 2021) and AnAge database (Tacutu et al., 2017). For some species for which lifespan and body size data was not available from either Fishbase or AnAge databases, we looked for reliable data in articles and other sources (detailed in Supporting Information: Table S31). Species with no maximum lifespan data were excluded from the dataset. The longevity quotient (LQ) was calculated according to Tollis et al. (2020). LQ gives an indication of how long a species' lifespan is compared with other species of similar size (LQ = observed longevity/expected longevity). For each species, expected longevity was calculated by fitting a linear regression to log_10_(maximum longevity) and log_10_(body mass).

### 2.4 Phylogenetic regression analysis (PGLS)

The phylogenetic tree for the fish species together with branch lengths was obtained from timetree.org (Kumar et al., 2017). Species that were missing in the timetree.org database were excluded from the analysis as phylogenetically informed regressions cannot be done without phylogenetic distances. The body size (maximum body length) and lifespan was log transformed prior to analysis. The normalized copy number counts were standardized (i.e. converted to *z*‐scores) prior to all analyses. However, the TSG/OG ratio was calculated by dividing the normalized TSG count with the normalized OG count. This ratio did not need standardization. All the statistical analysis was performed in R (version 4.0.5, R Core Team, 2021) using the caper package (Orme et al., 2013) for phylogenetically informed regressions. If *λ*, *κ* and *δ* values are provided, the branch lengths were optimized using maximum likelihood. *λ*, *κ* and *δ* values correspond to Pagel's branch‐length modifications (Pagel, 1997, 1999) as PGLS assumes that the characters evolve following a time‐scaled Brownian motion and that these branch‐length transformations allow for evolution that is not fully explained by the phylogeny (*λ*), changes in evolutionary rate across it (*δ*), or punctuated evolution (*κ*). Branch optimisation was undertaken to confirm that the results were not heavily dependent on default *λ*, *κ* and *δ* values and results presented below include both models, optimised and fixed (at 1), values for these. For more details on *λ*, *κ* and *δ*, see the caper package manual (Orme et al., 2013). Other used packages included base, utils, stats, (R Core Team, 2021) ggplot2 (Wickham et al., 2016), ggtree (Yu, 2020), tidytree (Yu, 2021), biomaRt (Durinck et al., 2009), ape (Paradis & Schliep, 2019), AnnotationDbi (Pagès et al., 2019), dagitty (Textor et al., 2016) and numerous dependencies within those.

### 2.5 Statistics

Phylogenetically adjusted regression was used to study the relationships between body size, ratio of copy numbers of tumor suppressor genes and oncogenes, and lifespan. To take into account the potential effect of multiple testing when the 34 tests conducted in the Supporting Information part S4 are added to the 5 tests conducted as part of the main article, we have applied Bonferroni correction to the results. For 39 tests, the Bonferroni significance threshold is 0.00128. All of the main results display *p* ≤ 0.0001, hence remain significant after Bonferroni correction up to 500 tests. We consider adding the tests in Supporting Information part S5 in the Bonferroni to be redundant, as these are the same tests that are performed in Appendix S4 (just smaller sample size, excluding salmonids and cyprinids). Similarly, we do not add the tests from Appendix S6 to Bonferroni correction, as the mammal comparisons performed for these supplementary analyses are just made for completeness/reference, not being a part of our hypotheses.

## 3 RESULTS

### 3.1 Lifespan vs. longevity

**FIGURE 2:**
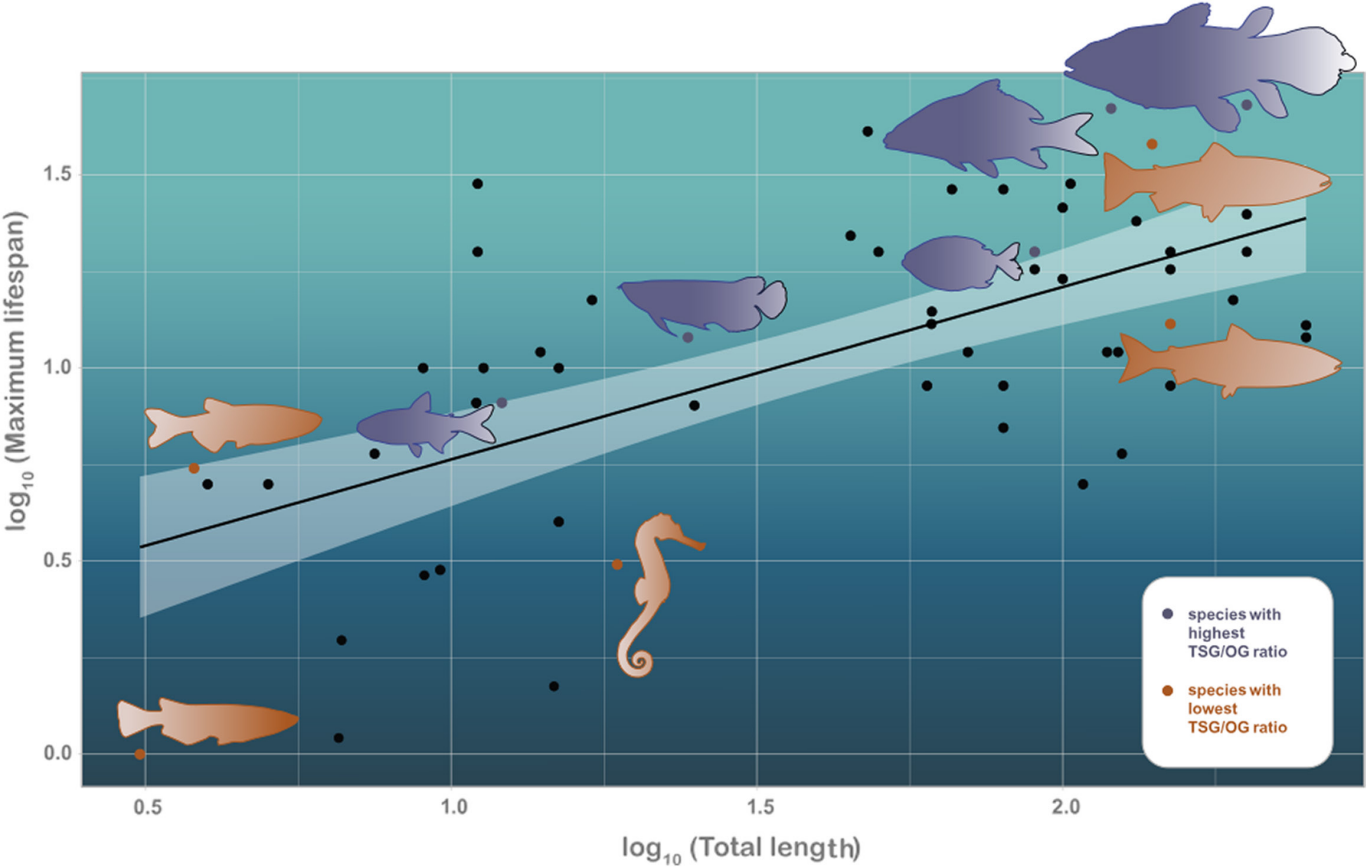
Linear regression between log transformed maximum body length and maximum lifespan. Each point in the plot represents a species. The line and the confidence intervals depicted in the plot come from standard linear regression, the values *R* ^2^, *p* and *N* are from phylogenetically adjusted regression, where *λ*, *κ* and *δ* are optimized using maximum likelihood. The images show the 5 species with the highest and lowest TSG/OG ratios. Highest ratio species (purple) are: *Latimeria chalumnae*, *Cyprinus carpio*, *Pygocentrus nattereri*, *Scleropages formosus*, *Astyanax mexicanus*. Lowest ratio species (orange) are: *Salmo salar*, *Danio rerio*, *Hippocampus comes*, *Salmo trutta*, *Oryzias sinensis*.

There was no maximum lifespan data available for 11 of the 65 fish species (*Actinopterygii*) that had genomes available. For the 54 species where this data was available, it was collected from AnAge for 23 species, 11 were taken from Fishbase and 10 from articles. The remaining data (10 species) were taken from 5 different, less reliable sources (Supporting Information: Table S31). For the 4 fishlike aquatic species (see Appendix S2 for clarification), 3 had age and size data available in Fishbase or AnAge. Nevertheless, from hereon, we will present results on the full dataset whilst the results using only fish species from class *Actinopterygii* are presented in Appendix S4 (Supporting Information: Figures S9–S20, Tables S1–S9). When branch lengths were optimized using maximum likelihood, maximum lifespan was related to maximum body size (Figure 2, *R* ^2^ = 0.34, *p* = 0.00001). At fixed branch lengths the relationship holds only for data from reliable sources (see Appendix S4: Figure S9, *p* = 0.1075 and Appendix S10 *p* = 0.0015). This is true for average age as well (Appendix S4: Figure S23, *p* = 0.0248).

### 3.2 Human cancer gene duplications in fish genomes

**FIGURE 3:**
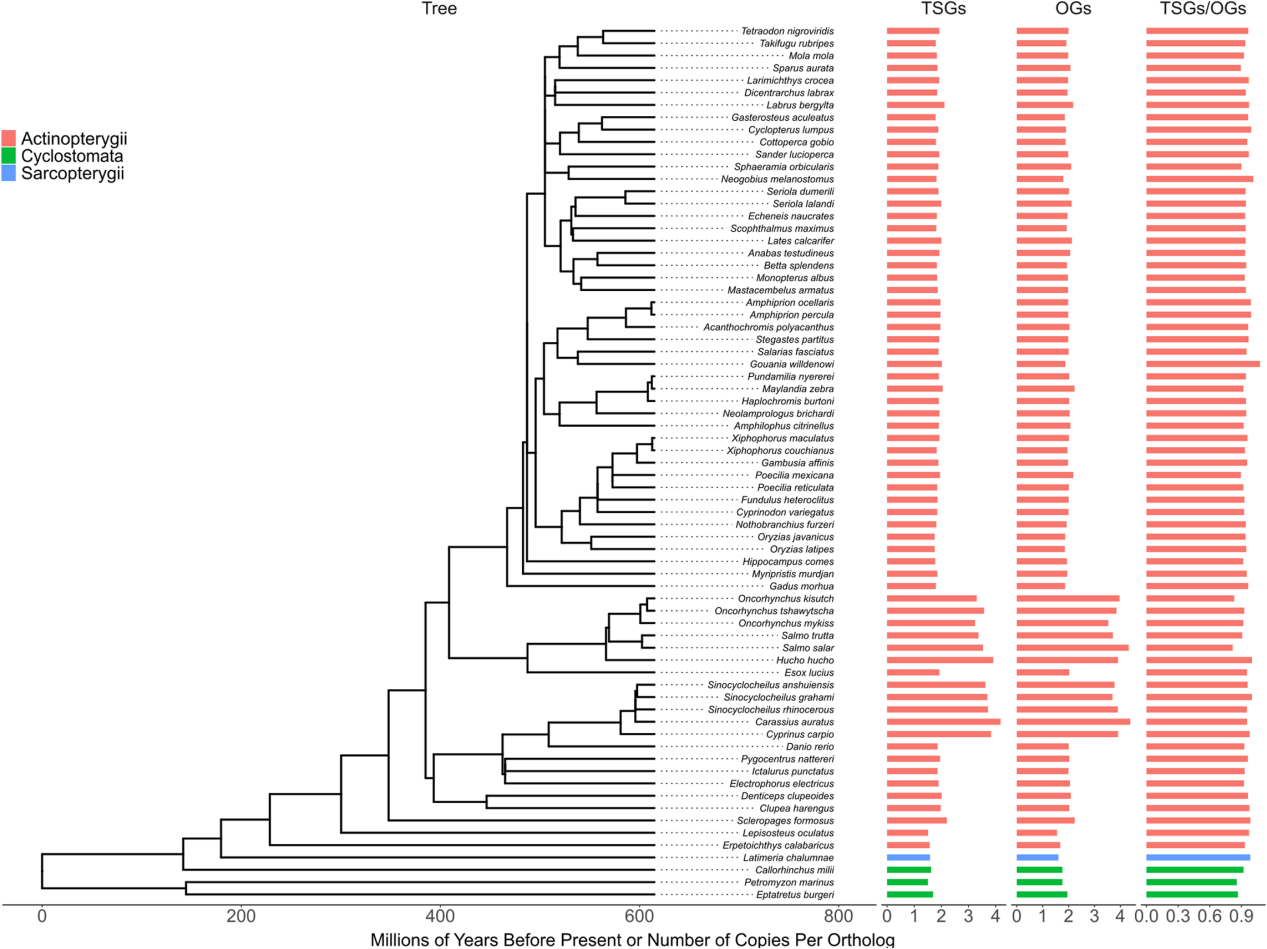
The species tree with branch lengths and counts for each species for normalized TSGs and OGs. TSGs/OGs is the ratio between normalized TSG counts and normalized OG counts.

We queried 68 genome assemblies representing three clades, Actinopterygii (ray‐finned fish, 65 species), Cyclostomata (jawless fish 3 species), and Sarcopterygii (fringe‐finned fish, 1 species; Figure 3) for 715 human cancer genes. Altogether the COSMIC list holds 243 pure TSGs, 243 pure OGs, 72 genes classified as both, 134 classified as pure fusion genes (i.e. genes resulting in cancer if translocated) and 31 genes as all (OGs, TSGs and fusions). We obtained normalized copy number counts for two cancer gene lists (COSMIC Tier 1 and COSMIC Tier 1&2, Tate et al., 2019), using copy number count methods that account for lineage information (CAFE, Herrero et al., 2016). As all species diverged from the lineage leading to humans at the same time point, we did not need to test for the potential systematic bias in our ability to identify human cancer genes in nonhuman genomes, as was done in the analogous comparative analysis of mammalian genomes (Tollis et al., 2020). From all queried human cancer genes, an average of 218 (±11 SD) TSG and 192 (±12 SD) OG orthologs were identified in these species using the CAFE approach and 170 (±31 SD) TSG and 152 (±27 SD) OG orthologs for the homolog approach (see the source data table in Baines, Meitern, et al., 2021 for numbers for all subsets). The methodology of obtaining copy number counts in this paper provides similar results to the methodology of Tollis et al. (see Appendix S3). In addition, the different COSMIC gene copy numbers (TSGs, OGs etc.) correlate positively (*R* > 0.3) regardless of the method used to obtain copy number counts (CAFE vs. Homolog) or subsets of cancer genes (COSMIC tier 1 vs. COSMIC tier 1&2, see Appendix S3). However, the total number of species in the analysis is larger for the CAFE approach, as the Homolog approach failed to produce copy number counts for some species.

### 3.3 Tumour suppressor genes balance oncogenes

**FIGURE 4:**
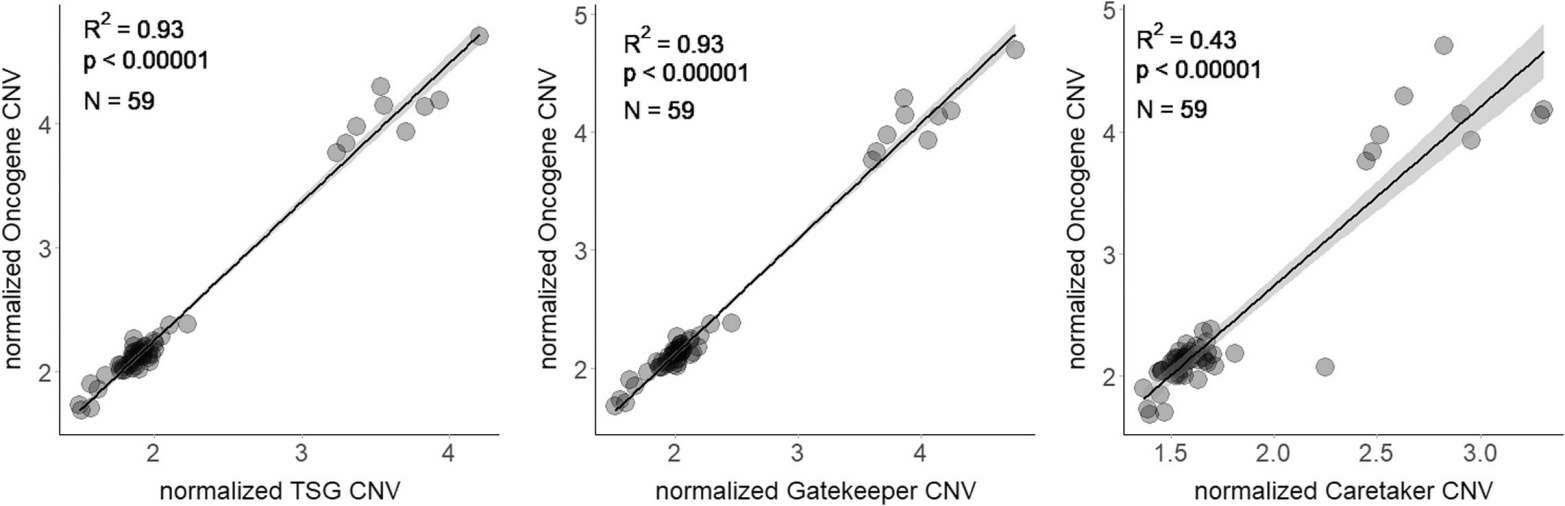
Linear regression between normalized copy numbers of different subsets of TSGs (all TSGs, gatekeeper genes and caretaker genes) and OG copy numbers. The copy numbers have been obtained using the CAFE approach and both COSMIC Tier 1 & 2 genes are included. Each point in the plot represents a species. The line and the confidence intervals depicted in the plot come from standard linear regression, the values *R* ^2^, *p* and *N* are from phylogenetically adjusted regression. The *λ*, *κ* and *δ* values are fixed at 1.

Using phylogenetically adjusted regressions, we found a strong positive correlation between the number of copies of OGs and TSGs (all TSGs: *R* ^2^ = 0.93, *p* < 0.00001, gatekeeper genes: *R* ^2^ = 0.93, *p* < 0.00001 and caretaker genes: *R* ^2^ = 0.43, *p* < 0.00001) in studied genomes (*N* = 59 for all comparisons, Figure 4). All these results (and most of the results described in the next section) remained significant when we removed the two fish families with an extra round of whole genome duplications (*Salmonidae* and *Cyprinidae*) from the analysis (see Appendix S5 file for analysis results without these two families, Figure S21–S32, Table S10–S18).

### 3.4 Tumour suppressor genes lengthen, oncogenes shorten lifespan

**FIGURE 5:**
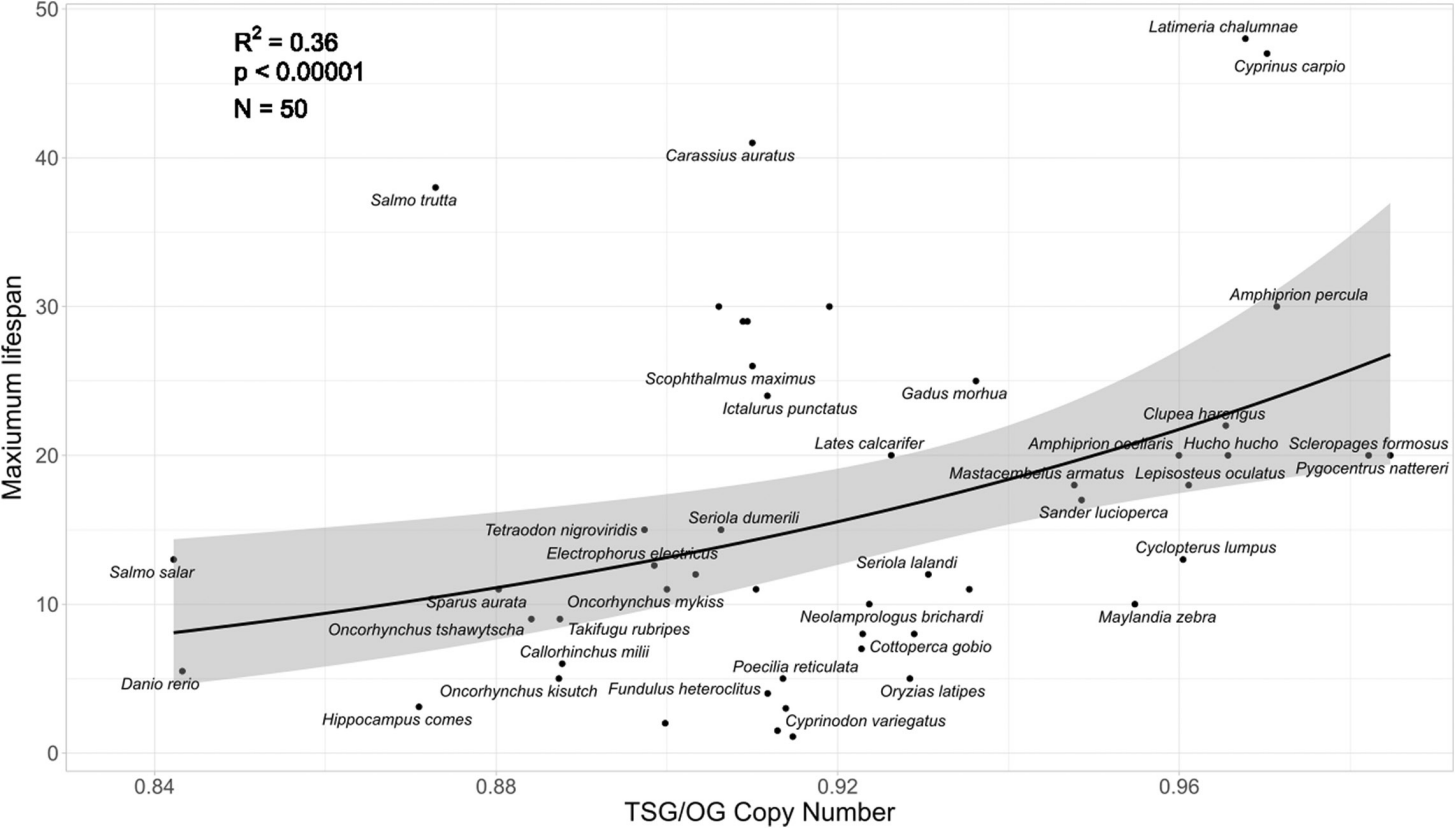
Linear regression between maximum lifespan and the normalized count of TSG, divided by the normalized count of OGs obtained from the CAFE approach and including only COSMIC Tier 1 genes. Each point in the plot represents a species in the dataset. The line and the confidence intervals depicted in the plot come from a log linked general linear model (i.e. not adjusted phylogenetically), the values *R* ^2^, *p* and *N* are from phylogenetically adjusted linear regression where the maximum lifespan is log transformed. The *λ*, *κ* and *δ* values are fixed at 1.

The magnitude of lifespan is positively related to the total number of TSGs and negatively to total number of OGs irrespective of branch length optimization (Table 1, Figure 5, for optimised branch lengths *p* < 0.00001 *R* ^2^ = 0.37, at fixed *p* < 0.00001 *R* ^2^ = 0.36), the inclusion or exclusion of body size (Appendix S4: Figure S19, Table S5), or low quality data points in the model (see Appendix S4). However, the relationship reveals itself only when both OG and TSG counts are included in the model or their ratio is used. The same result, a masking relationship between TSG and OGs, also holds true for another measure of lifespan: the longevity quotient (LQ; see Appendix S4: Figure S19). To test if the same masked relationship is present in the mammalian dataset, we ran a comparable analysis with mammals. In the mammalian dataset, we could not reveal the masked relationship between lifespan and TSGs or OGs (see Appendix S6: Figures S33–S42, Tables S19–S30).

**TABLE 1:** Phylogenetically adjusted regression with log body size and the normalized count of tumor supressor genes divided by the normalized count of oncogenes as predictors and log maximum lifespan as the response variable.

	Fixed *κ λ δ*				Maximum likelihood optimized *κ λ δ*			
	Estimate	SE	*t*	*p*	Estimate	SE	*t*	*p*
(Intercept)	−6.37	1.51	−4.23	0.0001	−3.33	1.08	−3.10	0.0033
Body size	0.19	0.14	137	0.177	0.37	0.07	5.00	<0.00001
TSG/OG	7.7	1.54	5.00	<0.00001	4.1	1.17	−3.51	0.001
	*κ* = 1	*λ* = 1	*δ* = 1		*κ* = 0.83	*λ* = 0	*δ* = 3	

*Note*: The predictors are body size (log maximum total length) and normalized TSG and OG copy number counts. The *λ*, *κ* and *δ* values are either fixed at 1 (left) or maximum likelihood optimized (right). The copy number counts have been obtained using the CAFE approach and only COSMIC Tier 1 genes are included. All species are included (*N* = 50). For 39 tests (5 in main analyses and 34 in supplementary analyses in Appendix S4), the Bonferroni significance threshold is 0.00128.

### 3.5 Species‐specific differences

We found that many human cancer genes are indeed also duplicated in fish genomes. The number of copies of genes varied between species, as did the ratio of TSGs/OGs (Figure 3). As expected, the species from the fish families that had undergone an extra round of whole genome duplication (*Salmonidae* and *Cyprinidae*) stand out as species with the highest copy numbers of TSGs and OGs. However, even within the fish species with smaller genomes, the number of copies of TSGs and OGs ranged from 1.5 to 2.2. When looking separately at fish species outside the salmonid and cyprinid families, species with highest copy numbers of TSGs are two tropical fish, Asian arowana (*Scleropages formosus*) and mormyrid electric fish (*Paramormyrops kingsleyae*), and one temperate fish, the ballan wrasse (*Labrus bergylta*; based on COSMIC tier 1 gene list, which is more reliable in regards of links of genes with cancer compared to the full list). As the number of copies of TSGs and OGs are correlated (*R* ^2^ = 0.93, *p* < 0.00001, Figure 4), we also calculated the TSG/OG ratio for all studied species (Figure 3), with the suggestion that species with the highest ratio invest more into cancer defences compared to species with the lowest ratio. Since this approach compensates for the whole genome duplication in two fish families, we can make comparisons across all studied species. According to this calculation, the three species with the highest TSG/OG copy number ratios were blind cave tetra (*Astyanax mexicanus*, TSG/OG copy number ratio 1.017), Asian arowana (0.985), and the red‐bellied piranha (*Pygocentrus nattereri*, 0.982). The three species with the lowest TSG/OG copy number ratio were zebrafish (0.843), Atlantic salmon (*Salmo salar*, 0.842), and reedfish (known also as ropefish, *Erpetoichthys calabaricus*, 0.837).

## 4 DISCUSSION

To date, comparative studies that have analysed cancer‐related gene duplications have been done on mammalian genomes (Tejada‐Martinez et al., 2021; Tollis et al., 2020; Vazquez & Lynch, 2021) and have suggested a link between lifespan and tumour suppressor gene copy numbers. Focusing on a phylogenetically older and genetically more diverse class of vertebrates could provide a control for the generalizability of the detected patterns but can also reveal patterns and trade‐offs that are not present in mammalian genomes. Here, we have provided the first comprehensive survey of cancer‐related gene duplications across the fish radiation, incorporating 715 human cancer genes with known orthologues in the genomes of 68 species.

Since proto‐oncogenes and TSGs are generally phylogenetically old, some of them dating back to the emergence of multicellularity (Lodish, 2000; Makashov et al., 2019), and in the absence of a more taxonomically wide database, it is reasonable to use human cancer gene database for studying cancer genes in fish. However, until it has been experimentally verified that human cancer genes in this study share the same function in other species, our results must be taken with caution. There are likely other fish‐specific pathways for tumour suppression in addition to those causally linked to human cancers, which we will miss in our current analysis. However, compared to previously published studies in mammals using the same human‐centred approach, our study benefits from the fact that the evolutionary distance from humans should not play a role in our comparative analysis on fish. We used the Ensembl Homology database and the Ensembl CAFE to calculate the gene copy numbers across species. Unfortunately, although the Ensembl database is considered of good quality, it is still missing a substantial number of species that already have a sequenced genome available. We cannot exclude the possibility that adding other aquatic vertebrate species to our dataset would have a significant effect on the results.

The results suggest the existence of a masked relationship between TSGs and OGs. When individually correlated with lifespan, the relationship between TSG and OG copy numbers and lifespan is not detectable, but when the ratio of TSG/OG is included in the model, there is a significant correlation with lifespan (Figure 5, *R* ^2^ = 0.36, *p* < 0.00001). This suggests that in order to achieve a longer lifespan, species must compensate for the number of cellular growth inducing proto‐oncogenes through increasing the number of copies of TSGs. In a previous comparative analysis with mammalian species, both the copy numbers of both TSGs and OGs were found to be positively correlated with longevity, a result that the authors found somewhat paradoxical (Tollis et al., 2020). Our results suggest that a high number of (proto‐)oncogene copies can indeed shorten lifespan and needs to be compensated for with a higher number of TSG copies. A positive correlation between TSG and OG copy number was found for both mammals (Tollis et al., 2020) and fish, supporting this conclusion. In the mammalian dataset, it is possible that a compensatory mechanism, causing this positive correlation between TSGs and OGs, hid the negative effect of OGs on lifespan. In our analysis using the same dataset as Tollis et al. (2020), we could also not reveal this masked relationship between lifespan and TSGs or OGs. As the Appendix S3 indicates, the CAFE and Homolog approaches computational methods provided somewhat different CNV estimates. It is possible that the Ensembl CAFE approach of calculating the gene gains and losses is also superior to the approach of Tollis et al. (2020) as it takes into account the phylogenetic tree of animals in copy number calculation. It is interesting to note that if we only kept mammal species with a genome assembly available in Ensembl (having a genome in Ensembl may be considered as having a genome of rather good quality (Kinsella et al., 2011)), we were able to indeed demonstrate the same masked relationship (TSGs lengthen and OGs shorten lifespan if both are in the model together) in mammals that we discovered in the fish dataset. One possible explanation why the masked relationship does not hold as strongly for mammals compared to fish is the relatively small phylogenetic distance between different mammal species, compared to the distance differences within fish species. It might be that such a relationship emerges only on a larger phylogenetic scale. Another possible explanation is that the cancer genes that have an ortholog in fish are the most conserved and/or more important in terms of lifespan. Accordingly, we could speculate that the masked relationship only reveals itself in the fish and not in the mammal dataset, as other less relevant and perhaps evolutionary more novel, cancer‐related genes are included.

Similarly to mammals and birds (where a strong correlation exists between lifespan and body mass, Healy et al., 2014), fish that live longer generally have longer bodies. As in other vertebrate classes, some species of fish live longer than expected for their body size, and some live shorter lives compared to other species in similar size. TSGs and OGs might be part of the story behind this variation, keeping in mind that it is mostly ecological selection pressures that have shaped lifespans of species over evolutionary time (Healy et al., 2014). We did not find a strong relationship between body size and TSG or OG copy numbers in our study. Species that grow larger tend to have slightly more copies of TSGs and OGs (Figure S30 in Appendix S5), but this trend is weak, and in the case of OGs, non‐significant. Indeed, there are other adaptive roles for proto‐OGs in addition to growth, e.g., cellular maintenance and survival (Creek et al., 2018). Whilst the positive association between TSG copy numbers and body length is expected, it was also not found in the similar comparative analysis of mammals (Tollis et al., 2020). Although it is known that within species, cancer risk increases with body size (Nunney, 2018), this relationship does not seem to hold on the between‐species level, potentially because of the upregulation of cancer defence mechanisms (Caulin & Maley, 2011). We do not yet have good cancer prevalence data for most fish species, so it is still early to make conclusions about the so‐called Peto's paradox (no increase in cancer prevalence with increased body size, Peto et al., 1975) in fish, but our results suggest that it is certainly a promising future research direction. Indeed, as fish grow throughout their life, it is logical to assume that defence mechanisms against the cost of growth (e.g. potentially increased cancer risk) are even more pronounced in this vertebrate class compared to classes with finite growth.

Species that have most cancer gene duplications in our study are the species that have gone through more rounds of whole genome duplications. When we exclude salmonids and cyprinids, the species with the highest number of TSG copies are two tropical species, Asian arowana and mormyrid electric fish, and one temperate fish, the ballan wrasse. All these species stand out, as they have been selected among the few fish species for which the genome has been sequenced. For example, the genome of the mormyrid was sequenced in order to understand the evolution and development of electric organs, and to identify candidate housekeeping genes related to electrogenesis (Gallant et al., 2017). We could speculate that these are the fish with strongest tumour suppression systems, similarly to elephants (*Loxodonta Africana*), naked mole rats (*Heterocephalus glaber)*, two‐toed sloth (*Choloepus hoffmanni*) and nine‐banded armadillos (*Dasypus novemcinctus*) in mammalian class (Tollis et al., 2020).

However, as we can see that TSG and OG copy numbers are correlated (Figure 4, *R* ^2^ = 0.93, *p* < 0.00001), we suggest that looking at TSG copy numbers in relation to OG copy numbers might be more informative in terms of cancer resistance and cancer susceptibility. This approach also allows the inclusion of salmonids and cyprinids in the discussion. Blind cave tetra is the species with the highest TSG/OG ratio in our dataset. This species has undergone a recent rapid evolutionary change, dividing into two subspecies, one that lives in total and permanent darkness and lacks eyes and pigmentation, the other an “ancestral” multi‐coloured tropical freshwater fish. With cave colonization, this species has undergone strong selective pressure and extreme morphological evolution and can be used to understand the evolution of specific traits and genetic mechanisms that support rapid habitat‐based evolutionary change (Torres‐Paz et al., 2018). Whether stronger tumour suppression is one of these traits remains to be studied. Next in line is the Asian arowana, who is still among the top three, in absolute TSG copy numbers as well as TSG/OG copy number ratio. This endangered and highly valued ornamental species stands out among fish due to its late sexual maturation and unusually high level of parental care (Scott & Fuller, 1976). High tumour resistance could therefore be considered as a trait related to slow life history (Boddy et al., 2020). The last of the three species with highest TSG/OG ratio is red‐bellied piranha, another fish species for whom parental care has been described (Queiroz et al., 2010).

Three species with the lowest TSG/OG copy number ratio were zebrafish, Atlantic salmon, and reedfish. Zebrafish has become one of the most common model organisms in cancer research in recent decades, due to rapid development, ease of care, similarity of tumourigenesis to humans, and its well‐studied genome (Stoletov & Klemke, 2008). If the fast life‐history of zebrafish is linked to higher cancer susceptibility, zebrafish might be a model organism that is more similar to mice than to humans in terms of the evolution of tumour suppression mechanisms. In addition to the Atlantic salmon, several other salmon species tend to have low TSG/OG ratio. We could speculate that this is also related to life history, as several salmon species are semelparous, breeding only once in their life. Reproduction in semelparous species can lead to rapid severe pathology known as reproductive death by various mechanisms, due to very high levels of reproductive effort and drastically lowered investment in self‐maintenance (Gems et al., 2021). Reduced tumour suppression could be one part of this strategy of low self‐maintenance investment and prioritisation of growth/reproduction. The species with the lowest TSG/OG ratio in our dataset was reedfish, a facultative air‐breather with an elongated body and the ability to move in both aquatic and terrestrial environments (Sacca & Burggren, 1982). It might be assumed that adaptation to two very different environments would also require strong tumour suppression mechanisms, but that does not appear to be the case for reedfish. Based on this finding, we could speculate that switching between terrestrial and aquatic environments, and various levels of oxygen, could be an environmental factor that suppresses oncogenic processes, rather than induces them. Indeed, in humans, it has been shown that a change in oxygen pressure (hyperbaric oxygenation) could inhibit tumour cell proliferation (Granowitz et al., 2005). Whether reedfish are indeed better protected against cancer due to changes in oxygen pressures, therefore being able to afford lower investment in genome‐based tumour suppression mechanisms, remains to be studied.

Whilst this field of research is still in its infancy ‐ the number of fish species that have been sequenced is still small, and the link between gene copy numbers and cancer is based on human data – it already shows great promise in providing a better understanding of the evolution of tumour suppression mechanisms. From the life‐history perspective, we can suggest that fish species with slow life‐history might exhibit stronger genomic defences against oncogenic processes, whereas fish with semelparous mating systems could be less protected against cancer.

This finding might have applications in conservation, as it might be possible to predict which species could be more vulnerable to oncogenic environmental change (e.g. oncogenic pollution exposure, Baines, Meitern, et al., 2021). Moreover, environmental change has shown to increase the risk of virus‐induced cancer. Currently, approximately 15% of cancers in humans are associated with oncogenic viruses; however, a viral aetiology of cancer was initially described in animals (Pesavento et al., 2018). Widening the research into the evolution of tumour suppression mechanisms to incorporate other taxa could provide novel insights into tumour suppression that might be able to be applied to human cancer research. Additionally, it is well known that fishing can induce the evolution of traits such as size and age at maturity in the target species, but also induce larger, ecosystem level changes, some of which may be indirect (Czorlich et al., 2022). Increased mortality from fishing is expected to favour faster life histories, realized through earlier maturation, increased reproductive investment, and reduced post‐maturation growth (Heino & Dieckmann, 2015). Therefore, it is possible that human induced gene‐pool modification might have an effect of cancer prevalence as a result of trade‐offs in, for example, reproductive investment, at the expense of tumour suppression as life‐span/body size is reduced from overfishing.

In conclusion, we were able to demonstrate a masked relationship between the number of copies of cancer related genes and maximum lifespan in fish species and can suggest that a higher TSG count is probably behind the increased lifespan in some species. This masked relationship only reveals itself in fish data, similar comparative analysis in mammals did not support this finding (Appendix S6), which indicates that studying different wild animal groups could provide complementary information about the evolution of tumour suppression. As fish are evolutionarily older and more diverse group compared to mammals, it is intriguing to suggest that fish studies could be a yet largely unexplored treasure trove for understanding the evolution and ecology of cancer. This field of research is a two‐way street: it could provide novel insights into the study of cancer and tumour suppression, and also the study of fish evolution, life‐histories, and ecology.

## CONFLICT OF INTEREST

We declare no conflict of interest.

## Supporting information


Appendix S1–S7.

## Data Availability

Data and code for this study are available at the Zenodo data repository: https://doi.org/10.5281/zenodo.5791154 (Baines, Meitern, et al., 2021).
