# Supplementary material for: Comparative study of the evolution of cancer gene duplications across fish
Source: Evol Appl. 2022 Sep 29;15(11):1834–45. doi: 10.1111/eva.13481 (PMC9679246; doi:10.1111/eva.13481)
Supplement: Supplementary file 1 — Appendix S1–S7. [file EVA-15-1834-s001.pdf]

Supplementary materials

S1. Extraction of genes from the COSMIC list

S2. Get fish homolog gene counts for COSMIC genes

S3. Comparison of the method from Tollis et al. (2020) and the Ensembl CAFE and Ensembl Homologue approach from this paper using mammalian data

S4. The plots from the main article, including only ray-finned fishes

S5. The plots from the main article, including only teleost fish which have gone through three rounds of whole-genome duplication (ray-finned fishes (Class: Actinopterygii) without salmonids and cyprinids.)

S6. Copy number regressions for mammal cancer genes, to check if the masked relationship detected in this paper fish datasets holds in the mammal dataset.

S7. Sources for age data

S1. Extraction of Genes from the COSMIC List

The aim of this supplementary material is to pre-proccess genes from the COSMIC (version 92) database (Sondka et al. 2018) to add fields for distinguishing tumour suppressor genes, oncogenes, somatic and germline genes for firstly, only tier 1 (576 genes) and secondly, tier 1 & 2 genes (723 genes). In addition, gatekeeper and caretaker genes are extracted using the list of Tollis et al. (2020). The generated table will then serve as a basis for further copy number variation analysis.

Data

```
dir <- "./data/"

#setup COSMIC column names
cols1 <- c("Gene.Symbol",
          "Name",
          "Synonyms",
          "ensemblID",
          "conservedAtTaxonomicLevel")

cols2 <- c(
          "Tier",
          "Hallmark",
          "Somatic",
          "Germline",
          "Tumour.Types.Somatic.",
          "Tumour.Types.Germline.",
          "Cancer.Syndrome",
          "Tissue.Type",
          "Molecular.Genetics",
          "Role.in.Cancer",
          "Mutation.Types",
          "Translocation.Partner",
          "Other.Germline.Mut",
          "Other.Syndrome")
cols <- c(cols1, cols2)
```

The input data ".csv" file is downloaded from *COSMIC (version 92)* database (Sondka et al. 2018).

```
cosmic <- read.csv(paste0(dir, "CensusCOSMICv92.csv"))


#cleanup data
cosmic$x <- sapply(cosmic$Role.in.Cancer, strsplit, ",")
cosmic$x <- sapply(cosmic$x, function(x) sapply(x, trimws))

cosmic$TSG <- sapply(cosmic$x, function(x) ifelse("TSG" %in% x, T, F))
cosmic$oncogene <- sapply(cosmic$x, function(x) ifelse("oncogene" %in%
```

```
x, T, F))
cosmic$fusion <- sapply(cosmic$x, function(x) ifelse("fusion" %in% x,
T, F))

cosmic$x <- sapply(cosmic$Synonyms, strsplit, ",")
cosmic$x <- sapply(cosmic$x, function(x) sapply(x, trimws))

#get ensembl gene ID's
cosmic$ensemblID <- sapply(cosmic$x, function(x)
unlist(x[grep("ENSG*", x, F, F)]))
cosmic$ensemblID<- as.character(cosmic$ensemblID)
cosmic$ensemblID<- sapply(cosmic$ensemblID, function(x){
  strsplit(x, ".", fixed = T)[[1]][1]
  })

#find missing ensembl IDs
missingNames <-cosmic$Gene.Symbol[cosmic$ensemblID == "NULL"]
#missingNames
missingEntrez <- cosmic$Entrez.GeneId[cosmic$ensemblID == "NULL"]
#missingEntrez
```

Used AnnotionDbi to find ENSEMBL gene identifiers (eID) for those that do not have it in COSMIC.

```
#list colnames in AnnotationDbi
AnnotationDbi::columns(org.Hs.eg.db::org.Hs.eg.db)
```

Assign first by symbol.

```
#library("org.Hs.eg.db") # remember to install it if you don't have it
already

symbols <- AnnotationDbi::mapIds(org.Hs.eg.db::org.Hs.eg.db,
                                 keys = as.character(missingNames),
                                 keytype = "SYMBOL", column="ENSEMBL")

#symbols

cosmic$ensemblID[cosmic$ensemblID == "NULL"] <-
  symbols[cosmic$Gene.Symbol[cosmic$ensemblID == "NULL"]]
```

Then by alias.

```
missingNames <-cosmic$Gene.Symbol[is.na(cosmic$ensemblID)]
#missingNames

#library("org.Hs.eg.db") # remember to install it if you don't have it
already
symbols <- AnnotationDbi::mapIds(org.Hs.eg.db::org.Hs.eg.db,
```

```
                                         keys = as.character(missingNames),
                                         keytype = "ALIAS", column="ENSEMBL")

#symbols

cosmic$ensemblID[is.na(cosmic$ensemblID)] <-
  symbols[cosmic$Gene.Symbol[is.na(cosmic$ensemblID)]]

#remove the ensembl ID from the synonym field
cosmic$Synonyms <-  sapply(cosmic$x, function(x) {
  out<- c(1, grep("ENSG*", x, F, F))
  x<-x[setdiff(1:length(x), out)]
  paste0(x, collapse = ", ")
  })

cosmic$ensembl_gene_id <- cosmic$ensemblID
```

Add gatekeepers and caretakers to TSGs list.

The file “msaa125_supplementary_data/GK_CT Genes.xlsx” is from the supplementary materials of Tollis et al. (2020).

```
gtctFname <- "data/msaa125_supplementary_data/GK_CT Genes.xlsx"
gkGenes <- readxl::read_excel(gtctFname,
    sheet = "GK", col_types = c("text"))
ctGenes <- readxl::read_excel(gtctFname,
    sheet = "CT", col_types = c("text"))
gkGenes <- gkGenes$`Gatekeeper Genes`
ctGenes <- ctGenes$`Caretaker Genes`

cosmic$gateKeeper <- ifelse(cosmic$Gene.Symbol %in% gkGenes, T, F)
cosmic$careTaker <- ifelse(cosmic$Gene.Symbol %in% ctGenes, T, F)

cosmic$Somatic <- ifelse(tolower(cosmic$Somatic) == "yes", T, F)
cosmic$Germline <- ifelse(tolower(cosmic$Germline) == "yes", T, F)

#cleanup
cosmic$SynonymsList <- cosmic$x
cosmic$x <- NULL
```

Write results

```
source("./functions/dataIO.R", encoding = "UTF-8")

writeResults <- TRUE
cosmicTSG <- cosmic[cosmic$TSG & cosmic$Tier == 1,]
cosmicOnco <- cosmic[cosmic$oncogene & cosmic$Tier == 1,]
```

```
cosmicPureTSG <- cosmicTSG[!cosmicTSG$oncogene & !cosmicTSG$fusion,]
cosmicPureOnco <- cosmicOnco[!cosmicOnco$TSG & !cosmicOnco$fusion,]


#write data to R data files
#the directory (dir) must exist
dir <- ("./data/proccessed/cosmic/tier1/")

if(writeResults){
  writeRDS(cosmicTSG, dir)
  writeRDS(cosmicOnco, dir)
  writeRDS(cosmicPureTSG, dir)
  writeRDS(cosmic, "./data/proccessed/cosmic")

}
```

References

Tollis M, Schneider-Utaka AK, Maley CC. (2020). The Evolution of Human Cancer Gene Duplications across Mammals. Molecular Biology and Evolution 37, 2875–2886.

Sondka Z, Bamford S, Cole CG, Ward SA, Dunham I, and Forbes SA. (2018). The COSMIC Cancer Gene Census: describing genetic dysfunction across all human cancers. Nature Reviews Cancer 18, 696.

S2. Get Fish Homolog Gene Counts for COSMIC Genes

Introduction

The aim of this of document is to get copy number count of fish genes for all of the human cancer associated genes. The cancer genes are from the *COSMIC (version 92)* database (see S1). The fish are those that have a sequenced genome in the Ensembl (v104) database and have a CAFE tree associated with them. The copy number counts are obtained by two methods:
1. Downloading the Ensembl CAFE gene duplication species trees for all of the COSMIC genes and extracting the copy numbers from there.
2. Downloading the list of human COSMIC gene orthologs for each fish species represented in the Ensembl database using BioMart and counting the unique confident orthologs in each species for each COSMIC gene.

Functions

The script below uses several custom functions to perform the aforementioned tasks.

```
source("./functions/dataIO.R", encoding = "UTF-8")
source("./functions/parseTreeData.R", encoding = "UTF-8")
source("./functions/parseDF.R", encoding = "UTF-8")
source("./functions/ensembl.R", encoding = "UTF-8")
```

Precompiled data

```
#check if directories exist
dirs <- c("./data",
          "./data/proccessed",
          "./data/proccessed/cosmic/",
          "./data/ensembl")

if(!all(dir.exists(dirs))){
  stop(paste0("directories missing: \n",
              paste0(dirs[!dir.exists(dirs)], collapse = "\n")))
       }

# read the preproccesed cosmic data prepeared in S1
cosmic <- readRDS("./data/proccessed/cosmic/cosmic.rds")
```

Altogether the COSMIC list holds 243 pure TSGs, 243 pure oncogenes, 72 genes classified as both, 134 classifid as pure fusion and 31 genes as all (oncogenes, TSGs and fusions). If we subset for **Tier 1** the COSMIC list holds 202 pure TSGs, 212 pure oncogenes, 69 genes classified as both, 93 classified as pure fusion and 30 genes as all.

1. Getting CAFE gene trees

Downloading and pre-proccesing the gene trees from the Ensembl CAFE database.

```
#should the latest data be downloaded far all of the enembl IDs in
COSMIC
#at first run change it to TRUE
redownload <- FALSE

if(redownload){
  genes <- cosmic$ensemblID
  for(gene in genes){
    downloadGenetree(gene)
  }
}

treesAll <- loadGeneTrees(cosmic$ensemblID)

## Warning in loadGeneTrees(cosmic$ensemblID): 5 genes absent in
datadir:
##  ENSG00000280757, ENSG00000274267, ENSG00000282600,
ENSG00000251562, ENSG00000197880
```

These 5 genes are not present in the CAFE gene tree of Ensembl.

Getting species

As the gene "ENSG00000148584" has a tree that has the most ray-finned fish species we use this for pruning the trees.

```
geneID <- "ENSG00000148584"
fish<-getNodeChildrenNames(treesAll[[geneID]], "Actinopterygii")
```

Next we also added fishlike species not from the class *Actinopterygii* (3 species of jawless fish and 1 species of fringe-finned fish) to the list of species to enable checking whether this will affect the final results. These species are:

```
## add non fish aquatic vertebrates
tetrapods <- getNodeChildrenNames(treesAll[[geneID]], "Tetrapoda")
sarcopterygi <- getNodeChildrenNames(treesAll[[geneID]],
"Sarcopterygii")
cyclostomata <-getNodeChildrenNames(treesAll[[geneID]],
"Cyclostomata")
Gnathostomata <- getNodeChildrenNames(treesAll[[geneID]],
"Gnathostomata")
Euteleostomi <-  getNodeChildrenNames(treesAll[[geneID]],
"Euteleostomi")
moreFishlike <- setdiff(Gnathostomata, Euteleostomi)
fishlike <- c(setdiff(sarcopterygi, tetrapods), cyclostomata,
moreFishlike)

fish <- c(fish, fishlike)
```

These species are: *Latimeria_chalumnae, Eptatretus_burgeri, Petromyzon_marinus, Callorhinchus_milii*

```
ensemblFish <- sapply(names(fish), function(x){
  res<-strsplit(tolower(x), " ")[[1]]
  paste0(substring(res[1],1,1),res[2])
})
names(ensemblFish) <- fish
```

Load trees for all studied species

Next we are pruning the trees to keep only the selected species and then extracting the gene counts from these lists.

```
#because it is a long command we'll save it
prunedTreesFile <- "./data/fishTree.rds"
#should the file be over written by new data
overWrite <- FALSE

if(!file.exists(prunedTreesFile) | overWrite){
  treesFull <- pruneTrees(treesAll, fish)
  saveRDS(treesFull, prunedTreesFile)
}
treesFull <-readRDS(prunedTreesFile)

#calcualting the copy numbers for COSMIC genes in the pruned trees
geneDupliLong<-getGeneCounts(treesFull)
```

2. Get ortholog gene counts from ensembl

The number of human homologs for each gene in the list of selected species is downloaded from Ensembl.

```
#set  downloadHumanHomologues to TRUE to download data
#It might fail if Ensembl is not responisve
downloadHumanHomologues<-FALSE

#NB the directory must exist for saving!
humanHomologuesFile <- "./data/ensembl/fishCosmicHumanHomologues.rds"

if(downloadHumanHomologues){
  sp="hsapiens"
  version=104
  ensembl<- biomaRt::useEnsembl(biomart = "genes", version=version)
    ensembl <- biomaRt::useDataset(dataset=paste0(sp,
"_gene_ensembl"),
                                   mart = ensembl)
    homologPages <- biomaRt::listAttributes(ensembl, page =
"homologs")
    homologPages$species <- sapply(homologPages$name, function(x){
      strsplit(x, "_", fixed = T)[[1]][1]
    })
    sps<-unique(homologPages$species)
    existingSpecies <- intersect(ensemblFish, sps)
    humanHomologues <- sapply(existingSpecies, getEnsemblHomologues,
                              ids = cosmic$ensemblID,
                              sp = "hsapiens",
                              ensembl = ensembl,
                              homologPages = homologPages)

    saveRDS(humanHomologues, humanHomologuesFile)

}
if(file.exists(humanHomologuesFile)){
  humanHomologues <- readRDS(humanHomologuesFile)
}
```

Now we have collected the duplication data for all available COSMIC genes for all available fish also using the ortholog technique.

Combine the copy number counts of the 2 methods (CAFE and ortholog).

```
#merge the data frames by species names
ensemblFishInv<-names(ensemblFish)
names(ensemblFishInv)<-ensemblFish
humanHomologuesConfidence <- Map(cbind, humanHomologues,
```

```
                              ensembl_sp = names(humanHomologues),
                              sp = ensemblFishInv[names(humanHomologues)])

reduceColNames<-function(d){
  names(d) <- gsub(paste0(unique(d$ensembl_sp),"_homolog_"),"",
names(d))
  d
}
humanHomologuesConfidence <- Map(reduceColNames,
humanHomologuesConfidence)


reduceDF<-function(d, f=sum){
  aggregate(orthology_confidence ~ ensembl_gene_id + sp , data=d, FUN
= f)
}
humanHomologuesConfidence <- Map(reduceDF, humanHomologuesConfidence)

humanHomologuesConfidence <-
unique(do.call(rbind,humanHomologuesConfidence))


geneDupliLong<-merge(geneDupliLong, humanHomologuesConfidence,
         by = c("ensembl_gene_id","sp"), all.x = T)



geneDupliLong<-merge(geneDupliLong, cosmic,
                    by = "ensembl_gene_id",
                    all = T)
```

For all 70 species there is 715 unique genes identified. The COSMIC gene list has 723 genes. For 3 of the later the Ensembl gene identifier was not found, so altogether 720 genes were queried using the ortholog and CAFE approach.

Generate summary table for regression analysis

```
#CNV variables of intrest
geneTypes <-  c("TSG","oncogene", "gateKeeper", "careTaker")
```

Next we will combine all of the data into a single data frame so that we have for each TSG, oncogene, gateKeeper, careTaker a COSMIC CAFE and ortholog approach gene counts. This is for both COSMIC Tier 1 and COSMIC Tier 1&2 genes. In addition this aggregation is done for somatic and germline cancer genes similar to Tollis et al. (2020).

```
d <- geneDupliLong[geneDupliLong$isSpecies, ]
d <- d[!is.na(d$ensembl_gene_id),]
d$nodeGeneCount <- as.numeric(d$nodeGeneCount)
```

```r
#aggregate the different subsets so that each species has 1 row
#later join by the species name
cat("\nOrthology genes\n")
```

```
##
## Orthology genes
```

```r
dct1 <- joinAggregateSubset(d, confidentOnly = T,
                            aggCol = "orthology_confidence",
                            normOnly = T, tiers = 1)
```

```
## TSG has on average 137 (25 SD), genes for COSMIC tier 1.
## Oncogene has on average 132 (24 SD), genes for COSMIC tier 1.
## TSG&Oncogene has on average 44 (8 SD), genes for COSMIC tier 1.
## GateKeeper has on average 142 (26 SD), genes for COSMIC tier 1.
## CareTaker has on average 36 (7 SD), genes for COSMIC tier 1.
## Somatic has on average 298 (55 SD), genes for COSMIC tier 1.
## Germline has on average 25 (5 SD), genes for COSMIC tier 1.
## Somatic&Germline has on average 48 (8 SD), genes for COSMIC tier 1.
```

```r
cat("\nCAFE genes\n")
```

```
##
## CAFE genes
```

```r
dgt1 <- joinAggregateSubset(d, confidentOnly = F,
                            aggCol = "nodeGeneCount",
                            normOnly = T, tiers = 1)
```

```
## TSG has on average 180 (9 SD), genes for COSMIC tier 1.
## Oncogene has on average 165 (10 SD), genes for COSMIC tier 1.
## TSG&Oncogene has on average 59 (4 SD), genes for COSMIC tier 1.
## GateKeeper has on average 179 (9 SD), genes for COSMIC tier 1.
## CareTaker has on average 53 (4 SD), genes for COSMIC tier 1.
## Somatic has on average 386 (22 SD), genes for COSMIC tier 1.
## Germline has on average 36 (2 SD), genes for COSMIC tier 1.
## Somatic&Germline has on average 58 (3 SD), genes for COSMIC tier 1.
```

```r
cat("\nOrthology genes\n")
```

```
##
## Orthology genes
```

```r
dcta <- joinAggregateSubset(d, confidentOnly = T,
                            aggCol = "orthology_confidence",
                            normOnly = T)
```

```
## TSG has on average 170 (31 SD), genes for COSMIC tiers 1 and 2.
## Oncogene has on average 152 (27 SD), genes for COSMIC tiers 1 and
2.
## TSG&Oncogene has on average 44 (9 SD), genes for COSMIC tiers 1 and
2.
## GateKeeper has on average 164 (30 SD), genes for COSMIC tiers 1 and
2.
## CareTaker has on average 45 (8 SD), genes for COSMIC tiers 1 and 2.
## Somatic has on average 384 (70 SD), genes for COSMIC tiers 1 and 2.
## Germline has on average 27 (5 SD), genes for COSMIC tiers 1 and 2.
## Somatic&Germline has on average 49 (8 SD), genes for COSMIC tiers 1
and 2.
```

```
cat("\nCAFE genes\n")
```

```
##
## CAFE genes
```

```
dgta <- joinAggregateSubset(d, confidentOnly = F,
                            aggCol = "nodeGeneCount",
                            normOnly = T)
```

```
## TSG has on average 218 (11 SD), genes for COSMIC tiers 1 and 2.
## Oncogene has on average 192 (12 SD), genes for COSMIC tiers 1 and
2.
## TSG&Oncogene has on average 61 (4 SD), genes for COSMIC tiers 1 and
2.
## GateKeeper has on average 207 (11 SD), genes for COSMIC tiers 1 and
2.
## CareTaker has on average 64 (4 SD), genes for COSMIC tiers 1 and 2.
## Somatic has on average 498 (29 SD), genes for COSMIC tiers 1 and 2.
## Germline has on average 39 (3 SD), genes for COSMIC tiers 1 and 2.
## Somatic&Germline has on average 60 (3 SD), genes for COSMIC tiers 1
and 2.
```

```
cat("\nCAFE genes\n")
```

```
##
## CAFE genes
```

```
items <- lapply(geneTypes,
  function(x, d){
    cat(paste0(x, " only \n"))
    joinAggregateSubset(d[d[[x]],], confidentOnly = F,
                                  aggCol = "genePresent",
                                 normOnly = T, tiers = 1,
                    subsets = c("Somatic","Germline",
```

```
                                                      "Somatic&Germline"))
  }, d)

## TSG only
## Somatic has on average 160 (9 SD), genes for COSMIC tier 1.
## Germline has on average 34 (2 SD), genes for COSMIC tier 1.
## Somatic&Germline has on average 45 (2 SD), genes for COSMIC tier 1.
## oncogene only
## Somatic has on average 199 (12 SD), genes for COSMIC tier 1.
## Germline has on average 6 (0 SD), genes for COSMIC tier 1.
## Somatic&Germline has on average 20 (1 SD), genes for COSMIC tier 1.
## gateKeeper only
## Somatic has on average 134 (8 SD), genes for COSMIC tier 1.
## Germline has on average 14 (1 SD), genes for COSMIC tier 1.
## Somatic&Germline has on average 32 (1 SD), genes for COSMIC tier 1.
## careTaker only
## Somatic has on average 20 (2 SD), genes for COSMIC tier 1.
## Germline has on average 21 (2 SD), genes for COSMIC tier 1.
## Somatic&Germline has on average 12 (1 SD), genes for COSMIC tier 1.

# rename columns
items <- Map(function(df, name, cols=NULL){
  if(is.null(cols)){
    cols = c(2:ncol(df))
  }
  colnames(df)[cols] <- paste0(colnames(df)[cols], name,
"EnsemblCafeTier1")
  df

}, df=items, name=geneTypes)
mergeList<-function(x, y, ...){
  merge(x, y, by = "sp")
}

mutTypeCNV <- Reduce(mergeList, items)


cancerGeneCNV <- merge(dgta, dcta, by = "sp",
                suffixes = c("EnsemblCafe", "EnsemblHomolog"),
                all = T)

cancerGeneCNVt1 <- merge(dgt1, dct1, by = "sp",
                suffixes = c("EnsemblCafe", "EnsemblHomolog"),
                all = T)

cancerGeneCNV <- merge(cancerGeneCNV, cancerGeneCNVt1, by = "sp",
                suffixes = c("TierAll", "Tier1"),
```

```r
                  all = T)

cancerGeneCNV <- merge(cancerGeneCNV, mutTypeCNV, by = "sp",  all = T)

#NB the directory must exist!
cnvFile <- "./data/proccessed/FishCOSMICwideCNVnorm.rds"
saveRDS(cancerGeneCNV, cnvFile)
```

Almost identical approach as above is used for getting the copy number counts of mammal cancer related genes analyzed in supplementary materials S5 and S6. The main difference is in pruning the tree with Mammalia instead of Actinopterygii.

S3. Tollis Mammal and Ensembl CAFE Mammal Comparsion

Introduction

The aim of this supplementum is to establish if the methods for obtaining counts of cancer gene copy numbers produce similar results. The methods to compare are the method from Tollis et al. (2020) and the Ensembl CAFE and Ensembl ortholog approach from this paper. The species in the comparison include those mammal species that are present in all of the datasets.

Read data

Read in the original data from Tollis et al. (2020) and the COSMIC data from Baines et al. 2021b. Also read the copy number data for Ensembl CAFE Herrero et al. 2016 and ortholog approach calculated as in Baines et al. (2021b).
There are 33 species that are present in all data frames.

Check calculations

Lets first check if our method of obtaining normalized gene counts is the same as used in Tollis et al. (2020). The y-axes represents the data from this paper while the x-axes are from the Tollis et al. (2020) paper.
## TSG has on average 227 (14 SD), genes for COSMIC tiers 1 and 2.
## Oncogene has on average 222 (12 SD), genes for COSMIC tiers 1 and 2.
## TSG&Oncogene has on average 65 (5 SD), genes for COSMIC tiers 1 and 2.
## GateKeeper has on average 225 (14 SD), genes for COSMIC tiers 1 and 2.
## CareTaker has on average 66 (5 SD), genes for COSMIC tiers 1 and 2.
## Somatic has on average 415 (25 SD), genes for COSMIC tiers 1 and 2.
## Germline has on average 37 (3 SD), genes for COSMIC tiers 1 and 2.
## Somatic&Germline has on average 62 (4 SD), genes for COSMIC tiers 1 and 2.

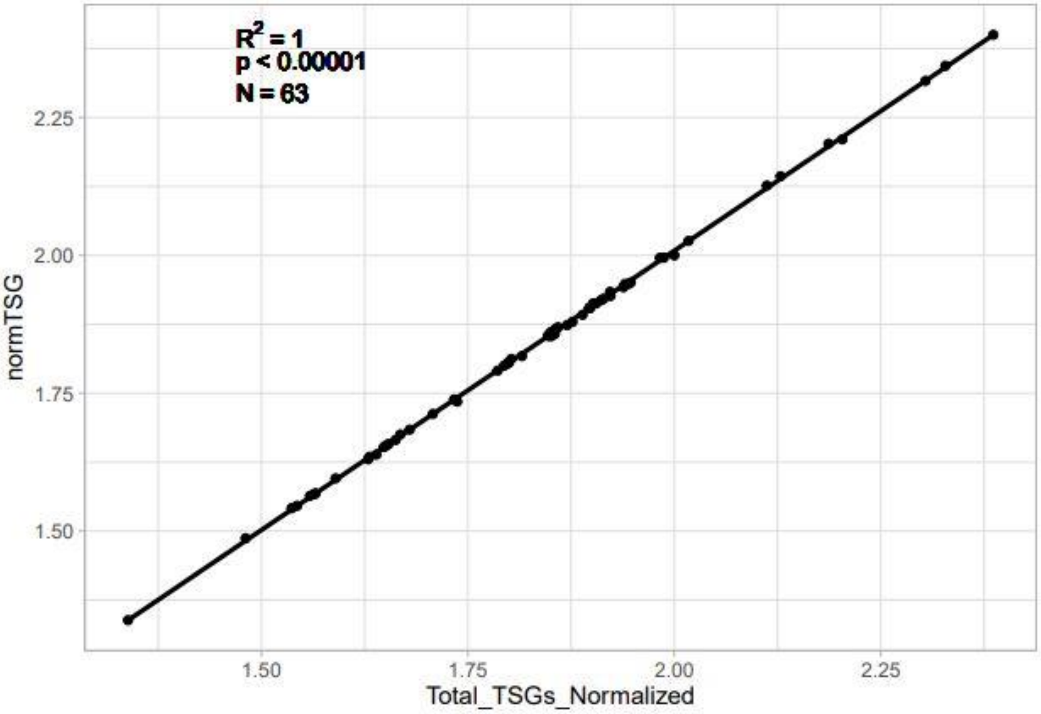

Figure S1. Linear regression between TSG copy number calculations. The y-axes represent the data calculated in this paper from Tollis' original data whilst the x-axes are from the paper of Tollis et al. (2020).

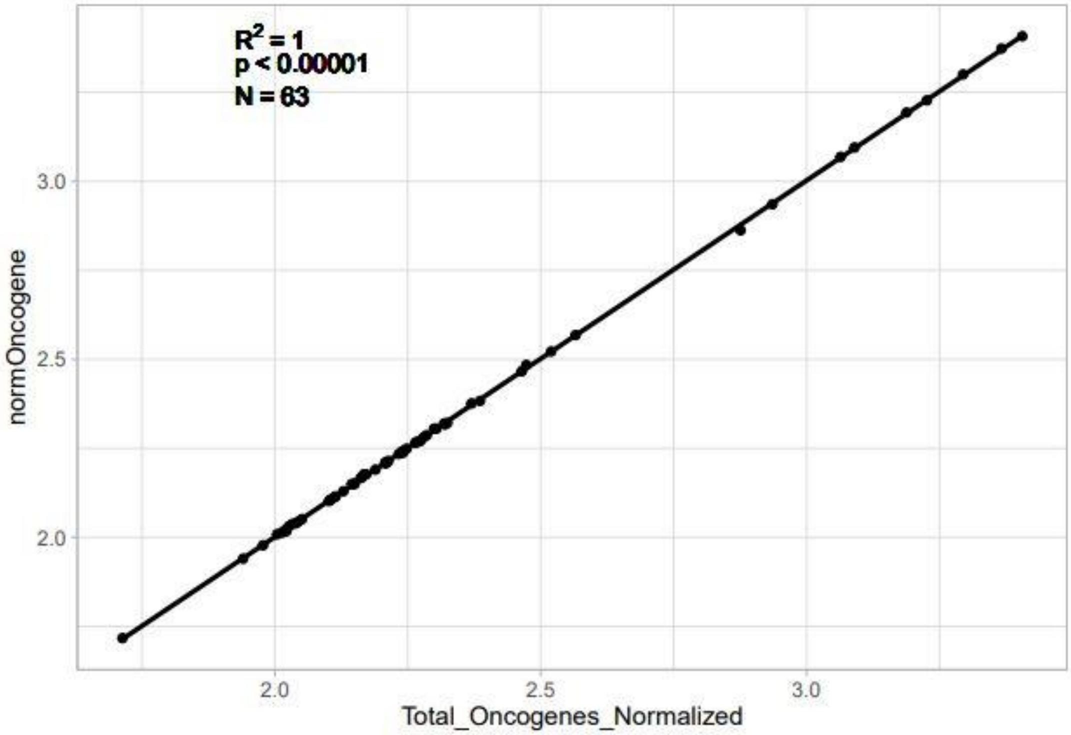

Figure S2. Linear regression between oncogene copy numbers calculations. The y-axes represent the data calculated in this paper from Tollis' original data whilst the x-axes are from the paper of Tollis et al. (2020).

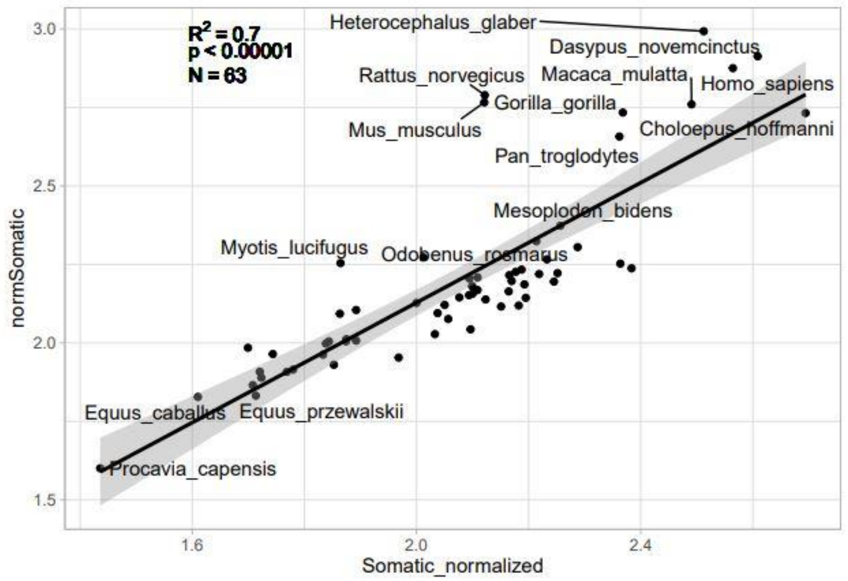

Figure S3. Linear regression between Somatic gene copy numbers calculations. The y-axes represents the data calculated in this paper from Tollis' original data whilst the x-axes are from the paper of Tollis et al. (2020).

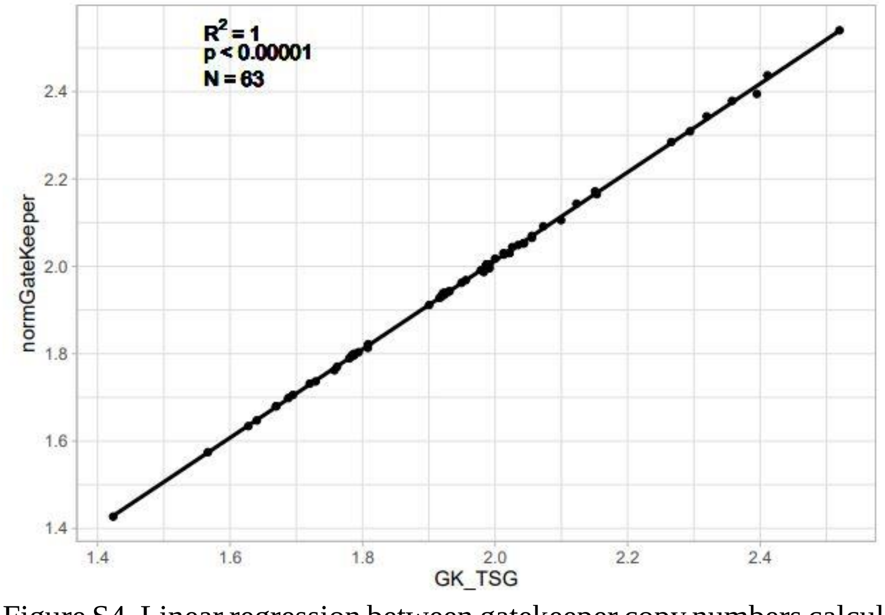

Figure S4. Linear regression between gatekeeper copy numbers calculations. The y-axes represents the data calculated in this paper from Tollis' original data whilst the x-axes are from the paper of Tollis et al. (2020).

The above plots confirm that (at least for most reported values in Tollis et al. 2020) the normalisation is performed consistently and repeatably. The difference in Somatic may come from changes in the COSMIC database or because Somatic only are those that have no Germline mutation. This can be checked but is largely irrelevant.

Correlations
TSGs

Lets see if different measures from Tollis et al. (2020) for TSG count yield similar results to our Ensembl CAFE and homologue approach.

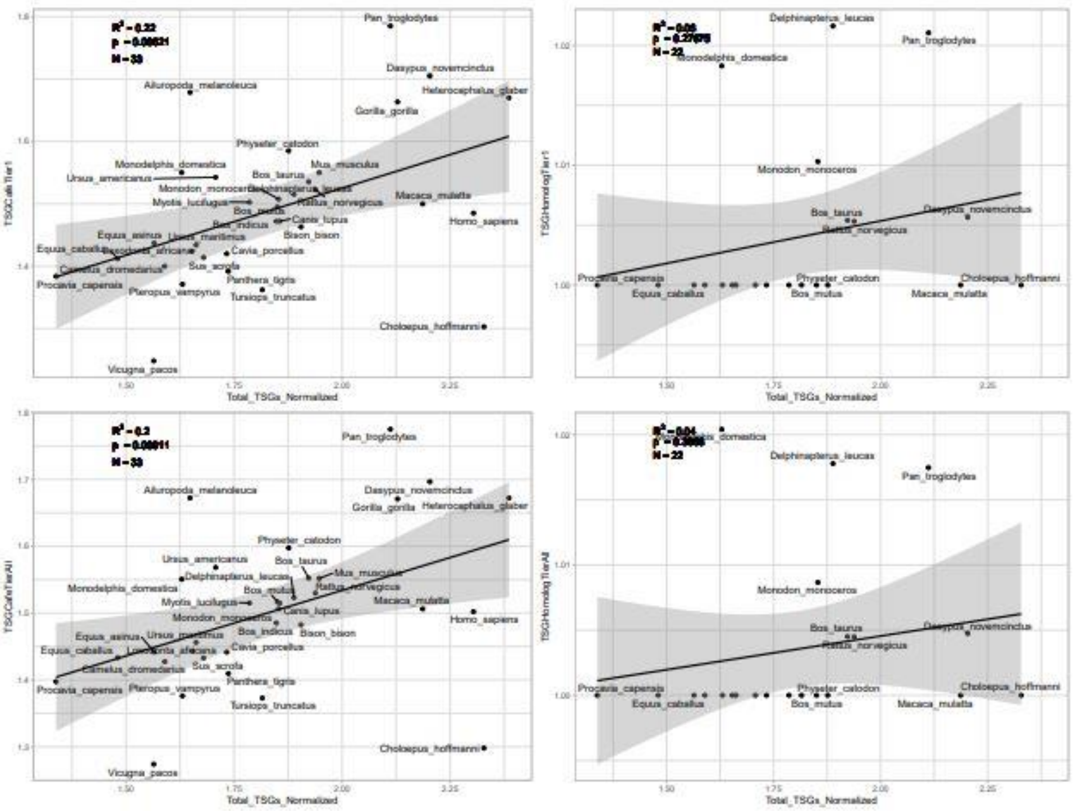

Figure S5. Linear regression between TSG copy number calculations. The y-axes represent the data from this paper extracted from Ensembl CAFE or the Homolog approach whilst the x-axes are from the paper of Tollis et al. (2020). Rows are different COSMIC Tiers (tier 1 or Tier 1&2) and columns represent Ensembl CAFE or Homolog approach. The data on x axis is same for all plots.

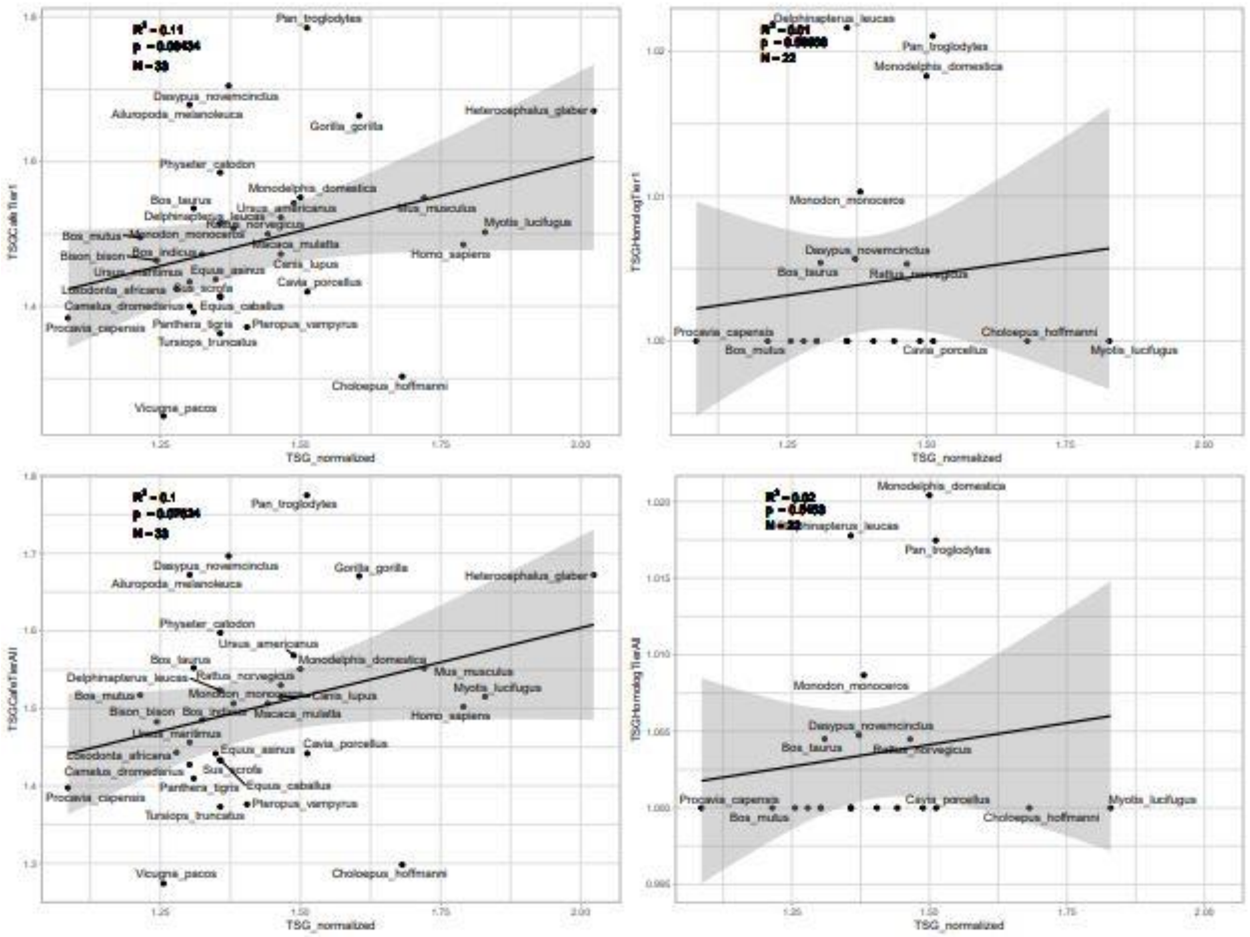

Figure S6. Linear regression between TSG copy number calculations. The y-axes represent the data from this paper extracted from Ensembl CAFE or the Homolog approach whilst the x-axes are from the paper of Tollis et al. (2020). Rows are different COSMIC Tiers (tier 1 or Tier 1&2) and columns represent Ensembl CAFE or Homolog approach. The data on the x axis is the same for all plots.

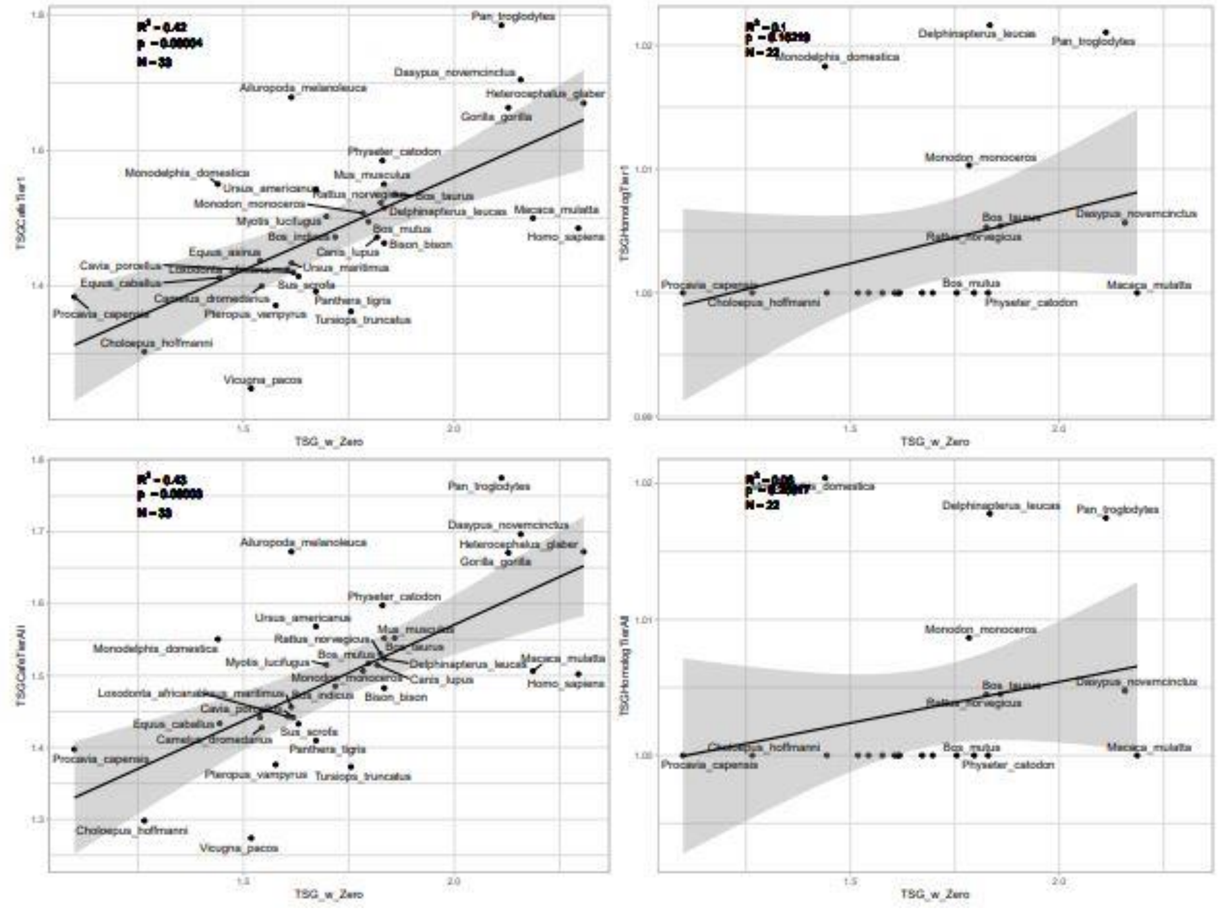

Figure S7. Linear regression between TSG copy number calculations. The y-axes represents the data from this paper extracted from Ensembl CAFE or the Homolog approach whilst the x-axes are from the paper of Tollis et al. (2020). Rows are different COSMIC Tiers (tier 1 or Tier 1&2) and columns represent Ensembl CAFE or Homolog approach. The data on the x axis is the same for all plots.

From the above plots it is evident that there is a significant positive correlation between the Tollis “TSG_w_Zero” and the data extracted from Ensembl CAFE but it is not very strong. Also, the mammal homolog calculations gives a completely different result due to the differences in Ensembl Homolog level calculations beween taxa. So the homolog approach should not be used for mammalian data.

Oncogenes

Lets see if different measures from Tollis et al. (2020) for oncogene counts yields similar results as the Ensembl CAFE and the homolog approach. The Oncogene results are similar to the TSG results.

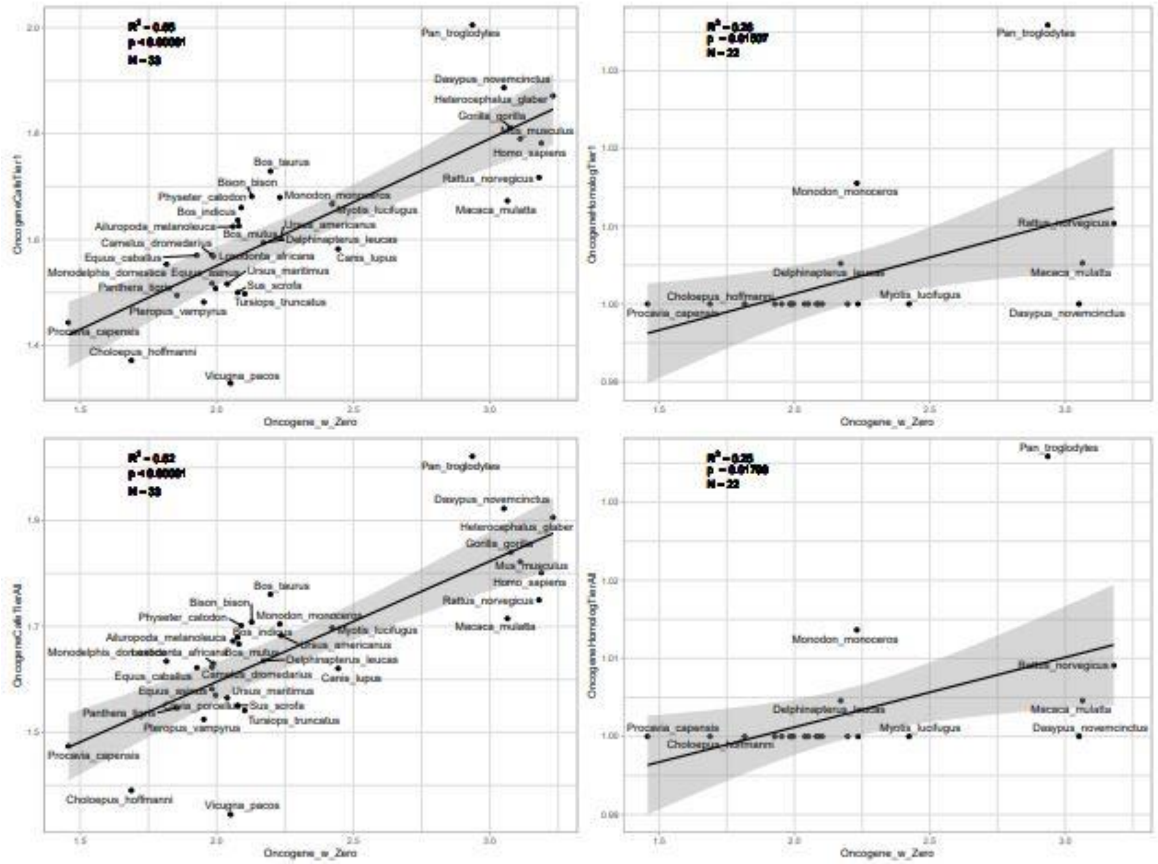

Figure S7. Linear regression between Oncogene copy number calculations. The y-axes represents the data from this paper extracted from Ensembl CAFE or the Homolog approach whilst the x-axes are from the paper of Tollis et al. (2020). Rows are different COSMIC Tiers (tier 1 or Tier 1&2) and columns represent Ensembl CAFE or Homolog approach. The data on x axis is same for all plots.

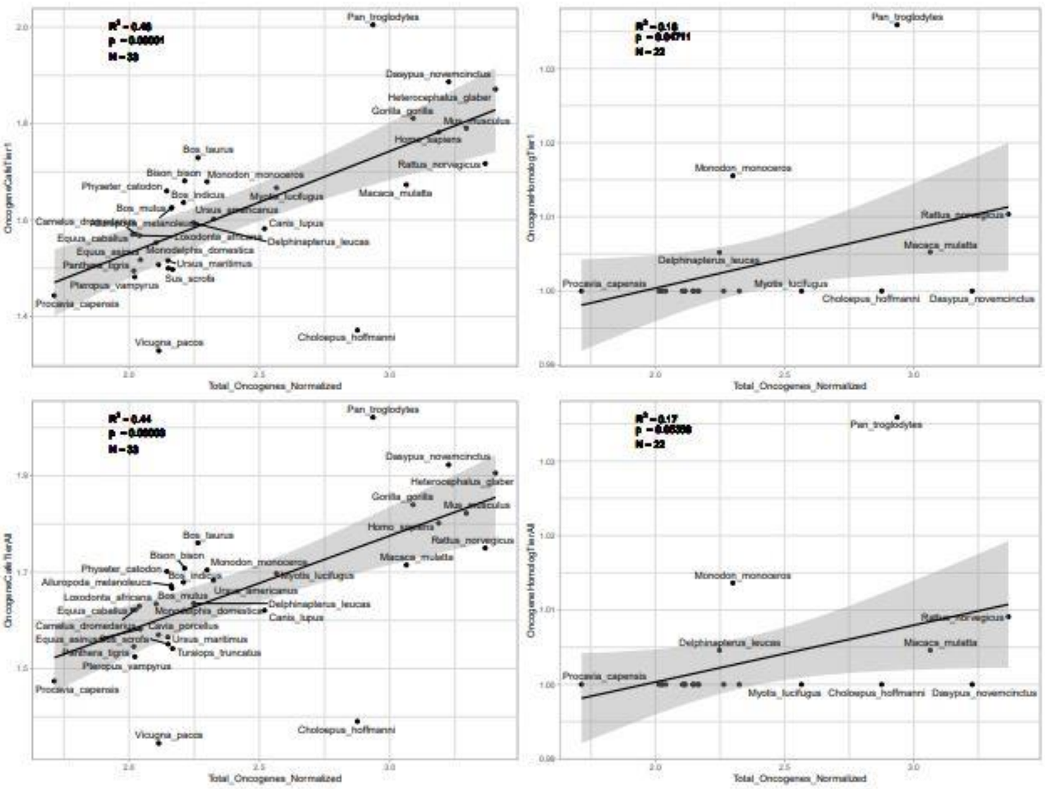

Figure S8. Linear regression between Oncogene copy number calculations. The y-axes represents the data from this paper extracted from Ensembl CAFE or the Homolog approach whilst the x-axes are from the paper of Tollis et al. (2020). Rows are different COSMIC Tiers (tier 1 or Tier 1&2) and columns represent Ensembl CAFE or Homolog approach. The data on x axis is the same for all plots.

Conclusions

The measures correlate but the correlations are not strong. Strangely the with 0 approach of Tollis et. al (2020) correlates best with the Ensembl CAFE results of this paper. So we can say the datasets are reasonably different but aim to measure the same thing.

References

Herrero J, Muffato M, Beal K, Fitzgerald S, Gordon L, Pignatelli M, Vilella AJ, Searle SMJ, Amode R, Brent S, et al. 2016. Ensembl comparative genomics resources. Database 2016, no. bav096 (January 1, 2016). https://doi.org/10.1093/database/bav096.

Tollis M, Schneider-Utaka AK, Maley CC. 2020. The Evolution of Human Cancer Gene Duplications across Mammals. Molecular Biology and Evolution 37, 2875–2886.

S4. Copy Number Regression Plots of Fish Cancer Genes

Introduction

This supplementum aims to plot additional graphs and tables for referencing in the main text. These plots and tables are meant to clarify and illustrate the data and analyses and differ from the ones in the main article mainly by including only ray-finned fishes (Class: *Actinopterygii*).

Lifespan vs size

First let us plot to see if the magnitude of maximum lifespan is related to the magnitude of maximum body length in all fish species. The underling hypothesis is that species who live longer also tend to be larger.

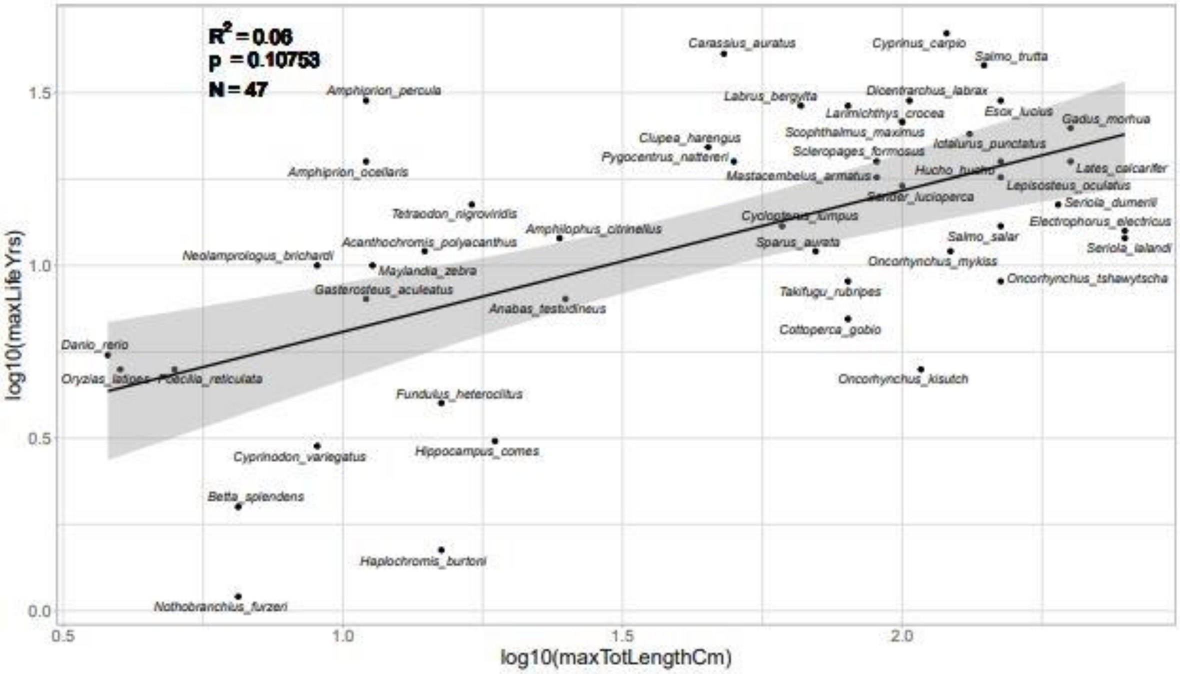

Figure S9. Linear regression between log transformed maximum body length and maximum lifespan. Each point in the plot represents a species. The line and the confidence intervals depicted in the plot come from standard linear regression, the values R2, p and N are from phylogenetically adjusted regression.

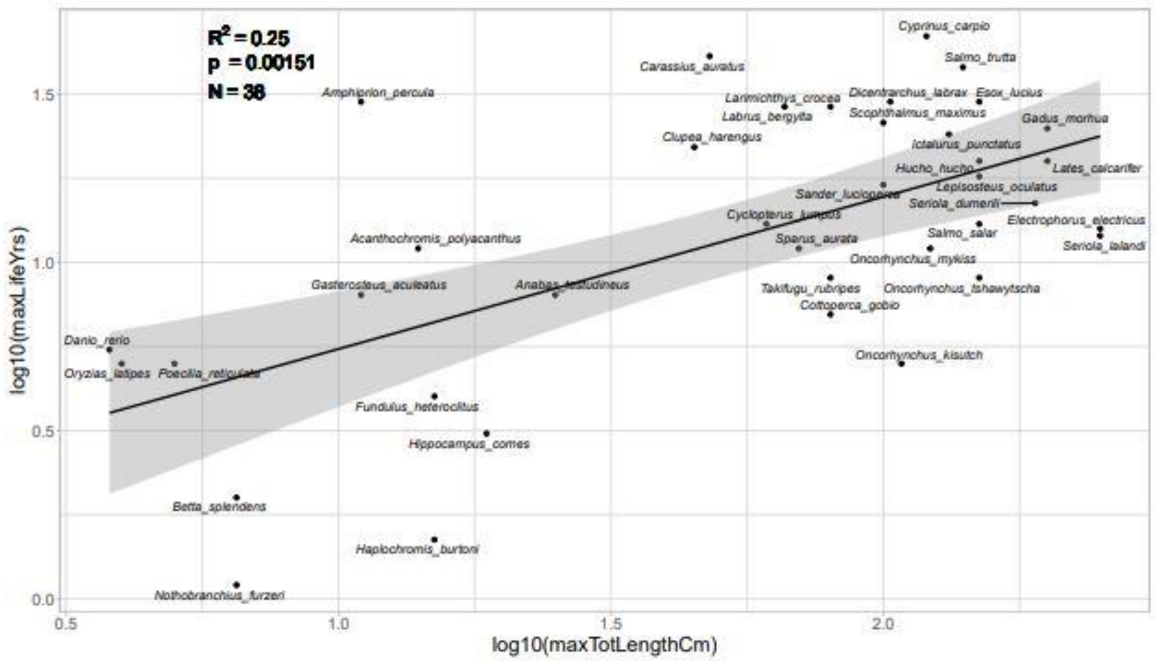

Figure S10. Linear regression between log transformed maximum body length and maximum lifespan. Each point in the plot represents a species. Only species included are those for whom the lifespan and body size data is extracted from data from AnAge database, fishbase or articles. The line and the confidence intervals depicted in the plot come from standard linear regression, the values R2, p and N are from phylogenetically adjusted regression.

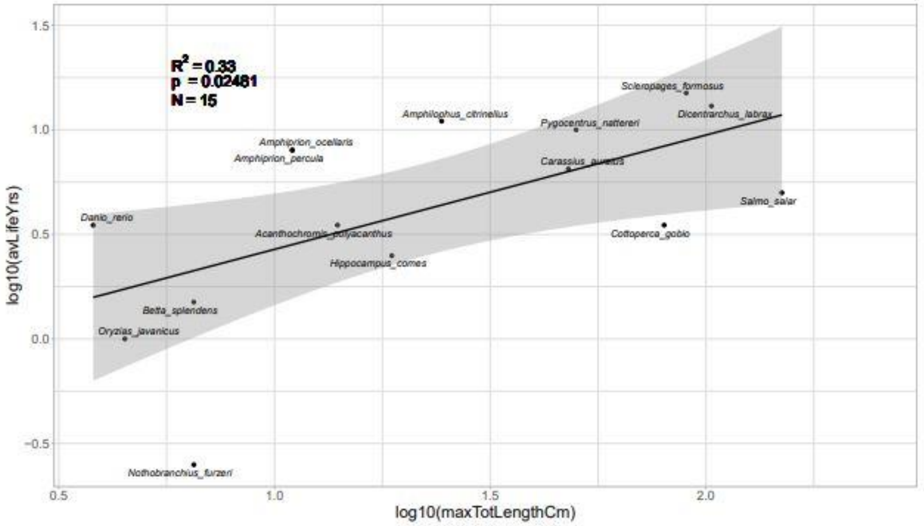

Figure 11. Linear regression between log transformed maximum body length and average lifespan. Each point in the plot represents a species. The line and the confidence intervals depicted in the plot come from standard linear regression, the values R2, p and N are from phylogenetically adjusted regression.

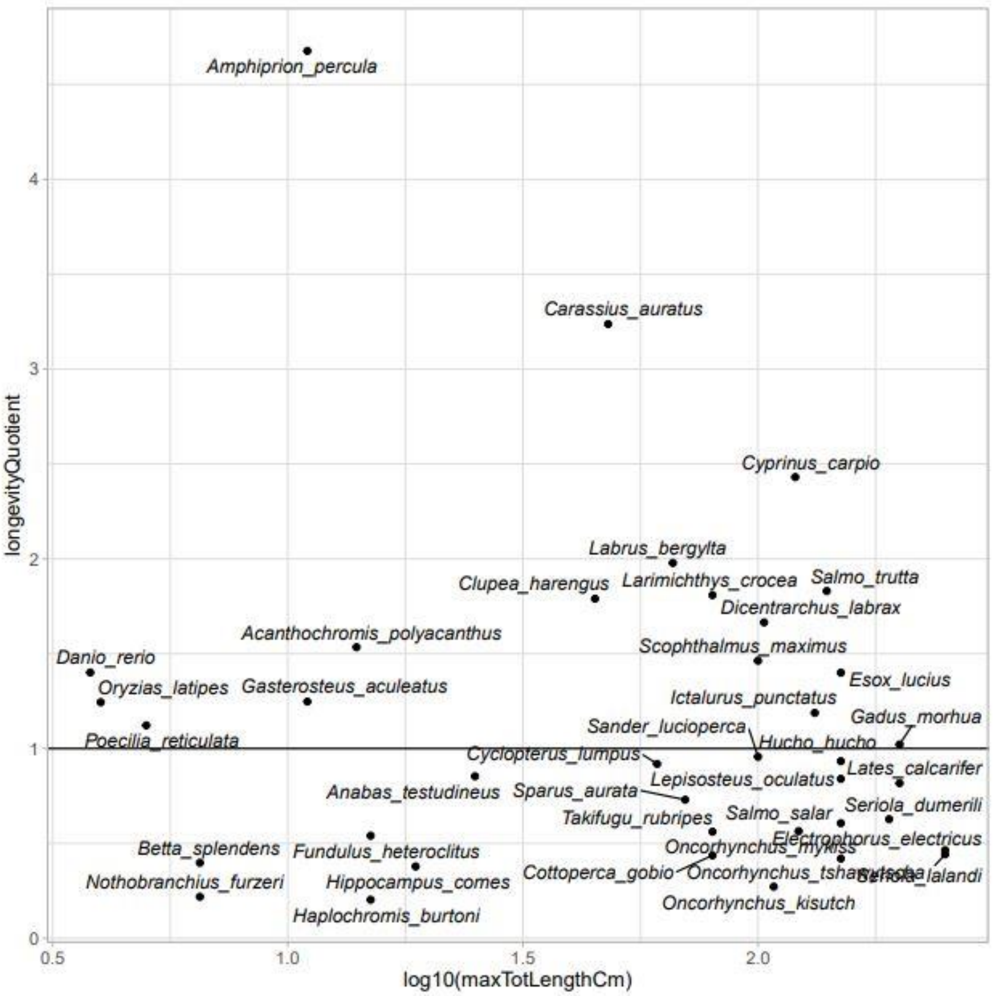

Figure S12. A plot between log transformed maximum body length and longevity quotient (LQ). Each point in the plot represents a species. To calculate the LQ we used the predict function on the model with only reliable ages (i.e. model from figure 2) similarly to Tollis et al. (2020).

Copy number variation (CNV) correlations of oncogenes an TSG's

The next set of plots are for depicting correlations between oncogene and tumour suppressor gene CNVs. In particular, the plots aim to visualize in what respect the obtained results depended on the method of getting copy number counts (CAFE vs ortholog) or subset of cancer genes (COSMIC tier 1 vs COSMIC tier 1&2).

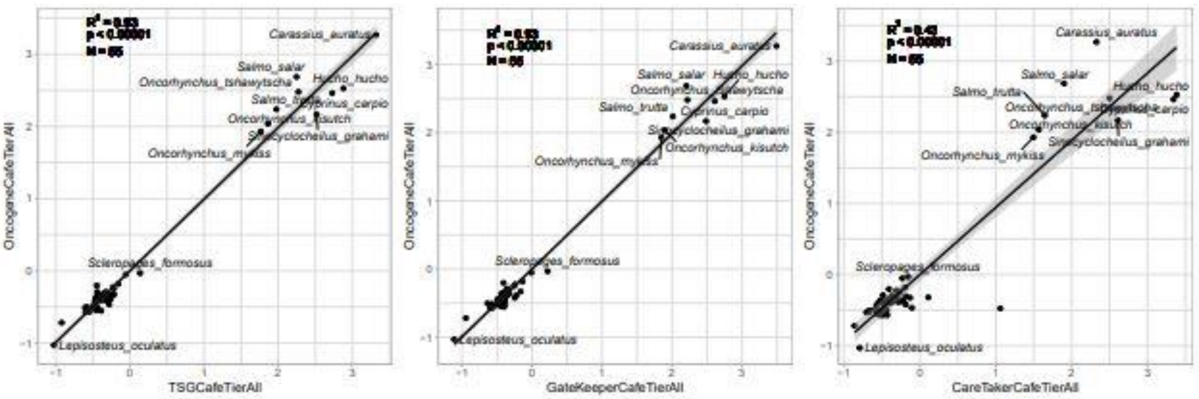

Figure S13. Linear regression between copy numbers of different subsets of tumour suppressor genes (all TSGs, gatekeeper genes and caretaker genes) and oncogene copy numbers. The CNVs have been obtained using the **CAFE** approach and both COSMIC **Tier 1&2** genes are included. Each point in the plot represents a species. The line and the confidence intervals depicted in the plot come from standard linear regression, the values R2, p and N are from phylogenetically adjusted regression.

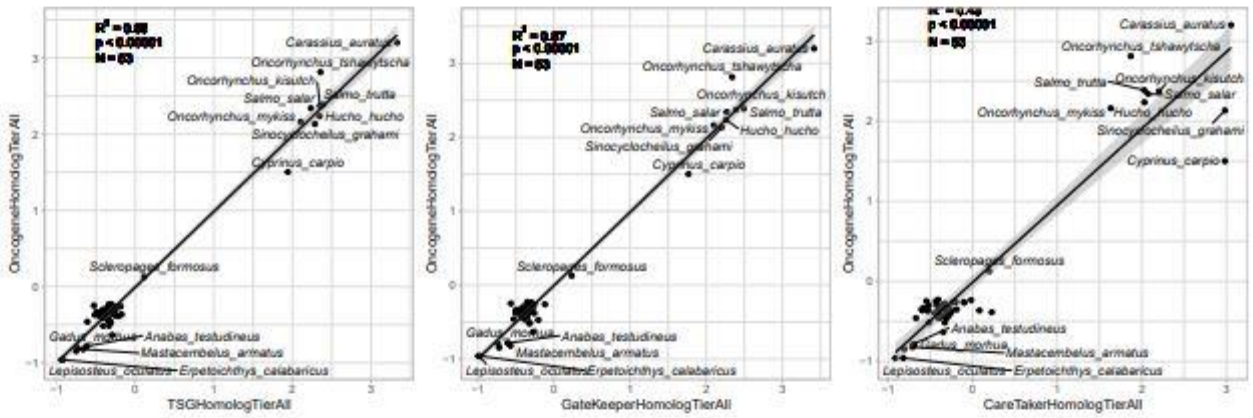

Figure S14. Linear regression between copy numbers of different subsets of tumor supressor genes (all TSGs, gatekeeper genes and caretaker genes) and oncogene copy numbers. The CNVs have been obtained using the **ortholog** approach and both COSMIC Tier 1&2 genes are included. Each point in the plot represents a species. The line and the confidence intervals depicted in the plot come from standard linear regression, the values R2, p and N are from phylogenetically adjusted regression.

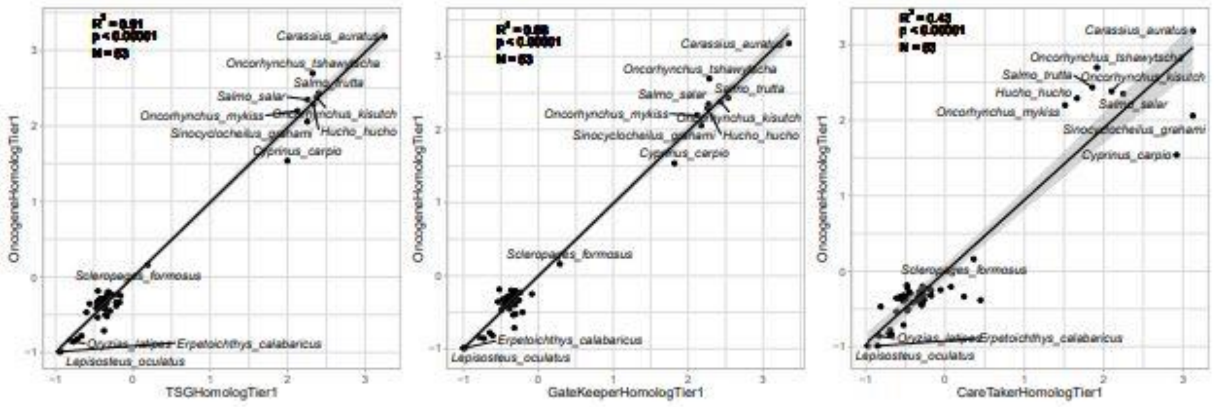

Figure S15. Linear regression between copy numbers of different subsets of tumor supressor genes (all TSGs, gatekeeper genes and caretaker genes) and oncogene copy numbers. The CNVs have been obtained using the **ortholog** approach and only COSMIC **Tier 1** genes are included. Each point in the plot represents a species. The line and the confidence intervals depicted in the plot come from standard linear regression, the values R2, p and N are from phylogenetically adjusted regression.

Cancer gene CNV vs lifepan

One central idea is that a higher number of TSGs and a lower number of oncogenes provides the basis for living longer. In the next section we correlate different CNVs with the magnitude of maximum lifespan. The CNVs are calculated using the CAFE approach on COSMIC Tier 1 cancer related genes.

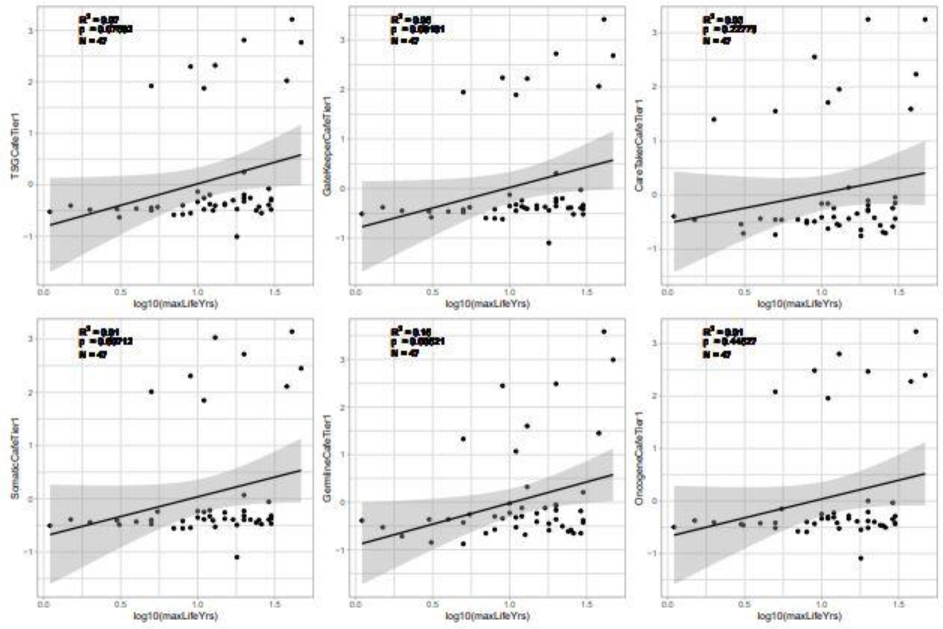

Figure S16. Linear regression between log maximum lifespan and copy numbers of different subsets of cancer related genes (TSG, GateKeeper, CareTaker, Somatic, Germline, Oncogene). The CNVs have been obtained using the **CAFE** approach and only COSMIC **Tier 1** genes are included. Each point in the plot represents a species in the dataset. The line and the confidence intervals depicted in the plot come from standard linear regression, the values R2, p and N are from phylogenetically adjusted regression.

Masked relationship between TSG copy numbers and lifespan

Checking the main idea of this paper, that the higher number of TSGs in longer lived fish is masked by the lower number of oncogenes in these fish. The copy numbers are calculated using the CAFE approach on COSMIC Tier 1 cancer related genes.

Table S1. Results from the phylogenetically adjusted regression with the formula *log10(maxLifeYrs) ~ OncogeneCafeTier1 + TSGCafeTier1*. The lambda, kappa and delta values are fixed at 1. The copy numbers have been obtained using the **CAFE** approach and only COSMIC T**ier 1** genes are included.

	Estimate	SE	t	p	
(Intercept)	1.17	0.38	3.07	0.0036	**
OncogeneCafeTier1	-1.23	0.35	-3.49	0.0011	**
TSGCafeTier1	1.37	0.35	3.94	3e-04	***

Table S2. Results from the phylogenetically adjusted regression with the formula *log10(maxLifeYrs) ~ OncogeneCafeTier1 + TSGCafeTier1*. The lambda, kappa and delta values have been optimized using maximum likelihood. The copy numbers have been obtained using the **CAFE** approach and only COSMIC **Tier 1** genes are included.

	Estimate	SE	t	p	
(Intercept)	1.2	0.06	19.95	<0.0001	***
OncogeneCafeTier1	-0.7	0.27	-2.64	0.011	*
TSGCafeTier1	0.71	0.25	2.89	0.006	**
	kappa	lambda	delta		
	0.16	1	3		

Table S3. Results from the phylogenetically adjusted regression with the formula *log10(maxLifeYrs) ~ log10(maxTotLengthCm) + OncogeneCafeTier1 + , TSGCafeTier1*. The lambda, kappa and delta values are fixed at 1. The copy numbers have been obtained using the **CAFE** approach and only COSMIC **Tier 1** genes are included.

	Estimate	SE	t	p	
(Intercept)	0.81	0.49	1.67	0.102	

log10(maxTotLengthCm)	0.19	0.16	1.17	0.25	
OncogeneCafeTier1	-1.19	0.35	-3.36	0.0016	**
TSGCafeTier1	1.31	0.35	3.73	6e-04	***

Table S4. Results from the phylogenetically adjusted regression with the formula *log10(maxLifeYrs) ~ log10(maxTotLengthCm) + OncogeneCafeTier1 + , TSGCafeTier1*. The lambda, kappa and delta values have been optimized using maximum likelihood. The copy numbers have been obtained using the **CAFE** approach and only COSMIC **Tier 1** genes are included.

	Estimate	SE	t	p	
(Intercept)	0.59	0.16	3.64	7e-04	***
log10(maxTotLengthCm)	0.31	0.08	3.95	3e-04	***
OncogeneCafeTier1	-0.49	0.22	-2.2	0.033	*
TSGCafeTier1	0.52	0.2	2.59	0.013	*
	kappa	lambda	delta		
	0.06	0.91	3		

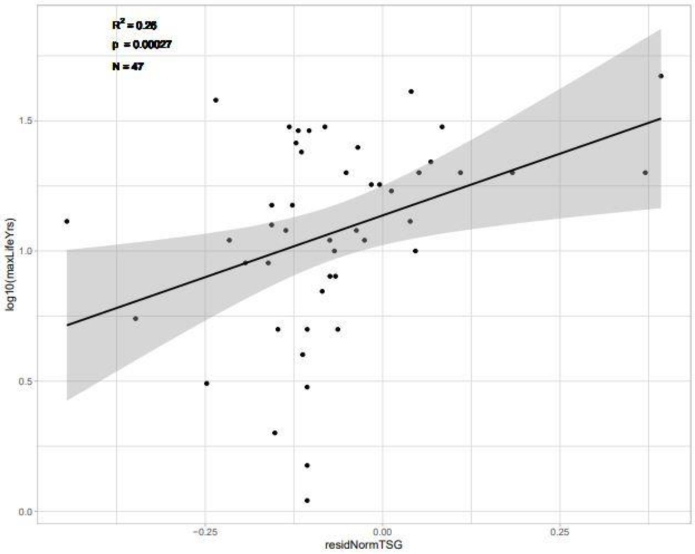

Figure S17. Linear regression between log maximum lifespan and the residual copy numbers of TSGs. The residual TSG CNVs have been obtained using the phylogenetically adjusted regression between TSG and oncogene CNVs. The **CAFE** approach and only COSMIC **Tier 1** genes are included. Each point in the plot represents a species in the dataset. The line and the confidence intervals depicted in the plot come from standard linear regression, the values R2, p and N are from phylogenetically adjusted regression.

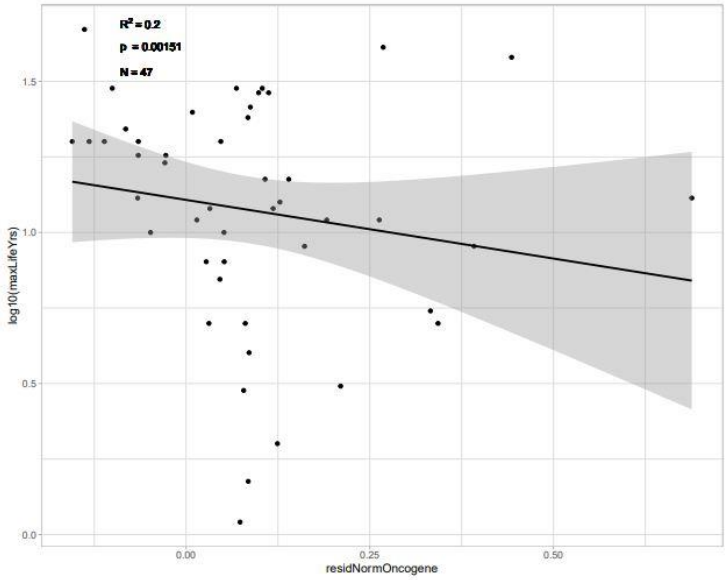

Figure S18. Linear regression between log maximum lifespan and the residual copy numbers of TSGs. The residual TSG CNVs have been obtained using the phylogenetically adjusted regression between TSG and oncogene CNVs. The **CAFE** approach and only COSMIC **Tier 1** genes are included. Each point in the plot represents a species in the dataset. The line and the confidence intervals depicted in the plot come from standard linear regression, the values R2, p and N are from phylogenetically adjusted regression.

All of the above relationships also hold (and are even more confident) in the dataset with only 36 species (only data from AnAge, fishbase and articles).

Body size vs cancer gene CNVs

To reproduce the plots in figure 4 of Tollis et al. (2020) on our fish dataset, we correlated the body size measures with normalized cancer related gene counts also on our fish dataset.

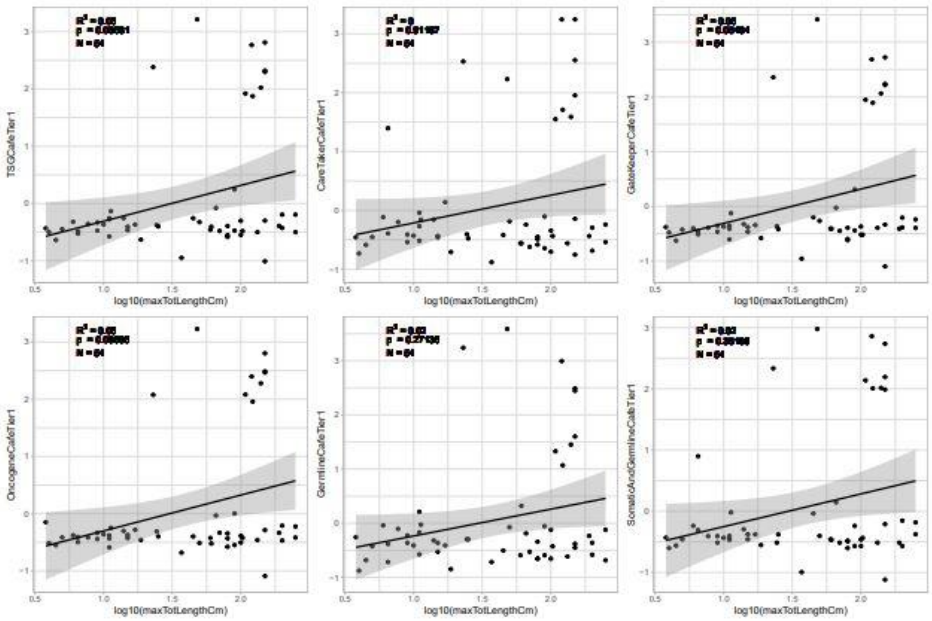

Figure S19. Linear regression between log maximum body size and copy numbers of different subsets of cancer related genes (TSG, CareTaker, GateKeeper, Oncogene, Germline, SomaticAndGermline). The CNVs have been obtained using the **CAFE** approach and only COSMIC **Tier 1** genes are included. Each point in the plot represents a species in the dataset. The line and the confidence intervals depicted in the plot come from standard linear regression, the values R2, p and N are from phylogenetically adjusted regression.

Also, let us check for a masked relationship as TSG and oncogene counts are likely evolutionary constrained.

Table S5. Results from the phylogenetically adjusted regression with the formula *log10(maxTotLengthCm) ~ OncogeneCafeTier1 + TSGCafeTier1*. The lambda, kappa and delta values are fixed at 1. The CNVs have been obtained using the **CAFE** approach and only COSMIC **Tier 1** genes are included.

	Estimate	SE	t	p	
(Intercept)	1.87	0.42	4.47	<0.0001	***
OncogeneCafeTier1	-0.09	0.31	-0.3	0.76	
TSGCafeTier1	0.26	0.31	0.85	0.4	
	kappa	lambda	delta		
	1	1	1		

Clearly, we are unable to demonstrate that body length depends on oncogene or TSG copy numbers.

Longevity Quotient (LQ) vs cancer gene CNVs

The phylogenetically informed regression indicates a stronger positive correlation between size and lifespan only on highly confident data (see above section **Lifespan vs size**). Therefore, at first the LQ was calculated using this model. In the plots we plot the same correlations between copy numbers and LQ that we did for maximum body size and lifespan for only these species that we have reasonably confident age estimate available.

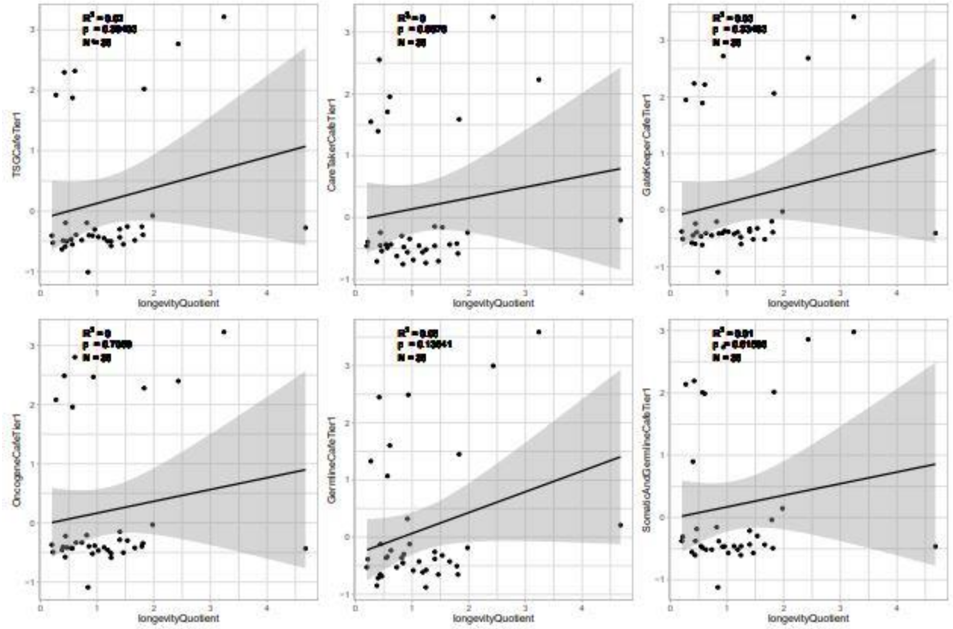

Figure S20. Linear regression between longevity quotient (LQ) and copy numbers for different subsets of cancer related genes (TSG, CareTaker, GateKeeper, Oncogene, Germline, SomaticAndGermline). The CNVs have been obtained using the **CAFE** approach and only COSMIC **Tier 1** genes are included. The LQs have been obtained using the same method as that of Tollis et al. (2020) Each point in the plot represents a species in the dataset. The line and the confidence intervals depicted in the plot come from standard linear regression, the values R2, p and N are from phylogenetically adjusted regression.

Masked relationship between TSG CNV and LQ

Checking the main idea in this paper that the higher number of TSGs in longer lived fish is masked by the lower number of oncogenes in said fish. The CNVs are calculated using the CAFE approach on COSMIC Tier 1 cancer related genes. The estimates are calculated for both more confident maximum lifespan and all available maximum lifespan.

Table S6. Results from the phylogenetically adjusted regression with the formula *longevityQuotient ~ OncogeneCafeTier1 + TSGCafeTier1*. The lambda, kappa and delta values are fixed at 1. The CNVs have been obtained using the **CAFE** approach and only COSMIC **Tier 1** genes are included. Only species with maximum lifespan from most trustworthy sources were included (N=38).

	Estimate	SE	t	p

(Intercept)	1.17	0.93	1.25	0.22	
OncogeneCafeTier1	-1.44	0.88	-1.63	0.112	
TSGCafeTier1	1.59	0.88	1.81	0.078	.

Table S7. Results from the phylogenetically adjusted regression with the formula *longevityQuotient ~ OncogeneCafeTier1 + TSGCafeTier1*. The lambda, kappa and delta values have been optimized using maximum likelihood. The CNVs have been obtained using the **CAFE** approach and only COSMIC **Tier 1** genes are included. Only species with maximum lifespan from most trustworthy sources were included (N=38).

	Estimate	SE	t	p	
(Intercept)	1.13	0.16	7.17	<0.0001	***
OncogeneCafeTier1	-1.09	0.78	-1.39	0.173	
TSGCafeTier1	1.33	0.78	1.7	0.098	.
	kappa	lambda	delta		
	0.46	0.79	3		

Table S8. Results from the phylogenetically adjusted regression with the formula *longevityQuotient ~ OncogeneCafeTier1 + TSGCafeTier1*. The lambda, kappa and delta values are fixed at 1. The CNVs have been obtained using the **CAFE** approach and only COSMIC **Tier 1** genes are included. Species with maximum lifespan from all sources were included (N=47).

	Estimate	SE	t	p	
(Intercept)	1.04	1.14	0.91	0.37	
OncogeneCafeTier1	-2.53	1.06	-2.39	0.021	*
TSGCafeTier1	2.73	1.04	2.61	0.012	*

Table S9. Results from the phylogenetically adjusted regression with the formula *longevityQuotient ~ OncogeneCafeTier1 + TSGCafeTier1*. The lambda, kappa and delta values have been optimized using maximum likelihood. The CNVs have been obtained using the **CAFE** approach and only COSMIC **Tier 1** genes are included. Species with maximum lifespan from all sources were included (N=47).

	Estimate	SE	t	p	
(Intercept)	0.99	0.14	7.19	<0.0001	***
OncogeneCafeTier1	-0.69	0.62	-1.13	0.27	
TSGCafeTier1	0.89	0.56	1.58	0.121	
	kappa	lambda	delta		
	0	1	2.83		

Conclusions

The analyses above suggest that the magnitude of maximum lifespan of fish species is positively affected by the total copy number of tumour suppressor genes and negatively by the total number of oncogenes. As TSG and oncogene CNV is strongly correlated, their relationship with lifespan is masked if one is excluded from the model. The connection between lifespan and TSG CNV is particularly robust as it does not depend much on model parameters inclusion and exclusion of some data points or whether the lifespan is adjusted for body size or the method of obtaining CNV counts.

References

Tollis M, Schneider-Utaka AK, Maley CC. 2020. The Evolution of Human Cancer Gene Duplications across Mammals. Molecular Biology and Evolution 37, 2875–2886.

S5 Copy Number Regression Plots of Fish Cancer Genes Without Salmonids and Cyprinids

Introduction

This supplementum aims to plot some graphs and tables for referencing in the main text. These plots and tables are meant to clarify and illustrate the data and analyses. The plots differ from the ones in the main article mainly by including only all teleost fish which have gone through three rounds of whole-genome duplication (WGD). This means ray-finned fishes (Class: *Actinopterygii*) without salmonids and cyprinids.

Lifespan vs size

First let's plot to see if the magnitude of maximum lifespan is related to magnitude in maximum body length in all fish species. The underling hypothesis is that species who live longer also tend to be larger.

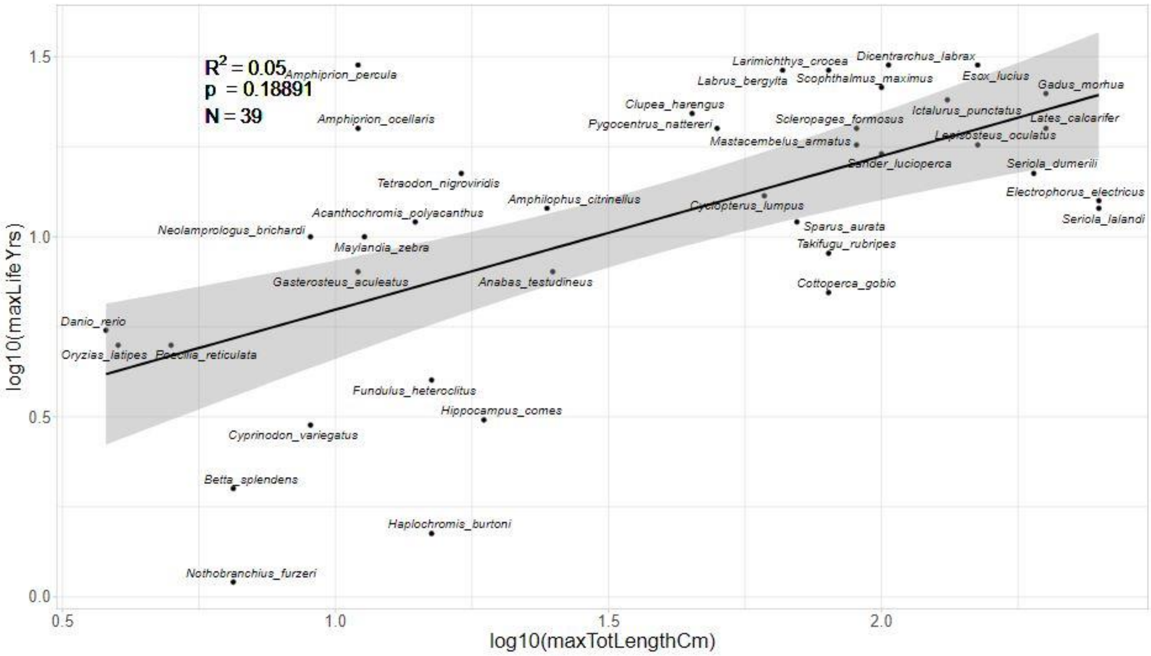

Figure S21. Linear regression between log transformed maximum body length and maximum lifespan. Each point in the plot represents a species. The line and the confidence intervals depicted in the plot come from standard linear regression, the values R2, p and N are from phylogenetically adjusted regression.

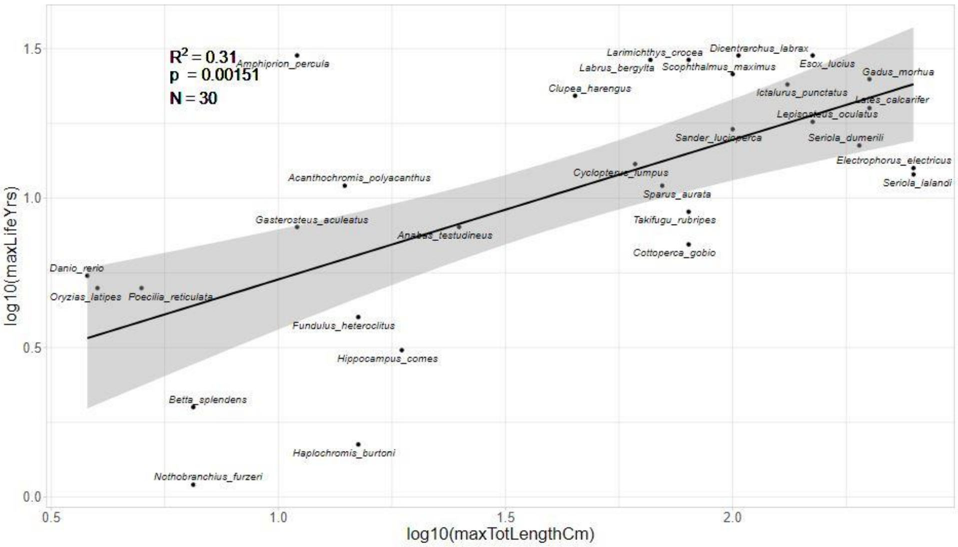

Figure S22. Linear regression between log transformed maximum body length and maximum lifespan. Each point in the plot represents a species. Only species for whom the lifespan and body size data is extracted from AnAge database, fishbase or articles is included. The line and the confidence intervals depicted in the plot come from standard linear regression, the values R^2, p and N are from phylogenetically adjusted regression.

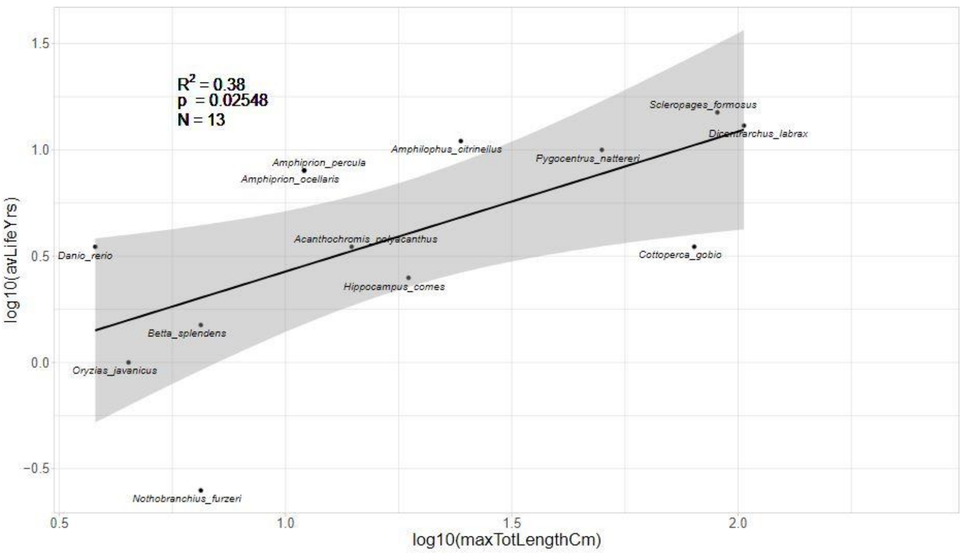

Figure S23. Linear regression between log transformed maximum body length and average lifespan. Each point in the plot represents a species. The line and the confidence intervals depicted in the plot come from standard linear regression, the values R^2, p and N are from phylogenetically adjusted regression.

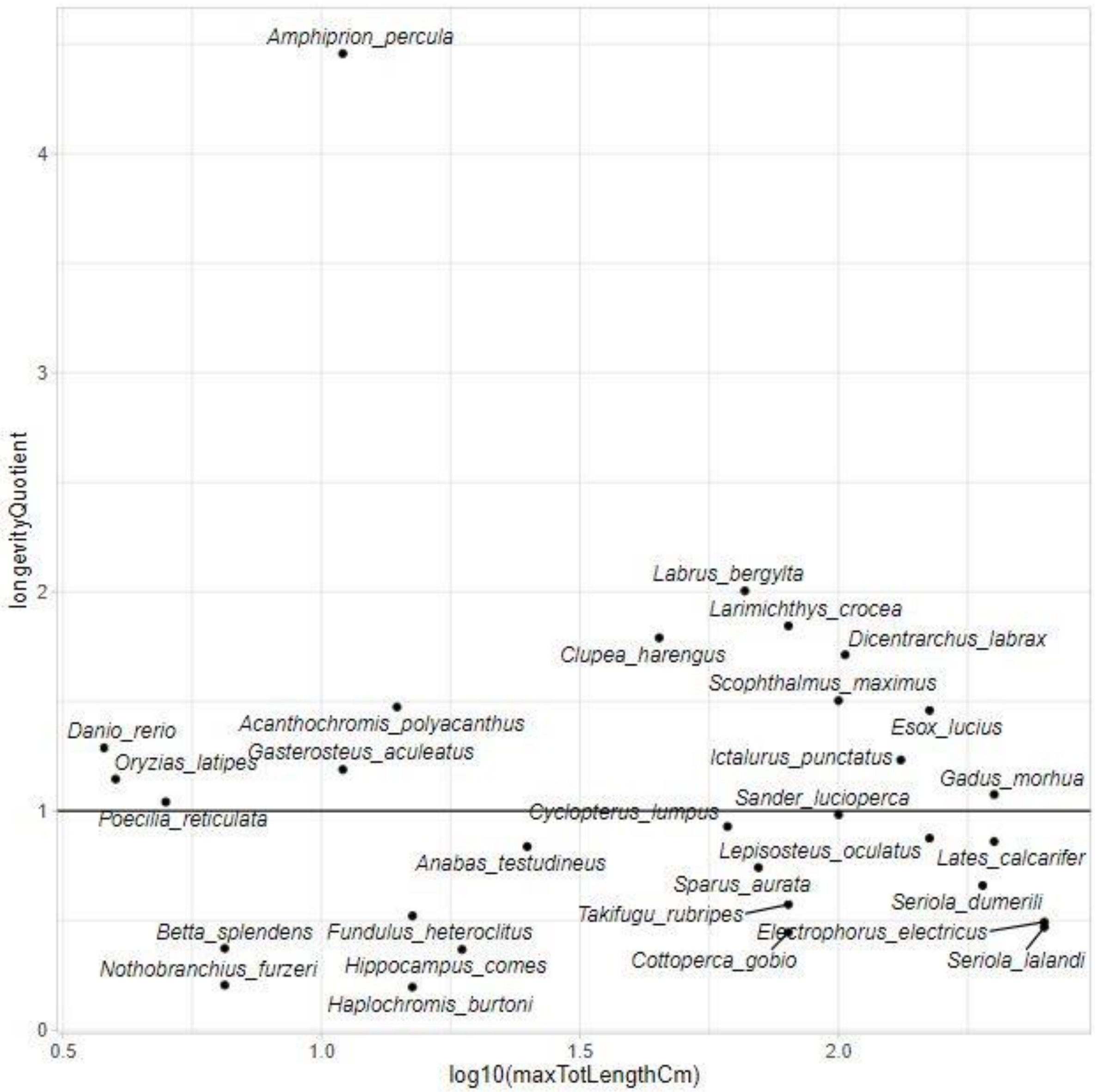

Figure S24. A plot between log transformed maximum body length and longevity quotient (LQ). Each point in the plot represents a species. To calculate the LQ we used the predict function on the model with only reliable ages (i.e. model from figure 2) similarly to Tollis et al. (2020).

Copy number variation (CNV) correlations of oncogenes an TSG's

The next set of plots are for depicting correlations between oncogene and tumour suppressor gene CNVs. In particular the plots aim to visualize in what respect the obtained results depended on the method used for getting copy number counts (CAFE vs ortholog) or subset of cancer genes (COSMIC tier 1 vs COSMIC tier 1&2).

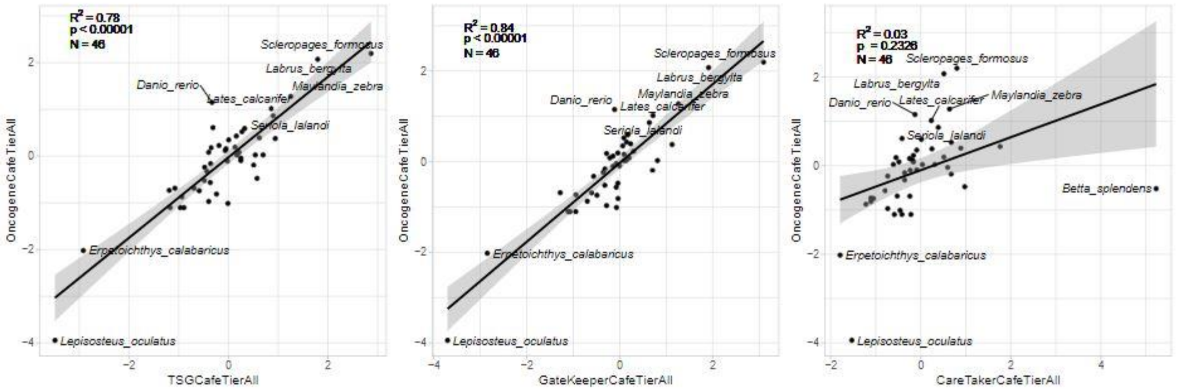

Figure S25. Linear regression between copy numbers of different subsets of tumour suppressor genes (all TSGs, gatekeeper genes and caretaker genes) and oncogenes. The copy numbers have been obtained using the **CAFE** approach and both COSMIC **Tier 1&2** genes are included. Each point in the plot represents a species. The line and the confidence intervals depicted in the plot come from standard linear regression, the values R^2, p and N are from phylogenetically adjusted regression.

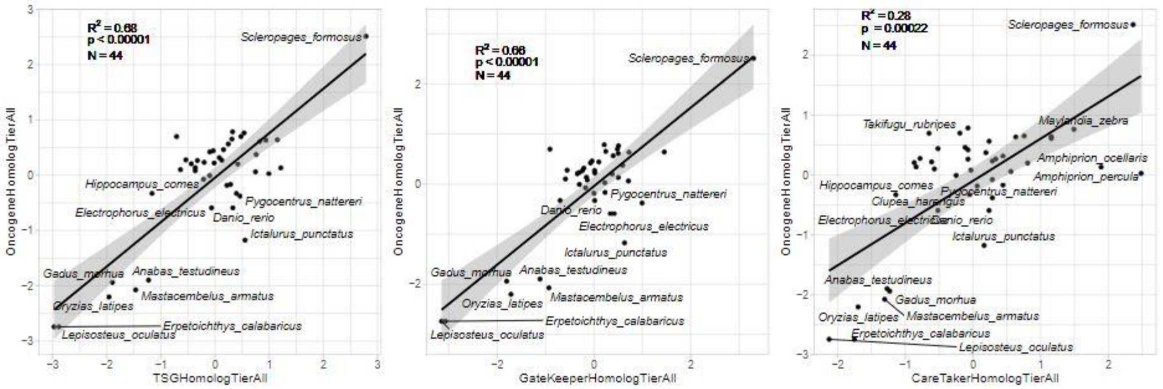

Figure S26. Linear regression between copy numbers of different subsets of tumour suppressor genes (all TSGs, gatekeeper genes and caretaker genes) and oncogenes. The copy numbers have been obtained using the **ortholog** approach and both COSMIC **Tier 1&2** genes are included. Each point in the plot represents a species. The line and the confidence intervals depicted in the plot come from standard linear regression, the values R^2, p and N are from phylogenetically adjusted regression.

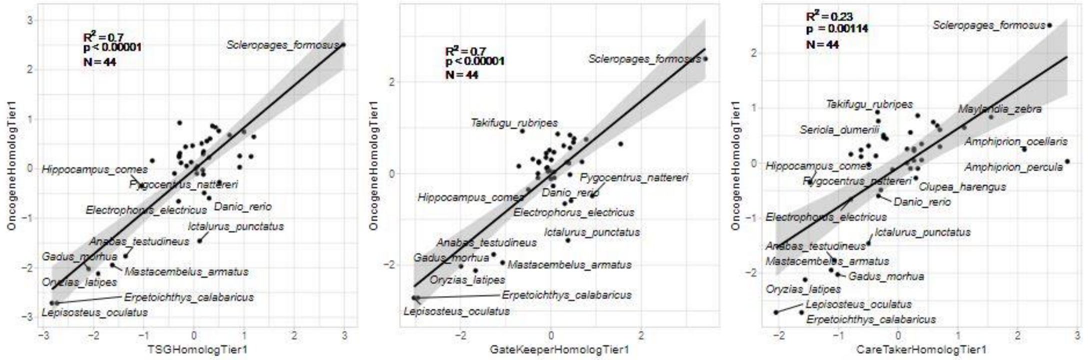

Figure S27. Linear regression between copy numbers of different subsets of tumour suppressor genes (all TSGs, gatekeeper genes and caretaker genes) and oncogenes. The copy numbers have been obtained using the **ortholog** approach and only COSMIC **Tier 1** genes are included. Each point in the plot represents a species. The line and the confidence intervals depicted in the plot come from standard linear regression, the values R^2, p and N are from phylogenetically adjusted regression.

Cancer gene CNV vs lifepan

One central idea is that the higher number of TSGs and the lower number of oncogenes provides the basis to live longer. In the next section we correlate different CNVs with the magnitude of maximum lifespan. The copy numbers are calculated using the CAFE approach on COSMIC Tier 1 cancer related genes.

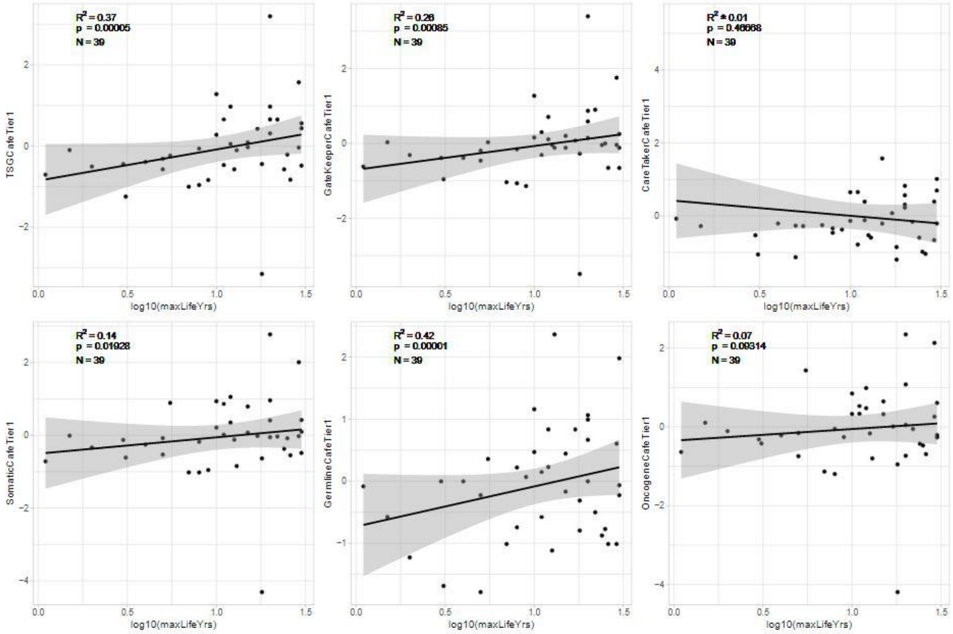

Figure S28. Linear regression between log maximum lifespan and copy numbers of different subsets of cancer related genes (TSG, GateKeeper, CareTaker, Somatic, Germline, Oncogene). The copy numbers have been obtained using the **CAFE** approach and only COSMIC **Tier 1** genes are included. Each point in the plot represents a species in the dataset. The line and the confidence intervals depicted in the plot come from standard linear regression, the values R^2, p and N are from phylogenetically adjusted regression.

Masked relationship between TSG CNV and lifespan

Checking the main idea of this paper that the higher number of TSGs in longer lived fish is masked by the lower number of oncogenes in these fish. The copy numbers are calculated using the **CAFE** approach on COSMIC **Tier 1** cancer related genes.

Table S10. Results from the phylogenetically adjusted regression with the formula *log10(maxLifeYrs) ~ OncogeneCafeTier1 + TSGCafeTier1*. The lambda, kappa and delta values are fixed at 1. The copy numbers have been obtained using the **CAFE** approach and only COSMIC **Tier 1** genes are included.

	Estimate	SE	t	p

(Intercept)	1.11	0.31	3.59	0.001	***
OncogeneCafeTier1	-0.41	0.11	-3.54	0.0011	**
TSGCafeTier1	0.61	0.1	5.9	<0.0001	***

Table S11. Results from the phylogenetically adjusted regression with the formula *log10(maxLifeYrs) ~ OncogeneCafeTier1 + TSGCafeTier1*. The lambda, kappa and delta values have been optimized using maximum likelihood. The copy numbers have been obtained using the **CAFE** approach and only COSMIC **Tier 1** genes are included.

	Estimate	SE	t	p	
(Intercept)	1.1	0.07	14.8	<0.0001	***
OncogeneCafeTier1	-0.22	0.06	-3.44	0.0015	**
TSGCafeTier1	0.24	0.07	3.62	9e-04	***
	kappa	lambda	delta		
	0	1	2.24		

Table S12. Results from the phylogenetically adjusted regression with the formula *log10(maxLifeYrs) ~ OncogeneCafeTier1 + TSGCafeTier1*. The lambda, kappa and delta values are fixed at 1. The copy numbers have been obtained using the **CAFE** approach and only COSMIC **Tier 1** genes are included.

	Estimate	SE	t	p	
(Intercept)	0.77	0.39	1.99	0.054	.
log10(maxTotLengthCm)	0.19	0.13	1.43	0.162	
OncogeneCafeTier1	-0.39	0.11	-3.39	0.0017	**
TSGCafeTier1	0.6	0.1	5.8	<0.0001	***

Table S13. Results from the phylogenetically adjusted regression with the formula *log10(maxLifeYrs) ~ log10(maxTotLengthCm) + OncogeneCafeTier1 + , TSGCafeTier1*. The lambda, kappa and delta values have been optimized using maximum likelihood. The copy numbers have been obtained using the **CAFE** approach and only COSMIC **Tier 1** genes are included.

	Estimate	SE	t	p	
(Intercept)	0.45	0.13	3.35	0.002	**
log10(maxTotLengthCm)	0.39	0.08	5.1	<0.0001	***
OncogeneCafeTier1	-0.05	0.05	-1.13	0.27	
TSGCafeTier1	0.09	0.05	1.66	0.106	
	kappa	lambda	delta		
	0	0	2.16		

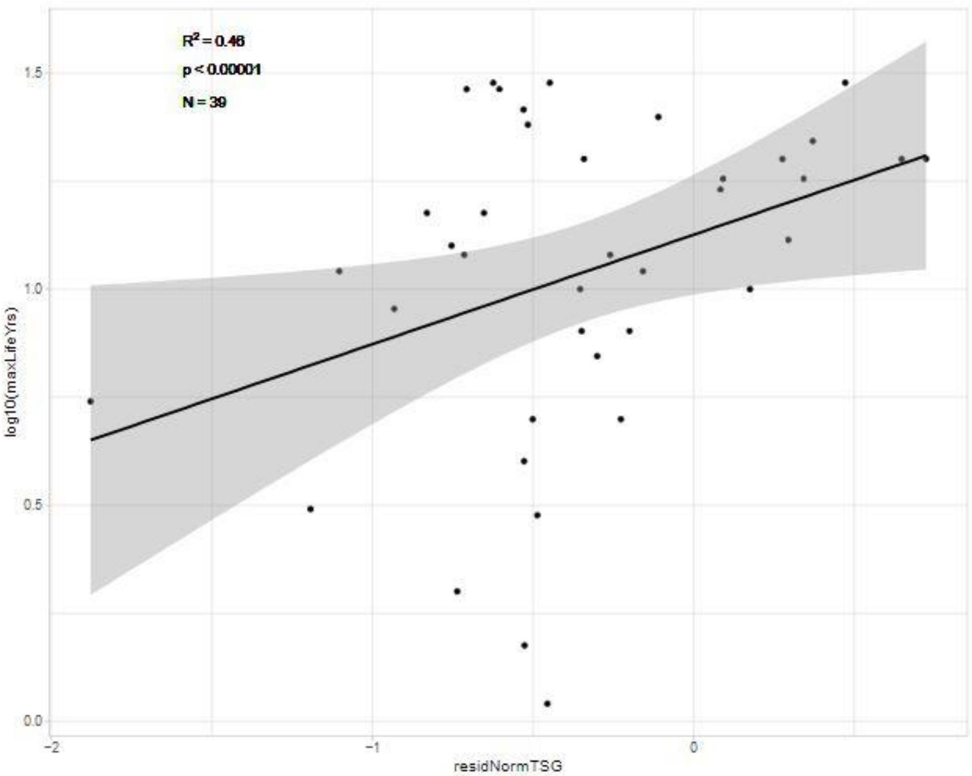

Figure S29. Linear regression between log maximum lifespan and the residual copy numbers of TSGs. The residual TSG copy numbers have been obtained using the phylogenetically adjusted regression between TSG and oncogene copy numbers. The **CAFE** approach and only COSMIC **Tier 1** genes are included. Each point in the plot represents a species in the dataset. The line and the confidence intervals depicted in the plot come from standard linear regression, the values R^2, p and N are from phylogenetically adjusted regression.

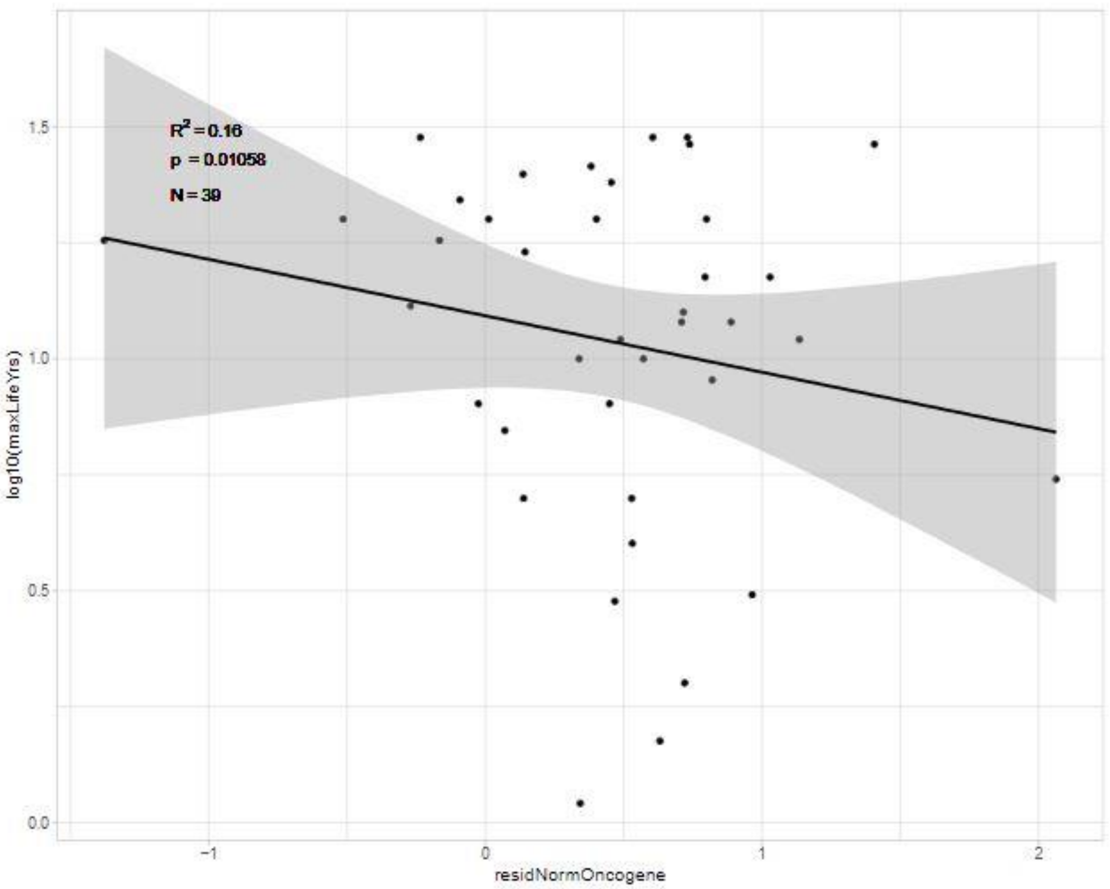

Figure S30. Linear regression between log maximum lifespan and the residual copy numbers of TSGs. The residual TSG copy numbers have been obtained using the phylogenetically adjusted regression between TSG and oncogene copy numbers. The **CAFE** approach and only COSMIC **Tier 1** genes are included. Each point in the plot represents a species in the dataset. The line and the confidence intervals depicted in the plot come from standard linear regression, the values R^2, p and N are from phylogenetically adjusted regression.

All of the above relationships also hold (and are even more confident) in the dataset with only 40 species (only data from AnAge, fishbase and articles).

Body size vs cancer gene CNVs

To reproduce the plots in figure 4 of Tollis et al. (2020) on our fish dataset, we correlated the body size measures with normalized cancer related gene counts also on our fish dataset.

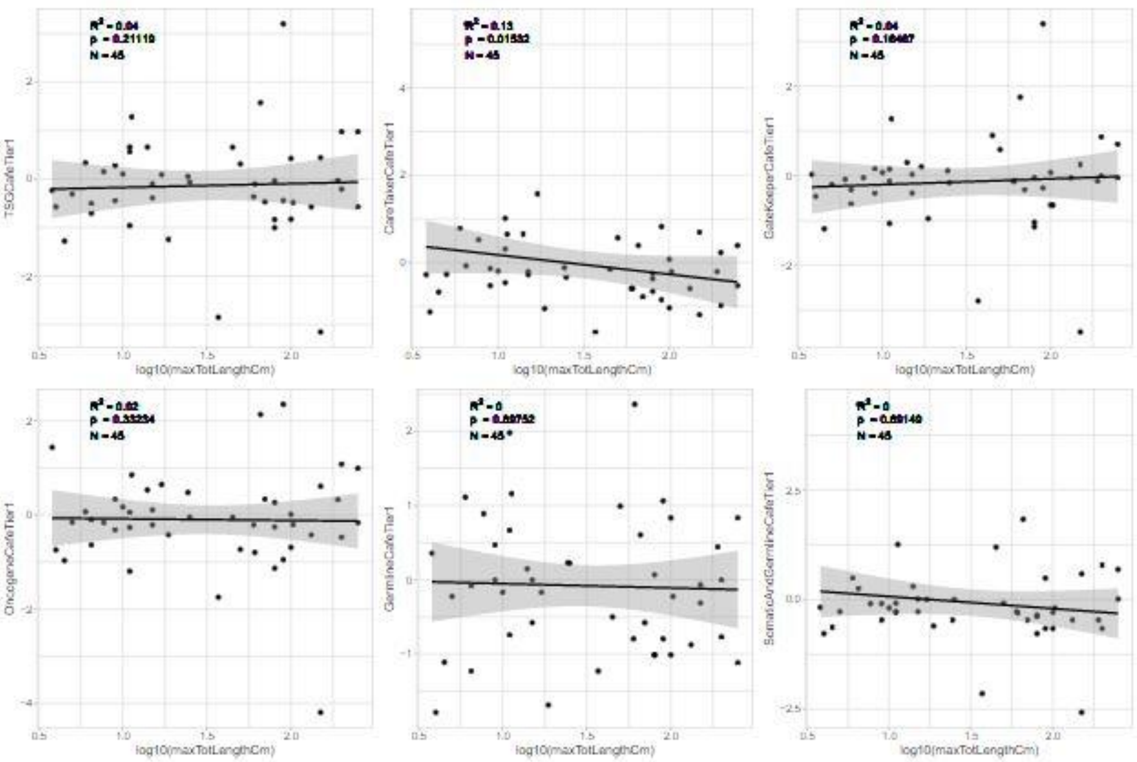

Figure S31. Linear regression between log maximum body size and copy numbers of different subsets of cancer related genes (TSG, CareTaker, GateKeeper, Oncogene, Germline, SomaticAndGermline). The copy numbers have been obtained using the **CAFE** approach and only COSMIC **Tier 1** genes are included. Each point in the plot represents a species in the dataset. The line and the confidence intervals depicted in the plot come from standard linear regression, the values R^2, p and N are from phylogenetically adjusted regression.

Also, let’s check for a masked relationship as TSG and oncogene counts are likely evolutionarily constrained.

Table S14. Results from the phylogenetically adjusted regression with the formula *log10(maxTotLengthCm) ~ OncogeneCafeTier1 + TSGCafeTier1*. The lambda, kappa and delta values are fixed at 1. The copy numbers have been obtained using the **CAFE** approach and only COSMIC **Tier 1** genes are included.

	Estimate	SE	t	p	
(Intercept)	1.86	0.44	4.19	1e-04	***
OncogeneCafeTier1	-0.04	0.13	-0.27	0.79	
TSGCafeTier1	0.1	0.12	0.83	0.41	
	kappa	lambda	delta		
	1	1	1		

Clearly, we were unable to demonstrate that body length depends on oncogene or TSG copy numbers.

Longevity Quotient (LQ) vs cancer gene copy numbers

The phylogenetically informed regression indicates a stronger positive correlation between size and lifespan only on high confident data (see above section **Lifespan vs size**). Therefore, the first the LQ was calculated using this model. In the plots we show the same correlations between copy numbers and LQ that we did for maximum body size and lifespan, for only the species that we have reasonably confident age estimate available for.

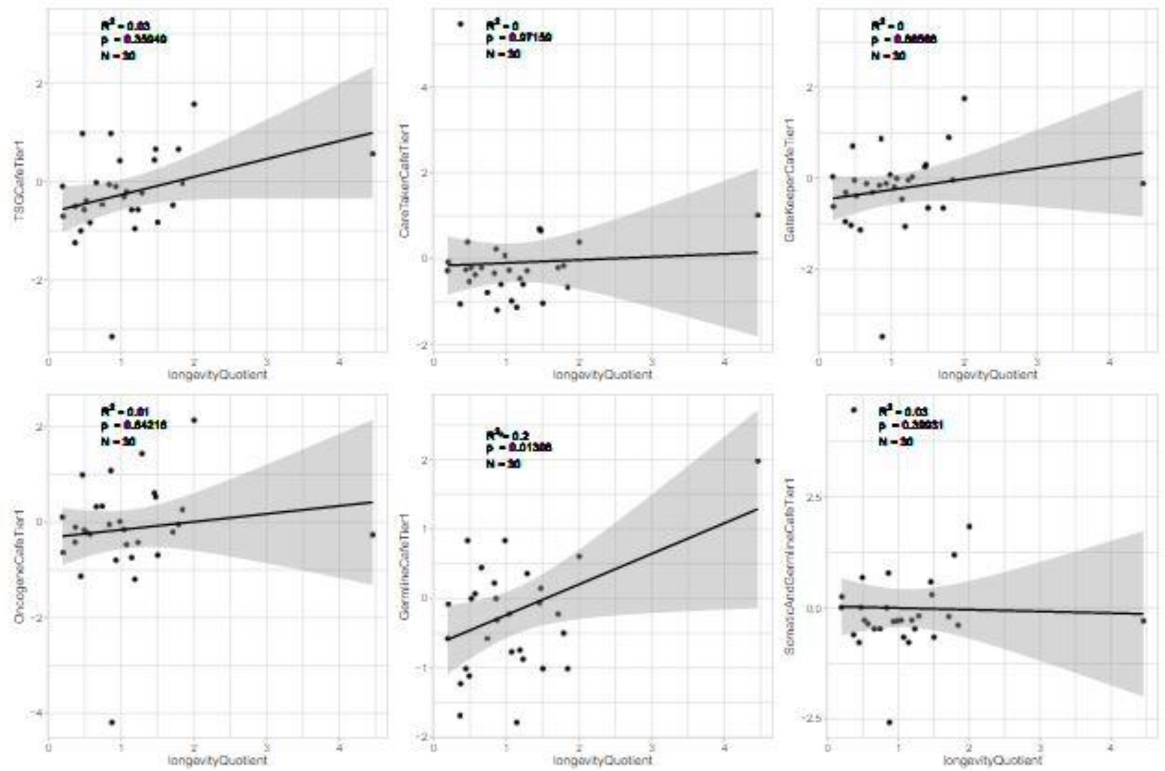

Figure S32. Linear regression between longevity quotient (LQ) and copy numbers of different subsets of cancer related genes (TSG, CareTaker, GateKeeper, Oncogene, Germline, SomaticAndGermline). The copy numbers have been obtained using the **CAFE** approach and only COSMIC **Tier 1** genes are included. The LQ have been obtained using the same method as Tollis et al. (2020) Each point in the plot represents a species in the dataset. The line and the confidence intervals depicted in the plot come from standard linear regression, the values R^2, p and N are from phylogenetically adjusted regression.

Masked relationship between TSG copy numbers and LQ

Checking the main idea of this paper that the higher number of TSGs in longer lived fish is masked by the lower number of oncogenes in these fish. The copy numbers are calculated using the CAFE approach on COSMIC Tier 1 cancer related genes. The estimates are calculated for both more confident maximum lifespan and all available maximum lifespan.

Table S15. Results from the phylogenetically adjusted regression with the formula *longevityQuotient ~ OncogeneCafeTier1 + TSGCafeTier1*. The lambda, kappa and delta values are fixed at 1. The copy numbers have been obtained using the **CAFE** approach and only COSMIC

Tier 1 genes are included. Only species with maximum lifespan from most trustworthy sources (N=30).

	Estimate	SE	t	p	
(Intercept)	1.11	0.85	1.31	0.2	
OncogeneCafeTier1	-0.91	0.37	-2.44	0.021	*
TSGCafeTier1	1.07	0.41	2.59	0.015	*

Table S16. Results from the phylogenetically adjusted regression with the formula *longevityQuotient ~ OncogeneCafeTier1 + TSGCafeTier1*. The lambda, kappa and delta values have been optimized using maximum likelihood. The copy numbers have been obtained using the **CAFE** approach and only COSMIC **Tier 1** genes are included. Only species with maximum lifespan from most trustworthy sources are included (N=30).

	Estimate	SE	t	p	
(Intercept)	1.35	0.09	14.33	<0.0001	***
OncogeneCafeTier1	-0.3	0.12	-2.54	0.017	*
TSGCafeTier1	0.55	0.16	3.48	0.0017	**
	kappa	lambda	delta		
	0	0.41	3		

Table S17. Results from the phylogenetically adjusted regression with the formula *longevityQuotient ~ OncogeneCafeTier1 + TSGCafeTier1*. The lambda, kappa and delta values are fixed at 1. The copy numbers have been obtained using the **CAFE** approach and only COSMIC **Tier 1** genes are included. Species with maximum lifespan from all sources are included (N=39).

	Estimate	SE	t	p	
(Intercept)	0.7	1.02	0.69	0.5	
OncogeneCafeTier1	-1.28	0.38	-3.39	0.0017	**
TSGCafeTier1	1.4	0.34	4.09	2e-04	***

Table S18. Results from the phylogenetically adjusted regression with the formula *longevityQuotient ~ OncogeneCafeTier1 + TSGCafeTier1*. The lambda, kappa and delta values have been optimized using maximum likelihood. The copy numbers have been obtained using the **CAFE** approach and only COSMIC **Tier 1** genes are included. Species with maximum lifespan from all sources are included (N=39).

	Estimate	SE	t	p	
(Intercept)	1.12	0.08	13.22	<0.0001	***
OncogeneCafeTier1	0.01	0.1	0.12	0.9	
TSGCafeTier1	0.06	0.11	0.57	0.57	
	kappa	lambda	delta		
	0	0	2.56		

Conclusions

The analyses above suggest that the magnitude of maximum lifespan of fish species is positively affected by the total copy number of tumour suppressor genes when species with WGD have been removed. As TSG and oncogene copy numbers are strongly correlated, TSG relationship with lifespan is masked if one is excluded from the model. However, the connection between lifespan

and TSG copy numbers is not particularly robust as it does depend somewhat on model parameters inclusion and exclusion of some data points or whether the lifespan is adjusted for body size (i.e. longevity quotient) or not.

References

Tollis M, Schneider-Utaka AK, Maley CC. 2020. The Evolution of Human Cancer Gene Duplications across Mammals. Molecular Biology and Evolution 37, 2875–2886.

S6 Copy Number Regressions for Mammal Cancer Genes

Introduction

The idea of this supplementum is to do basic phylogenetically informed regressions of cancer gene copy numbers of mammal genes. This analysis is aimed to check if the masked relationship detected in this papers fish datasets holds for the mammal dataset. The cancer genes are from the COSMIC (version 92) database (Sondka et al. 2018). The mammals are those that have a sequenced genome in the Ensembl (v104) database and have a CAFE tree associated with them in Ensembl Compara (Herrero et al. 2016). This report is inspired by the paper on copy number variation (CNV) in mammals by Tollis et al. (2020). While the aforementioned paper used 63 mammal genomes this report uses 108 species (only 33 species overlap between the 2 datasets).

Data

The copy numbers are obtained for two sets of cancer related genes. The first is the COSMIC tier 1 genes and the second included both COSMIC tier 1 and tier 2 genes. COSMIC is a manually curated list of human cancer genes, that also assigns genes as tumour suppressor genes and oncogenes. COSMIC also provides mutation types such as germline, somatic, or both. We also classified each tumour suppressor genes as being a gatekeeper gene or a caretaker gene according to the list provided by Tollis et al. (2020).

The copy number count of cancer related genes in mammals is this report is obtained differently from Tollis et al. (2020). To get the copy number of the abovementioned COSMIC genes in different mammal species two approaches were used. The first one included downloading the Ensembl CAFE species trees for all of the COSMIC genes. The Ensembl CAFE provides species trees for gene copy number counts. Ensembl CAFE data "gene gains and losses in each GeneTree are calculated in by starting from the number of gene copies in each species and using CAFE (Bie et al. 2006) to estimate how many genes existed in each lineage before a speciation event" cited from(Herrero et al. 2016). The second method included downloading the list of human COSMIC gene orthologs for each mammal species represented in the Ensembl database using BioMart (Kinsella et al. 2011) and counting the unique confident orthologs in each species, for each gene. The chosen approach is computationally much less intensive than the one used by Tollis et al. (2020) as it reuses the computational effort from Ensembl. As the supplementary material S1 indicates, the computational methods provide somewhat different CNV estimates. In our opinion, the Ensembl CAFE approach of calculating the gene gains and losses is superior to the approach of Tollis et al. (2020) as it takes into account the phylogenetic relationship of animals in CNV calculation.

Then the normalized copy number counts for both cancer gene lists (COSMIC Tier 1 and COSMIC Tier 1&2) and for both copy number count methods (CAFE and Ortholog) was calculated according to Tollis et al. (2020). See also code in Baines et al. (2021b).

The maximum length and lifespan data (as well as other parameters) were obtained from AnAge database Tacutu et al. 2018.
The phylogenetic tree for the mammal species together with branch lengths was obtained from www.timetree.org. Species that were missing in the timetree database were excluded from the analysis as phylogenetically informed regressions can’t be done without phylogenetic distances.
From all the mammal species that have a genome in Ensembl (95) only 73 have both adult weight and maximum lifespan given. We further excluded low and questionable data (quality assigned by anAge).
This retained 70 species.

Analysis on the Enseble CAFE mammal dataset

NB! All of the following graphs display **all individual species as data points (N)** and the regression line (with intervals) is calculated using the **lm()** function. However, the R2 and p value are the results from **phylogenetically informed regression** (function pgls from package caper). Hence, comparing the visuals on the plot and R2 and p values is somewhat misleading.

Lifespan vs size

First we will look if the maximum lifespan is related to maximum body size in all species. The underling hypothesis is that species who live longer also tend to be larger in log scale.

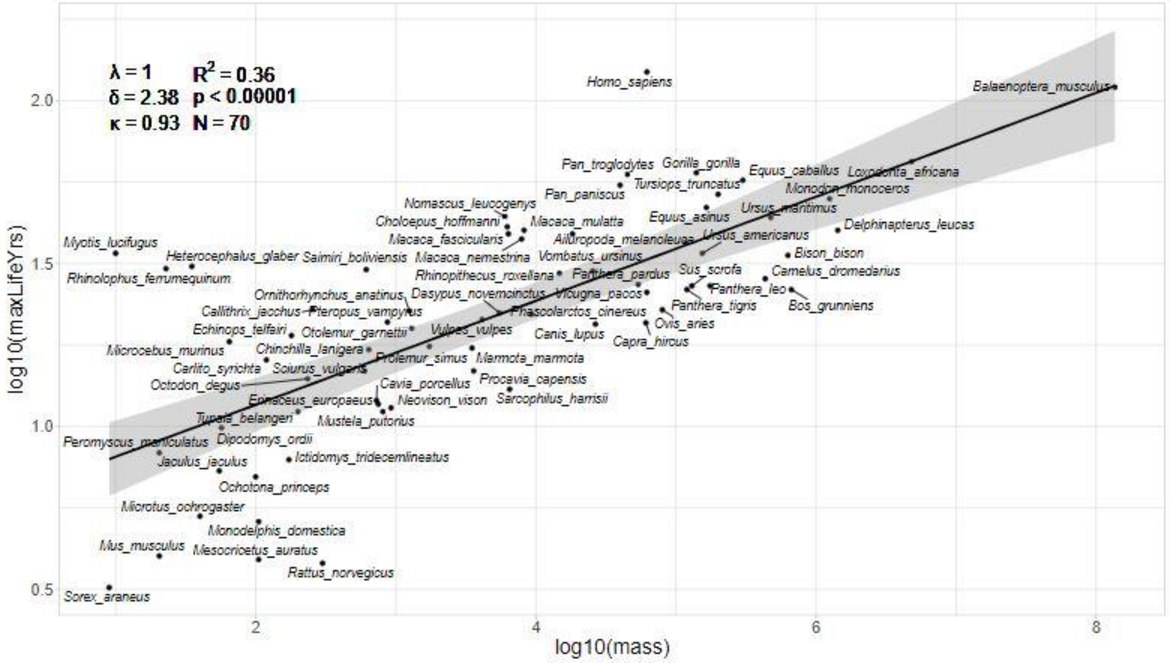

Figure S33. Linear regression between log transformed maximum body mass and maximum lifespan. Each point in the plot represents a species. The line and the confidence intervals depicted in the plot come from standard linear regression, the values R^2, p and N are from phylogenetically adjusted regression.
So the direction is the same as for in fish or in the dataset of Tollis et al. (2020). Just that we have more available data. Note how the human is a severe outlier.
From this correlation we calculated the longevity quotient according to Tollis et al. (2020).

Cancer gene counts

To see the correlations between types of different COSMIC gene counts we correlated the normalized copy number counts of different cancer gene measures.

Correlations between different cancer gene counts

First we looked to see if the obtained results depended on the method of getting copy number counts (CAFE vs ortholog) or subset of cancer genes (COSMIC tier 1 vs COSMIC tier 1&2).

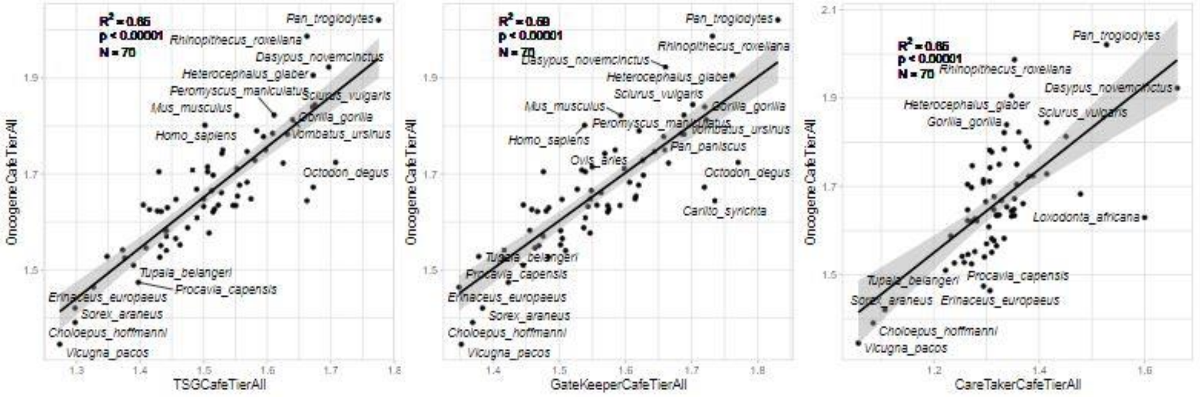

Figure S34. Linear regression between copy numbers of different subsets of tumour suppressor genes (all TSGs, gatekeeper genes and caretaker genes) and oncogenes. The copy numbers have been obtained using the **CAFE** approach and both COSMIC **Tier 1&2** genes are included. Each point in the plot represents a species. The line and the confidence intervals depicted in the plot come from standard linear regression, the values R^2, p and N are from phylogenetically adjusted regression.

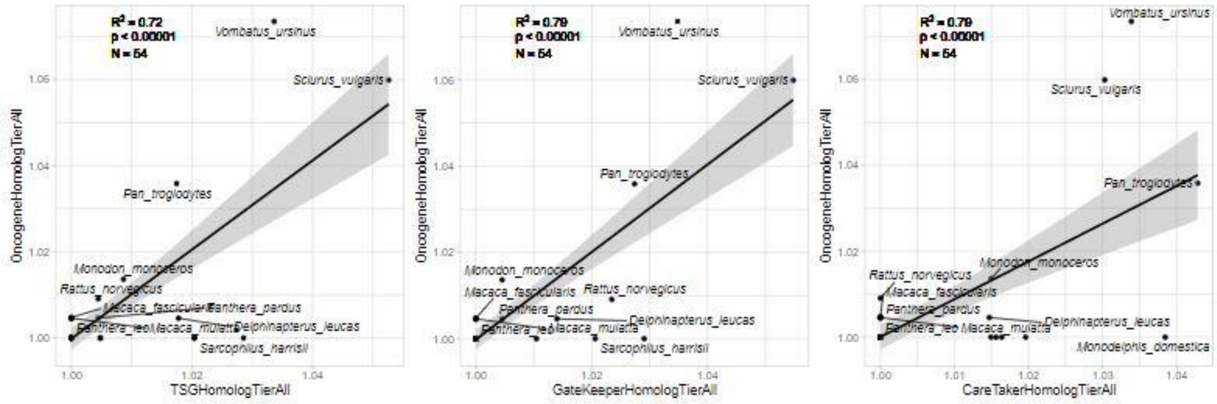

Figure S35. Linear regression between copy numbers of different subsets of tumour suppressor gene (all TSGs, gatekeeper genes and caretaker genes) and oncogenes. The copy numbers have been obtained using the **homolog** approach and both COSMIC **Tier 1&2** genes are included. Each point in the plot represents a species. The line and the confidence intervals depicted in the plot come from standard linear regression, the values R^2, p and N are from phylogenetically adjusted regression.

Looking at the two above plots it seems that the positive correlations between oncogene and tumors suppressor genes is present regardless of the method of getting cancer gene copy number counts. The same can be said about subset of genes used (plots not shown above but below and in subsequent chapters). The results are extremely similar for tier 1 and for both tiers combined.

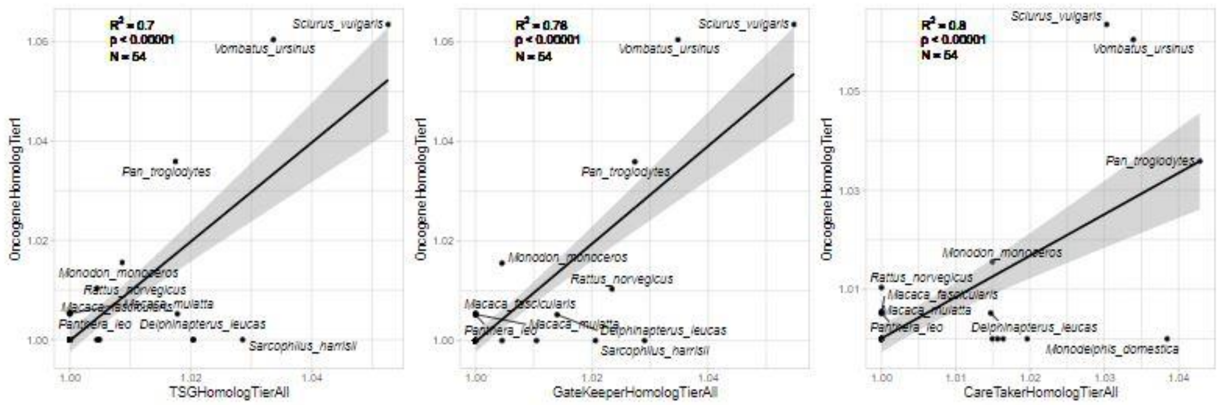

Figure S36. Linear regression between copy numbers of different subsets of tumour suppressor gene (all TSGs, gatekeeper genes and caretaker genes) and oncogenes. The copy number have been obtained using the **homolog** approach and COSMIC **Tier 1** genes are included. Each point in the plot represents a species. The line and the confidence intervals depicted in the plot come from standard linear regression, the values R^2, p and N are from phylogenetically adjusted regression.

However, the total number of species in the analysis is larger for CAFE as the ortholog approach failed to produce copy number counts for some species. So we chose CAFE to retain more species in the further analyses and tier 1 because this enables to include only the most studied and validated cancer genes.

Copy number variation (CNV) and lifepan

One central idea is that higher number of TSGs and lower number of oncogenes provides the basis to live longer. Let's correlate different copy number counts with maximum lifespan.

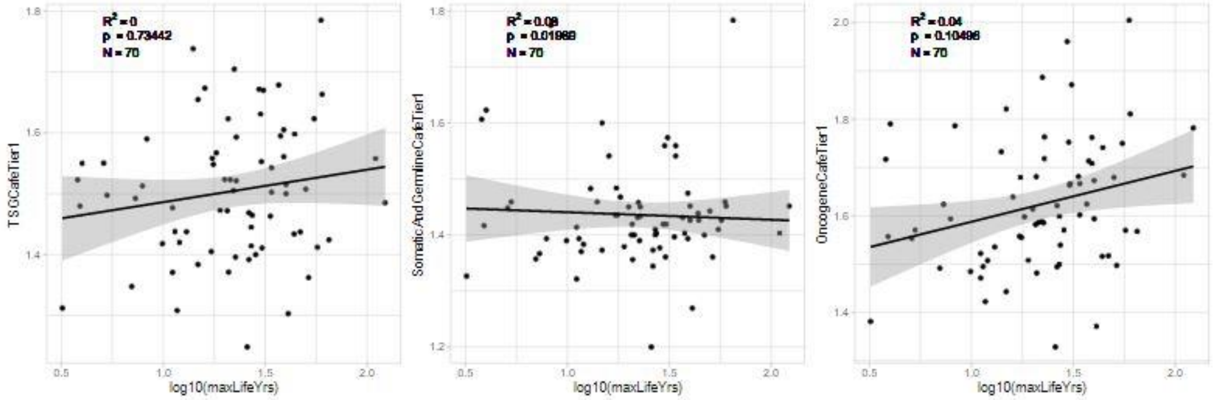

Figure S37. Linear regression between log maximum lifespan and copy numbers of different subsets of cancer related genes (TSG, SomaticAndGermline, Oncogene). The copy numbers have been obtained using the **CAFE** approach and only COSMIC **Tier 1** genes are included. Each point in the plot represents a species in the dataset. The line and the confidence intervals depicted in the plot come from standard linear regression, the values R^2, p and N are from phylogenetically adjusted regression.

So all of these correlations are non-significant. However as oncogenes and TSG's should have, in general, an opposite relationship to lifespan it is worthwhile to put both into the model.

Oncogene masked relationship with TSG copy numbers

Lets check, if the oncogene copy number masked relationship between lifespan and TSG copy numbers that we detected in the fish dataset, is also detectable in the mammal dataset.

Table S19. Results from the phylogenetically adjusted regression with the formula *log10(maxLifeYrs) ~ OncogeneCafeTier1 + TSGCafeTier1*. The lambda, kappa and delta values have been optimized using maximum likelihood if not 1. The copy numbers have been obtained using the **CAFE** approach and only COSMIC **Tier 1** genes are included.

	Estimate	SE	t	p		Estimate	SE	t	p	
(Intercept)	1.15	0.36	3.23	0.0019	**	1.26	0.35	3.56	7e-04	***
OncogeneCafeTier1	0.94	0.28	3.34	0.0014	**	0.99	0.29	3.38	0.0012	**
TSGCafeTier1	-0.94	0.33	-2.88	0.0053	**	-0.96	0.34	-2.83	0.0061	**
	kappa	lambda	delta			kappa	lambda	delta		
	1	1	1			0.91	1	1.19		

The results above suggest an inverted masked relationship in mammals and are similar to the results in Tollis et al. (2020) finding a positive correlation between lifespan and oncogene count.

Table S20. Results from the phylogenetically adjusted regression with the formula *log10(maxLifeYrs) ~ log10(mass) + log(Litter.Clutch.size) + log(Litters.Clutches.per.year) + , OncogeneCafeTier1 + TSGCafeTier1*. The lambda, kappa and delta values have been optimized using maximum likelihood. The copy numbers have been obtained using the **CAFE** approach and only COSMIC **Tier 1** genes are included.

	Estimate	SE	t	p		Estimate	SE	t	p	
(Intercept)	0.91	0.29	3.11	0.0029	**	0.67	0.29	2.3	0.025	*
log10(mass)	0.08	0.02	3.56	8e-04	***	0.08	0.02	3.71	5e-04	***
log(Litter.Clutch.size)	-0.16	0.04	-3.88	3e-04	***	-0.16	0.04	-3.86	3e-04	***
log(Litters.Clutches.per.year)	-0.03	0.04	-0.6	0.55		-0.06	0.04	-1.38	0.172	
OncogeneCafeTier1	0.97	0.23	4.17	1e-04	***	1.01	0.27	3.69	5e-04	***
TSGCafeTier1	-0.91	0.26	-3.47	0.001	**	-0.78	0.31	-2.54	0.014	*

	kappa	lambda	delta		kappa	lambda	delta
	1	1	1		1	0.98	3

This is true even if we take body size and reproductive effort into this model.
Let it be noted, that the CAFE approach of getting gene counts takes the species tree into account. So one might argue that in this case using standard linear regression to check, if the oncogene copy number masked relationship between lifespan and TSG copy numbers would be justified. However, the main reason for using phylogenetically adjusted model is to include the relative distance between speciation events (i.e.taking the branch lengths into account).

Humans

As the **human** in this dataset might be a confounding observation due to the fact it is the species all else is compared to and has extended lifespan let us exclude it.

Table S21. Results from the phylogenetically adjusted regression with the formula *log10(maxLifeYrs) ~ OncogeneCafeTier1 + TSGCafeTier1*. The lambda, kappa and delta values have been optimized using maximum likelihood. The copy numbers have been obtained using the **CAFE** approach and only COSMIC **Tier 1** genes are included. Homo sapiens is excluded.

	Estimate	SE	t	p		Estimate	SE	t	p	
(Intercept)	0.82	0.35	2.3	0.024	*	1.1	0.36	3.06	0.0032	**
OncogeneCafeTier1	0.6	0.29	2.09	0.041	*	0.62	0.28	2.22	0.03	*
TSGCafeTier1	-0.35	0.37	-0.96	0.34		-0.42	0.36	-1.15	0.25	
	kappa	lambda	delta			kappa	lambda	delta		
	1	1	1			1.06	1	1		

The human datapoint has a huge impact on the results.

Table S22. Results from the phylogenetically adjusted regression with the formula *log10(maxLifeYrs) ~ log10(mass) + log(Litter.Clutch.size) + log(Litters.Clutches.per.year) +* , *OncogeneCafeTier1 + TSGCafeTier1*. The lambda, kappa and delta values have been optimized using maximum likelihood. The copy numbers have been obtained using the **CAFE** approach and only COSMIC **Tier 1** genes are included. Homo sapiens is excluded.

	Estimate	SE	t	p		Estimate	SE	t	p	
(Intercept)	0.56	0.27	2.08	0.042	*	0.58	0.25	2.3	0.025	*
log10(mass)	0.08	0.02	4.03	2e-04	***	0.08	0.02	4.21	<0.0001	***
log(Litter.Clutch.size)	-0.16	0.04	-4.54	<0.0001	***	-0.17	0.04	-4.68	<0.0001	***

log(Litters.Clutches.per.year)	-0.03	0.04	-0.86	0.4		-0.04	0.04	-1	0.32	
OncogeneCafeTier1	0.58	0.22	2.64	0.011	*	0.58	0.22	2.61	0.012	*
TSGCafeTier1	-0.26	0.27	-0.94	0.35		-0.27	0.28	-0.97	0.34	
	kappa	lambda	delta			kappa	lambda	delta		
	1	1	1			1.2	0.99	2.02		

Clearly, the human data point has a huge impact on the observed results.

Body size vs cancer gene counts

To reproduce the plots in fig 4 of Tollis et al (2020) we correlated the body size measures with normalized cancer related gene counts.

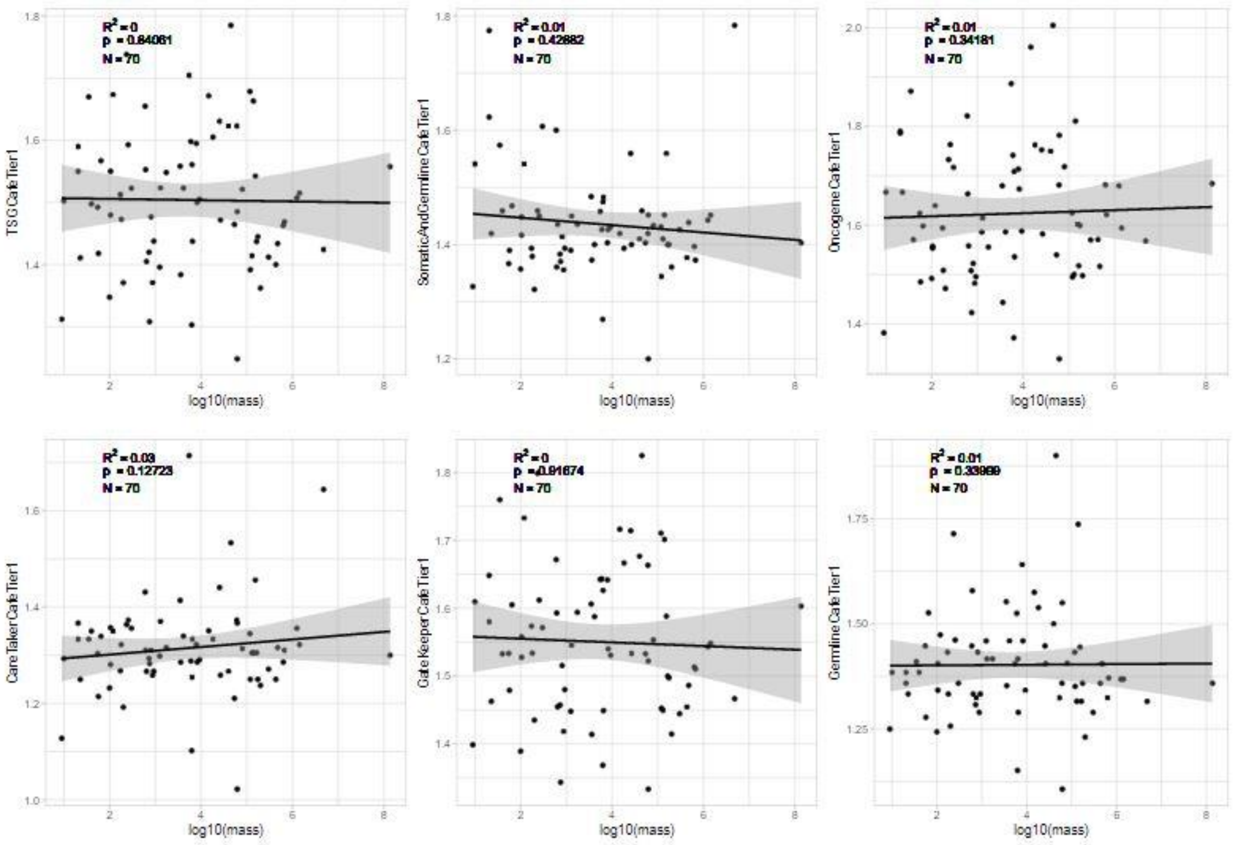

Figure S38. Linear regression between log maximum body size and copy numbers of different subsets of cancer related genes (TSG, SomaticAndGermline, Oncogene, CareTaker, GateKeeper, Germline). The copy numbers have been obtained using the **CAFE** approach and only COSMIC **Tier 1** genes are included. Each point in the plot represents a species in the dataset. The line and the confidence intervals depicted in the plot come from standard linear regression, the values R^2, p and N are from phylogenetically adjusted regression.

All of the results are similarly non-significant.

Longevity Quotient (LQ) vs cancer gene copy number counts

Next we looked at the longevity quotient results. The LQ was calculated as in the article of Tollis et al (2020).

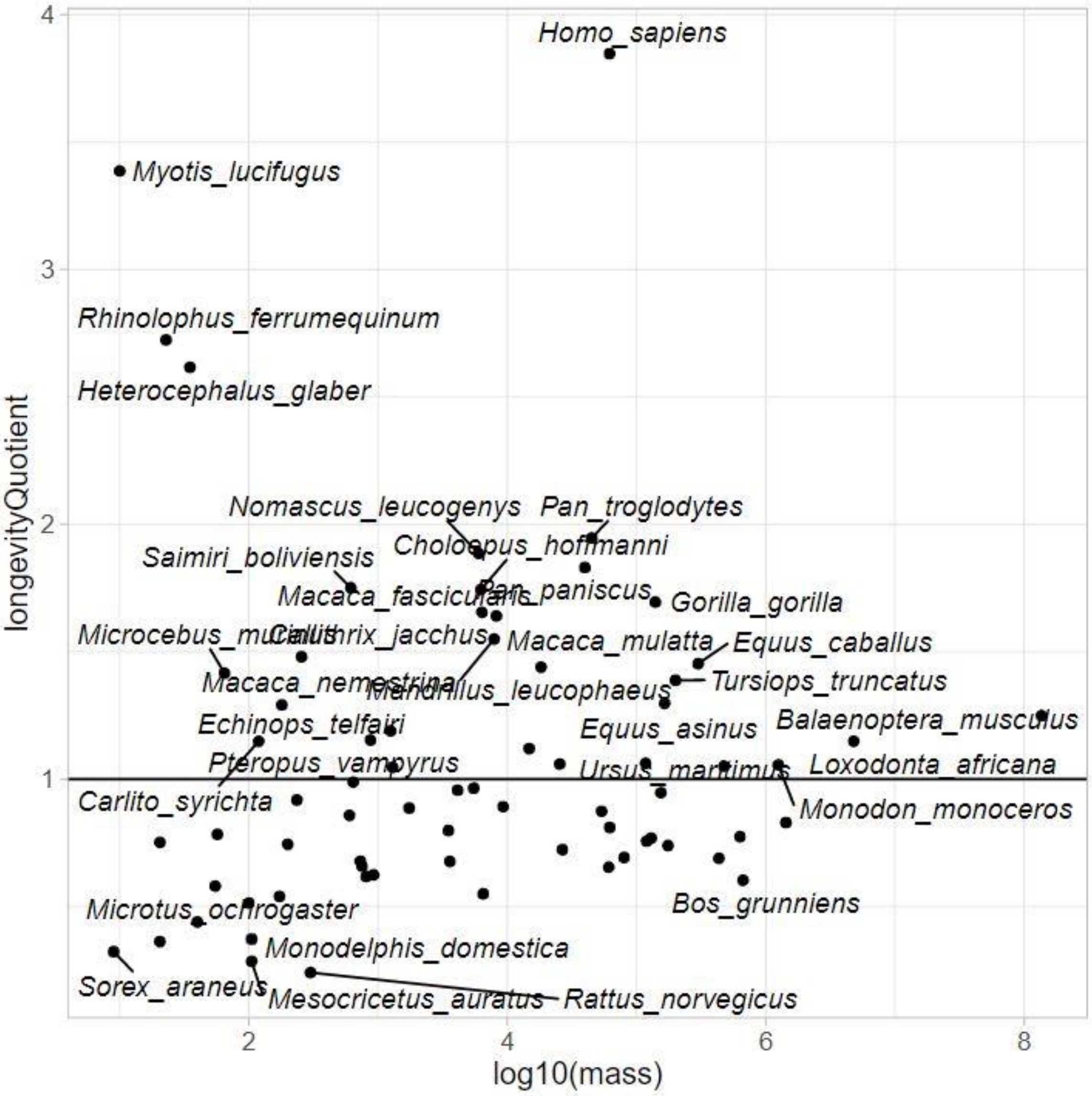

Figure S39. A plot between log transformed body mass and longevity quotient (LQ). Each point in the plot represents a species. To calculate the LQ we used the lifespan to mass model (i.e. model from figure 1) similarly to Tollis et al. (2020).

Clearly human stands out living longer than expected for its body size.

Next we plot the same correlations between numbers of copies of genes and LQ, as we did for maximum body size and lifespan.

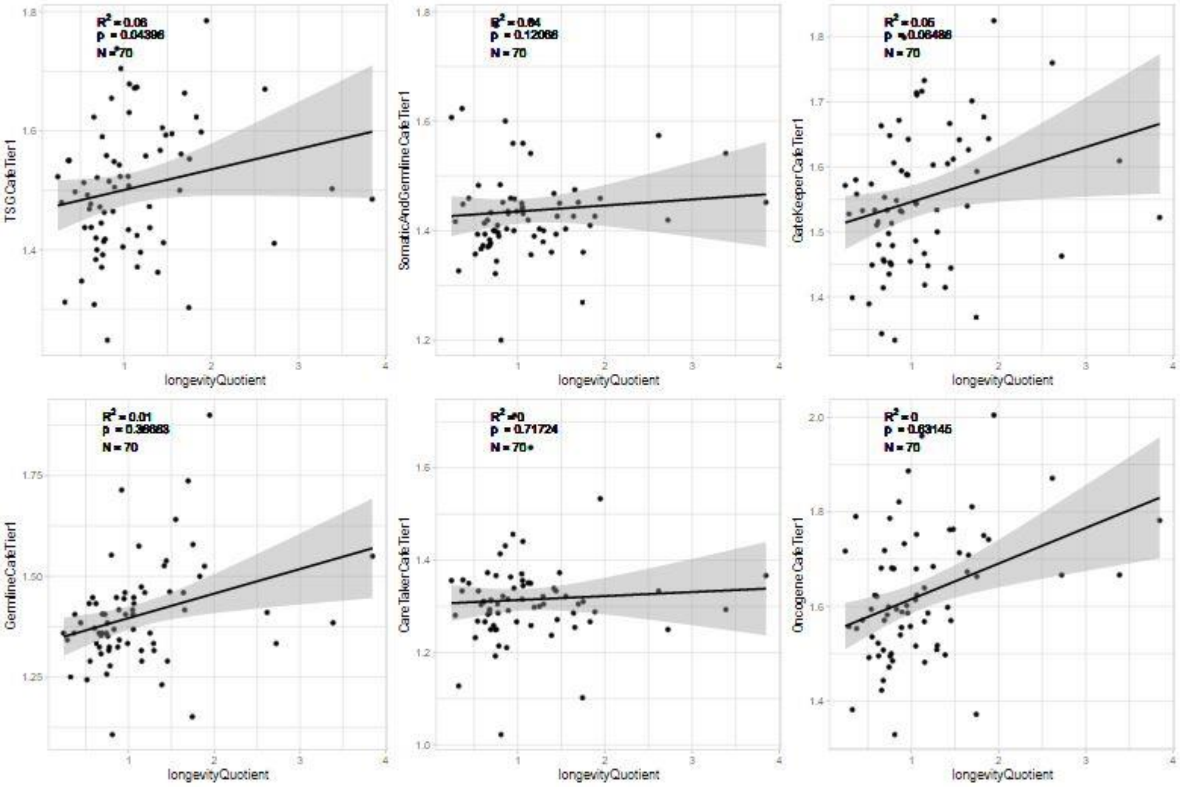

Figure S40. Linear regression between longevity quotient (LQ) and copy numbers of different subsets of cancer related genes (TSG, SomaticAndGermline, GateKeeper, Germline, CareTaker, Oncogene). The copy numbers have been obtained using the **CAFE** approach and only COSMIC **Tier 1** genes are included. The LQs have been obtained using the same method as in Tollis et al. (2020). Each point in the plot represents a species in the dataset. The line and the confidence intervals depicted in the plot come from standard linear regression, the values R^2, p and N are from phylogenetically adjusted regression.

None of the correlations with copy numbers and LQ are very strong.

Oncogene masked relationship with TSG copy numbers

Finally let's look if the oncogene copy numbers masked relationship between LQ and TSG copy numbers that we detected in the fish dataset is also detectable in the mammal dataset.

Table S23. Results from the phylogenetically adjusted regression with the formula *longevityQuotient ~ OncogeneCafeTier1 + TSGCafeTier1*. The lambda, kappa and delta values have been optimized using maximum likelihood. The copy numbers have been obtained using the **CAFE** approach and only COSMIC **Tier 1** genes are included.

	Estimate	SE	t	p		Estimate	SE	t	p	
(Intercept)	2.22	1.05	2.12	0.038	*	0.53	0.92	0.58	0.57	
OncogeneCafeTier1	3.18	0.82	3.87	3e-04	***	3.18	0.9	3.52	8e-04	***

TSGCafeTier1	-4.27	0.96	-4.44	<0.0001	***	-3.09	1.03	-3.01	0.0037	**
	kappa	lambda	delta			kappa	lambda	delta		
	1	1	1			0.71	0.97	3		

So again, the results suggest the opposite relationship than observed in fish. Let's exclude the human datapoint as it might be misleading in this context. First the maximum human lifespan is inflated due to much larger sample size and much better hospital care compared to other animals. Secondly, as the dataset is compiled in comparison to human cancer gene count the copy numbers of genes for humans is effectively 1.

Table S24. Results from the phylogenetically adjusted regression with the formula *longevityQuotient ~ OncogeneCafeTier1 + TSGCafeTier1*. The lambda, kappa and delta values have been optimized using maximum likelihood. The copy numbers have been obtained using the **CAFE** approach and only COSMIC **Tier 1** genes are included. Homo sapiens is excluded.

	Estimate	SE	t	p		Estimate	SE	t	p	
(Intercept)	0.12	0.74	0.16	0.88		0.56	0.63	0.9	0.37	
OncogeneCafeTier1	1.07	0.6	1.77	0.082	.	0.83	0.46	1.81	0.075	.
TSGCafeTier1	-0.56	0.77	-0.72	0.47		-0.53	0.62	-0.86	0.39	
	kappa	lambda	delta			kappa	lambda	delta		
	1	1	1			1.54	1	1.4		

After removing the human data the result becomes again non-significant.

Analysis on the mammal dataset combined by Tollis et al.(2020)

The section aims to verify if the masked relationsip of TSG and Oncogene link to lifespan holds also in data put together by Tollis et al. (2020). To be more conservative phylogenetically informed regression is used similar to Tollis et al. 2020.

Read data

There are 63 species present in the data frame by Tollis et al. (2020).

Lifespan relation to cancer genes

The first plot plots the phylogenetically informed regression.

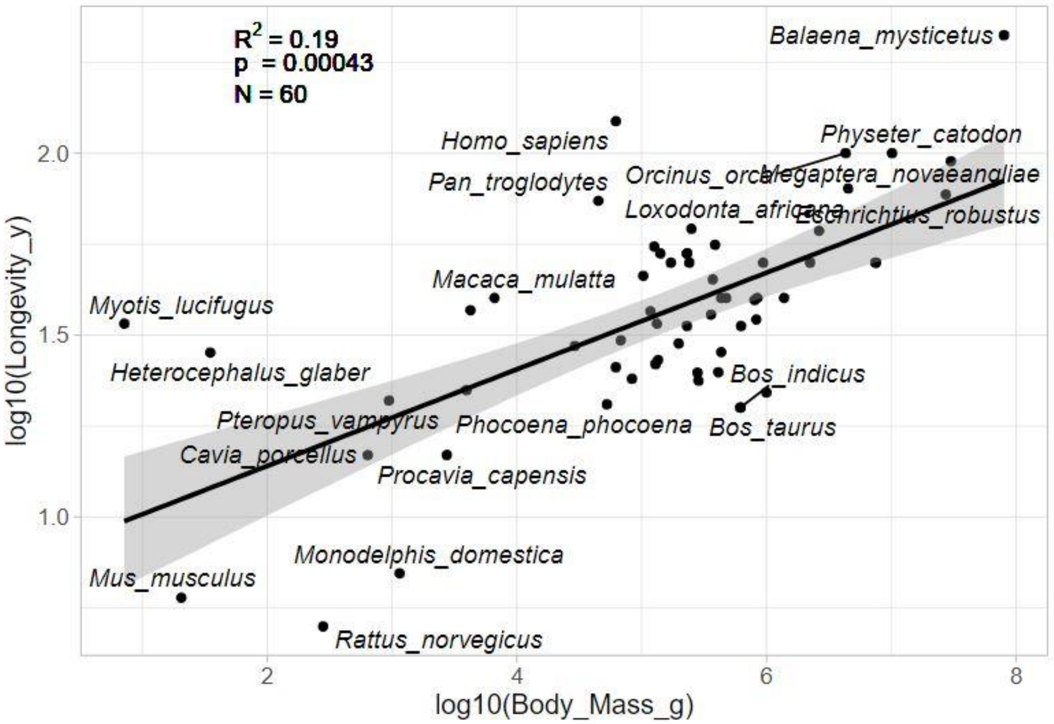

Figure S41. Linear regression between log transformed maximum body mass and maximum lifespan. Each point in the plot represents a species. The line and the confidence intervals depicted in the plot come from standard linear regression, the values R^2, p and N are from phylogenetically adjusted regression. This is the same plot as in Tollis et al. (2020).

Cancer gene copy numbers and lifespan

Table S25. Results from the phylogenetically adjusted regression with the formula *log10(Longevity_y) ~ Total_TSGs_Normalized + Total_Oncogenes_Normalized.* The lambda, kappa and delta values are fixed at 1. The data is from Tollis et al. (2020).

	Estimate	SE	t	p	
(Intercept)	1.04	0.53	1.95	0.056	.
Total_TSGs_Normalized	-0.21	0.31	-0.67	0.51	
Total_Oncogenes_Normalized	0.26	0.2	1.32	0.192	
	kappa	lambda	delta		
	1	1	1		

Table S26. Results from the phylogenetically adjusted regression with the formula log10(Longevity_y) ~ Total_TSGs_Normalized + Total_Oncogenes_Normalized. The lambda, kappa and delta values have been optimized using maximum likelihood. The data is from Tollis et al. (2020).

	Estimate	SE	t	p	
(Intercept)	0.54	0.41	1.31	0.196	
Total_TSGs_Normalized	0.86	0.34	2.52	0.014	*
Total_Oncogenes_Normalized	-0.34	0.2	-1.76	0.083	.
	kappa	lambda	delta		
	0.77	0.91	0.97		

Phylogenetically informed regression on the Tollis et al. (2020) dataset does not confirm, that species with longer lifespan have overall more copies of known TSG genes and less copies of oncogene genes if λ, δ and κ are fixed at 1 but display a similar pattern to fish if all three (or only λ or κ) have been optimized by maximum likelihood.

Table S27. Results from the phylogenetically adjusted regression with the formula *log10(Longevity_y) ~ Log_Body_Mass + Total_TSGs_Normalized + , Total_Oncogenes_Normalized.* The lambda, kappa and delta values have been optimized using maximum likelihood if not 1. The data is from Tollis et al. (2020).

	Estimate	SE	t	p		Estimate	SE	t	p	
(Intercept)	0.19	0.49	0.38	0.7		0.15	0.34	0.43	0.67	
Log_Body_Mass	0.18	0.04	4.61	<0.0001	***	0.15	0.03	5.46	<0.0001	***
Total_TSGs_Normalized	-0.44	0.27	-1.63	0.109		0.36	0.29	1.24	0.22	
Total_Oncogenes_Normalized	0.51	0.18	2.85	0.0061	**	0	0.17	0.02	0.98	
	kappa	lambda	delta			kappa	lambda	delta		
	1	1	1			1.11	0.9	3		

However, adding body mass to the model unlinks lifespan from TSG or oncogene copy numbers.

Using Ensembl only genomes

It is interesting to note however, that if to keep only these mammals with a genome assembly available in Ensembl (Having a genome in Ensembl may be considered as having a genome of rather good quality). The phylogenetically informed regression reveals the same masked relationship in the Tollis et al. (2020) dataset that we discovered in our fish dataset. See below:

Table S28. Results from the phylogenetically adjusted regression with the formula *log10(Longevity_y) ~ Total_TSGs_Normalized + Total_Oncogenes_Normalized*. The lambda, kappa and delta values have been optimized using maximum likelihood if not 1. The data is from Tollis et al. (2020).

	Estimate	SE	t	p		Estimate	SE	t	p	
(Intercept)	0.61	0.43	1.42	0.166		0.78	0.4	1.96	0.059	.
Total_TSGs_Normalized	1	0.36	2.81	0.0087	**	1.23	0.37	3.36	0.0022	**
Total_Oncogenes_Normalized	-0.48	0.22	-2.17	0.038	*	-0.65	0.2	-3.26	0.0028	**

	kappa	lambda	delta		kappa	lambda	delta
	1	1	1		1.4	0.97	3

So in this subset of data species with longer lifespan have less oncogenes and more TSG’s and the relationship is more likely true for TSG’s. Notably some of the largest mammals (whales) are missing in this dataset. See the plot below.

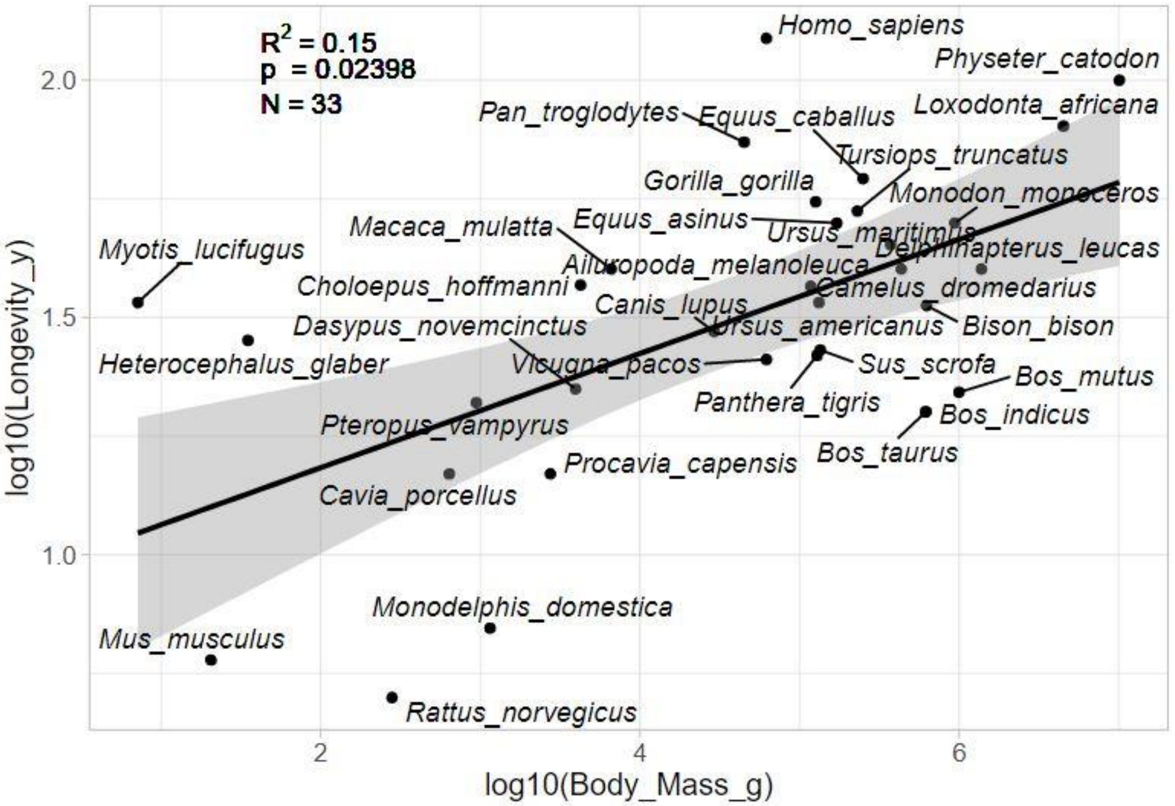

Figure S42. Linear regression between log transformed maximum body mass and maximum lifespan. Each point in the plot represents a species. The line and the confidence intervals depicted in the plot come from standard linear regression, the values R^2, p and N are from phylogenetically adjusted regression. This is the same plot as in Tollis et al. (2020) except the species that have a genome in Ensembl.

Table S29. Results from the phylogenetically adjusted regression with the formula *log10(Longevity_y) ~ Log_Body_Mass + Total_TSGs_Normalized + , Total_Oncogenes_Normalized*. The lambda, kappa and delta values have been optimized using maximum likelihood if not 1. The data is from Tollis et al. (2020).

	Estimate	SE	t	p		Estimate	SE	t	p	
(Intercept)	0.17	0.46	0.37	0.72		0.31	0.42	0.75	0.46	
Log_Body_Mass	0.09	0.04	2.06	0.048	*	0.09	0.04	2.36	0.025	*

Total_TSGs_Normalized	0.8	0.35	2.28	0.03	*	0.92	0.36	2.54	0.017	*
Total_Oncogenes_Normalized	-0.28	0.23	-1.22	0.23		-0.37	0.22	-1.73	0.094	.
	kappa	lambda	delta			kappa	lambda	delta		
	1	1	1			1.34	0.96	3		

The relationship for TSGs holds also if taking body mass into account. In previous sections we checked the existence of the masked relationship in the full Ensembl CAFE dataset of mammals (using CNV calculations from this paper). It's notable that the overlap between the mammal genomes available in both Ensembl and the genomes used in Tollis et al. (2020) datasets is relatively small (33 species are in both datasets).

Cancer gene copy numbers and longevity quotient

Finally lets look if the overlapping dataset (33 species) display a masked relationship between TSG's and LQ as we saw in the fish dataset.

Table S30. Results from the phylogenetically adjusted regression with the formula *Longevity_Quotient ~ Total_TSGs_Normalized + Total_Oncogenes_Normalized*. The lambda, kappa and delta values have been optimized using maximum likelihood if not 1. The data is from Tollis et al. (2020).

	Estimate	SE	t	p	Estimate	SE	t	p
(Intercept)	-0.08	0.68	-0.11	0.91	0.42	0.48	0.87	0.39
Total_TSGs_Normalized	0.26	0.56	0.46	0.65	-0.11	0.36	-0.31	0.76
Total_Oncogenes_Normalized	0.36	0.35	1.04	0.31	0.43	0.29	1.46	0.156
	kappa	lambda	delta		kappa	lambda	delta	
	1	1	1		1.63	1	3	

The overlapping dataset of 33 mammal species, does not indicate that longevity quotient is affected by the TSG and Oncogene counts. So we also failed to demonstrate a clear masked relationship (that we observed in our fish genomes dataset) in the Tollis et al. (2020) dataset of mammals.

Conclusions

We were unable to demonstrate a masked relationship with the number of copies of TSGs and oncogenes and maximum lifespan in mammal species from Ensembl (104) using the CAFE gene counts and age and size data from anAge. On the contrary, an inverted relationship can be observed if the human datapoint is included into the model. However by removing the human from the data,

because the data collection for human lifespan and cancer gene count is different from other mammals, these results become less reliable.

One possible explanation why the masked relationship between the number of copies of TSGs and lifespan does not hold for mammals is the relatively small phylogenetic distance between different mammal species, compared to the distance differences between fish species. It might be that such a relationship emerges only in large scale. Another possible explanation is that the cancer genes that have an ortholog in fish are the most conserved and/or important ones for lifespan.

References

De Bie T, Cristianini N, Demuth JP, Hahn MW, Leuven KU. 2006. CAFE: a computational tool for the study of gene family evolution. Bioinformatics. 22:1269–1271. (May 15, 2006): 1269–71. *https://doi.org/10.1093/bioinformatics/btl097*.

Tacutu R, Barardo D, Craig T. 2017. Human Ageing Genomic Resources: New and updated databases . Artic. Nucleic Acids Res. 46:D1083-D1090., no. D1 (January 4, 2018). *https://doi.org/10.1093/nar/gkx1042*.

Herrero J, Muffato M, Beal K, Fitzgerald S, Gordon L, Pignatelli M, Vilella AJ, Searle SMJ, Amode R, Brent S, et al. 2016. Ensembl comparative genomics resources. Database 2016, no. bav096 (January 1, 2016). *https://doi.org/10.1093/database/bav096*.

Kinsella RJ, Kä Hä Ri A, Haider S, Zamora J, Proctor G, Spudich G, Almeida-King J, Staines D, Derwent P, Kerhornou A, et al. 2011. Ensembl BioMarts: a hub for data retrieval across taxonomic space. Database 2011. (0):bar030.

Tollis M, Schneider-Utaka AK, Maley CC. 2020. The Evolution of Human Cancer Gene Duplications across Mammals. Mol. Biol. Evol. 37:2875–2886.

Sondka Z, Bamford S, Cole CG, Ward SA, Dunham I, Forbes SA. 2018. The COSMIC Cancer Gene Census: describing genetic dysfunction across all human cancers. Nat. Rev. Cancer 18:696–705.

S7. Sources for age data

Age data was taken from Fishbase and AnAge databases, when available. For the remaining species, other data sources were used. Details can be seen from Table S31.

Table S31. Sources of the age data

**Species**	**Age source**	**Source clarification**	**Date**
Acanthochromis_polyacanthus	article	Kingsford and Hughes, 2005	
Amphilophus_citrinellus	animal_world	*https://animal-world.com/encyclo/fresh/cichlid/MidasCichlid.php#:~:text=Though%20both%20males%20and%20females,more%20if%20well%20cared%20for.*	June 16, 2021
Amphiprion_ocellaris	other	*https://phys.org/news/2019-04-nemo-animal-aging.html*	March 1, 2021

Amphiprion_percula	article	Buston and García, 2007	
Anabas_testudineus	anage		
Astatotilapia_calliptera	other	https://tropical-fish-keeping.com/eastern-happy-astatotilapia-calliptera.html	June 10, 2021
Astyanax_mexicanus_Astyanax_mexicanus-1.0.2	fishbase		
Astyanax_mexicanus_Astyanax_mexicanus-2.0	fishbase		
Betta_splendens	anage		
Carassius_auratus	anage		
Clupea_harengus	anage		
Cottoperca_gobio	article	*https://www.frontiersin.org/articles/10.3389/fmars.2020.00421/full*	March 1, 2021
Cyclopterus_lumpus	fishbase		
Cynoglossus_semilaevis	article	Meng and Ren, 1988	
Cyprinodon_variegatus	dissertation	Foster, 1967	
Cyprinus_carpio_common_carp_genome	anage		
Danio_rerio	anage		
Dicentrarchus_labrax	fishbase		

Echeneis_naucrates	article	Bachman et al. 2018	
Electrophorus_electricus	anage		
Esox_lucius	anage		
Fundulus_heteroclitus	fishbase		
Gadus_morhua	anage		
Gambusia_affinis	anage		
Gasterosteus_aculeatus	anage		
Haplochromis_burtoni	article	Hoffmann et al. 1999	
Hippocampus_comes	article	Morgan, 2007	
Hucho_hucho	fishbase		
Ictalurus_punctatus	fishbase		
Kryptolebias_marmoratus	article	Rossi et al. 2019	
Labrus_bergylta	anage		
Larimichthys_crocea	article	Chen et al. 2020	
Lates_calcarifer	article	Balston, 2009	
Lepisosteus_oculatus	anage		
Mastacembelus_armatus	animal_world	https://animal-world.com/encyclo/fresh/Eels/ZigZagEel.php#:~:text=Spiny%20eels%20have%20a%20lifespan,series%20of%20irregular%20dark%20markings.	May 18, 2021

Maylandia_zebra	animal_world	https://animal-world.com/encyclo/fresh/cichlid/RedZebra.php#:~:text=be%20closely%20related.-,Size%20of%20fish%20%2D%20inches%3A%203.9%20inches%20(10.01%20cm),10%20years%20with%20proper%20care.	May 18, 2021
Mola_mola	national_geographic	*https://www.nationalgeographic.com/animals/fish/facts/ocean-sunfish*	May 18, 2021
Monopterus_albus	article	Yang and Xiong, 2010	
Myripristis_murdjan			
Neogobius_melanostomus	fishbase		
Neolamprologus_brichardi	animal_world	https://animal-world.com/encyclo/fresh/cichlid/brichardi.php#:~:text=under%20the%20eyes.-,Size%20of%20fish%20%2D%20inches%3A%205.1%20inches%20(13.00%20cm),10%20years%20with%20proper%20care.	May 18, 2021
Nothobranchius_furzeri	anage		
Oncorhynchus_kisutch	anage		
Oncorhynchus_mykiss	anage		
Oncorhynchus_tshawytscha	anage		
Oreochromis_niloticus	anage		
Oryzias_latipes_ASM223467v1	anage	Egami and Etoh, 1969	
Parambassis_ranga	article	Ishikawa and Tachihara, 2012	

Periophthalmus_magnuspinnatus	article	Jaafar and Murdy, 2017	
Poecilia_formosa	article	Loewe and Lamatsch, 2008	
Poecilia_reticulata	anage		
Pygocentrus_nattereri	animal_world	https://animal-world.com/encyclo/fresh/characins/RedPiranha.php#:~:text=Lifespan%3A%2020%20years%20%2D%20They%20generally,live%20for%20over%2020%20years.	May 19, 2021
Salarias_fasciatus	animal_world	https://animal-world.com/encyclo/marine/Blennies/LawnmowerBlenny.php	May 19, 2021
Salmo_salar	anage		
Salmo_trutta	anage		
Sander_lucioperca	fishbase		
Scleropages_formosus	gov_website	https://www.fws.gov/fisheries/ANS/erss/uncertainrisk/ERSS-Scleropages-formosus-Final-Dec2019.pdf	May 19, 2021
Scophthalmus_maximus	anage		
Seriola_dumerili	**fishbase**		
Seriola_lalandi_dorsalis	**fishbase**		
Sparus_aurata	anage		
Takifugu_rubripes	fishbase		
Tetraodon_nigroviridis	animal_world	https://animal-world.com/encyclo/fresh/Puffers/GreenSpottedPuffer.php	May 19, 2021
Latimeria_chalumnae	anage		

Petromyzon_marinus	fishbase
Callorhinchus_milii	anage

References:

Bachman, B.A., Kraus, R., Peterson, C.T., Grubbs, R.D. and Peters, E.C., 2018. Growth and reproduction of Echeneis naucrates from the eastern Gulf of Mexico. Journal of Fish Biology, 93:755-758.
Balston, J., 2009. Short-term climate variability and the commercial barramundi (Lates calcarifer) fishery of north-east Queensland, Australia. Marine and Freshwater Research, 60:912-923.
Buston, P.M. and García, M.B., 2007. An extraordinary life span estimate for the clown anemonefish Amphiprion percula. Journal of Fish Biology, 70:1710-1719.
Chen, Y., Huang, W., Shan, X., Chen, J., Weng, H., Yang, T. and Wang, H., 2020. Growth characteristics of cage-cultured large yellow croaker Larimichthys crocea. Aquaculture Reports, 16:100242.
Egami, N. and Etoh, H., 1969. Life span data for the small fish, Oryzias latipes. Experimental gerontology, 4:127-129.
Foster, N.R. 1967. Comparative studies on the biology of killifishes (Pisces: Cyprinodontidae). Ph.D. diss., Cornell Univ. Ithaca, N.Y., 369.
Hofmann, H.A., Benson, M.E. and Fernald, R.D., 1999. Social status regulates growth rate: consequences for life-history strategies. Proceedings of the National Academy of Sciences, 96:14171-14176.
Ishikawa, T. and Tachihara, K., 2012. Reproductive biology, growth, and age composition of non-native Indian glassy fish Parambassis ranga (Hamilton, 1822) in Haebaru Reservoir, Okinawa-jima Island, southern Japan. Journal of Applied Ichthyology, 28:231-237.
Jaafar, Z. and Murdy, E.O. eds., 2017. Fishes out of water: biology and ecology of mudskippers. CRC Press.
Kingsford, M.J. and Hughes, J.M., 2005. Patterns of growth, mortality, and size of the tropical damselfish Acanthochromis polyacanthus across the continental shelf of the Great Barrier Reef. Fishery Bulletin, 103:561-573.
Loewe, L. and Lamatsch, D.K., 2008. Quantifying the threat of extinction from Muller's ratchet in the diploid Amazon molly (Poecilia formosa). BMC Evolutionary Biology, 8:1-20.
Meng, T. and Ren, S., 1988. Age and growth of Cynoglossus semilaevis Günther in the Bohai Sea. Mar. Fish. Res., 9:173-183.
Morgan, S.K., 2007. The ontogenetic ecology and conservation of exploited tropical seahorses (Doctoral dissertation, McGill University).
Rossi, G.S., Cochrane, P.V., Tunnah, L. and Wright, P.A., 2019. Ageing impacts phenotypic flexibility in an air-acclimated amphibious fish. Journal of Comparative Physiology B, 189:567-579.
Yang, M.S. and Xiong, B.X., 2010. Age and growth of Monopterus albus Zuiew, 1793 (Synbranchidae). Journal of Applied Ichthyology, 26:488-490.
